# Including residual contact information into replica-exchange MD simulations significantly enriches native-like conformations

**DOI:** 10.1371/journal.pone.0242072

**Published:** 2020-11-16

**Authors:** Arthur Voronin, Marie Weiel, Alexander Schug

**Affiliations:** 1 Steinbuch Centre for Computing, Karlsruhe Institute of Technology, Eggenstein-Leopoldshafen, Germany; 2 Department of Physics, Karlsruhe Institute of Technology, Karlsruhe, Germany; 3 Institute for Advanced Simulation, Jülich Supercomputing Center, Jülich, Germany; 4 Faculty of Biology, University of Duisburg-Essen, Duisburg, Germany; Weizmann Institute of Science, ISRAEL

## Abstract

Proteins are complex biomolecules which perform critical tasks in living organisms. Knowledge of a protein’s structure is essential for understanding its physiological function in detail. Despite the incredible progress in experimental techniques, protein structure determination is still expensive, time-consuming, and arduous. That is why computer simulations are often used to complement or interpret experimental data. Here, we explore how *in silico* protein structure determination based on replica-exchange molecular dynamics (REMD) can benefit from including contact information derived from theoretical and experimental sources, such as direct coupling analysis or NMR spectroscopy. To reflect the influence from erroneous and noisy data we probe how false-positive contacts influence the simulated ensemble. Specifically, we integrate varying numbers of randomly selected native and non-native contacts and explore how such a bias can guide simulations towards the native state. We investigate the number of contacts needed for a significant enrichment of native-like conformations and show the capabilities and limitations of this method. Adhering to a threshold of approximately 75% true-positive contacts within a simulation, we obtain an ensemble with native-like conformations of high quality. We find that contact-guided REMD is capable of delivering physically reasonable models of a protein’s structure.

## Introduction

Knowledge of protein structures is crucial for understanding their various functions within living organisms and the biological processes they take part in. Structural knowledge is also critical in related fields such as pharmacology to understand pathogenesis on a molecular level as an essential prerequisite to effective drug design. Both protein structure and function are intrinsically encoded in the corresponding amino acid sequence [1–3]. Over the past years, experimental sequencing techniques have become exceptionally efficient and lead to fast growing sequence databases, e.g., GenBank [4] and UniProt [5]. In contrast, experimental structure determination using high-resolution X-ray crystallography or NMR spectroscopy is comparably time-consuming and expensive. Some other experimental techniques, e.g., small-angle X-ray scattering, Förster resonance energy transfer, and cryogenic electron microscopy, are less involved but provide ambiguous structural information of lower spatial resolution only. With proteins being nm-sized objects, molecular structures can only be observed indirectly. Measured data have to be interpreted carefully, which is why they commonly are complemented by computer simulations [6–8].

Over the past years, a broad variety of computational structure-prediction methods for inferring a three-dimensional protein structure from its amino acid sequence evolved, ranging from algorithms for “blind” or *de novo* predictions using Monte-Carlo or physics-based biomolecular simulations to algorithms transferring structure information from known homologous proteins. Today many web servers exist for calculation or prediction of additional information from sequence data, which can be integrated into simulations to further improve the model quality of proteins. They often also provide a complete automated workflow for structure prediction. Each web server specializes in different aspects, e.g. the Robetta server [9] mainly utilizes homology modeling and *ab initio* fragment assembly in Rosetta. Meanwhile, RaptorX [10, 11] focuses on machine learning and is capable of predicting secondary and tertiary structures as well as contact or distance maps, among many other things. Every other year, a new round of the Critical Assessment of Structure Prediction (CASP [12]) is held, in which the current state of the art for protein structure prediction solely reliant on sequence information and structure refinement methods are evaluated. Novel purely data-driven approaches using neural networks have recently shown to be capable of predicting high-quality structures [13, 14]. A drawback of such methods is that they typically lack insight into the physical processes driving structure adoption and cannot be easily complemented by experimental information. Depending on the method applied, local structural motifs are often less resolved [13] and could benefit from additional refinement. Physics-driven approaches are particularly suitable for this and based on specific energy functions called force fields. Using molecular-dynamics simulations, Lindorff et al. demonstrated current force fields to be sufficiently accurate to reversibly fold proteins starting from unfolded conformations [15, 16]. Still, the computational cost of such *de novo* folding simulations is extremely high. To date, simulations on the millisecond timescale can only be performed on specialized supercomputers like Anton [17]. Alternatively, simulations can be guided towards target structures or ensembles by including experimental information via an energetic bias based on measured data [18–21]. This bias is intended to smoothen the often frustrated energy landscape with many competing minima separated by high barriers. At the same time, computational costs are lowered due to the reduced sampling space. Using the example of ubiquitin, Raval et al. examined the extent to which the use of low-resolution information in the form of residue-residue contacts can speed up the determination of protein structure in plain all-atom MD simulations starting from extended conformations [22]. Introducing a flat-bottom harmonic potential for different numbers of randomly chosen native contacts, they found a significantly accelerated convergence to near-native structures even for a rather small number of restraints compared to simulations without contact bias. In light of these results, the questions arises whether one can further decrease computational demands by enhanced sampling techniques [23–25].

Applying bias potentials derived from different sets of error-ridden contact information, we here explore to what extent such information helps guiding replica-exchange molecular dynamics (REMD) towards the native fold. Contact information about adjacent amino acids can be obtained from different sources, e.g., sparse NMR contact maps. By themselves they provide insufficient information for structure generation and thus have to be complemented. By integrating NMR-derived distance restraints into ensemble MD simulations, Dedmon et al. have shown that the native state of the intrinsically disordered protein *α*-synuclein, which plays a key role in the pathogenesis of Parkinson’s, is composed of a more compact ensemble of conformations than would be expected for a random coil [26]. Coevolution analysis methods such as direct coupling analysis (DCA) [27] infer contact information from large multiple sequence alignments. DCA identifies coevolving residue pairs, which can be interpreted as spatially adjacent. This information was successfully used for structure prediction [28] even in large-scale studies of proteins [29] or for RNA [30]. DCA-derived contacts have already been combined with structure-based models to uncover conformational diversity for medium to large proteins, including hidden functional configurations and intermediate states [31]. However, it often is uncertain how error-prone available contact information actually is. NMR assignments can be wrong or DCA can contain false-positive contacts. For this purpose, we perform an extensive study to investigate the influence of native (“correct”) and non-native (“wrong”) contact information with regards to structure determination. To overcome kinetic entrapment due to the multiple-minima problem during a simulation, we use REMD as an enhanced sampling technique [15, 32–34].

Adding a contact-based bias to the energy function effectively guides the search towards the target structure by narrowing the conformational space to be sampled. By combining both contact information and REMD simulations, we drastically enrich native and native-like conformations in the simulated ensemble of a single run. To systematically study and test our method’s performance, we conduct REMD simulations of two small proteins with known native structures, starting from an unfolded state. We test different scenarios (see Table 1) by varying the bias quality, i.e. the true-positive rate (TPR), and the total number of randomly selected contact pairs. As the study is performed to assess the influence of both native and non-native contacts, we apply equal force coefficients *k* (see Eq (6)) to all used contact pairs. We analyze the data for each test case with simulated times of 250 ns, especially for the lowest-temperature replica. By comparing the test cases to a reference simulation not including any contact information, we can estimate the total number of required restraints and the bias strength. Furthermore, we investigate to what extent contact-guided REMD increases the chance of obtaining a native fold compared to normal MD. The study shows that our method yields high-quality results for both tested proteins as long as the bias has a TPR of approximately 75% or more. We find that such an energetic bias, even if containing false-positive contacts to a certain extent, greatly enhances the refinement process during REMD and improves the chance of finding the native state in a single run. It is possible to further include experimental information from other sources into such simulations and use them as a hybrid tool for joint data interpretation.

**Table 1:** Variation of bias quality in method performance study using REMD simulations.

TPR (%)	ref	100	100	100	100	100	75	75	75	75	50	50	50	50
# CP	0	6	12	24	36	48	12	24	36	48	12	24	36	48
# native	0	6	12	24	36	48	9	18	27	36	6	12	18	24
# non-native	0	0	0	0	0	0	3	6	9	12	6	12	18	24

Overview of the 14 REMD scenarios investigated in the performance study for both test proteins. Listed are the true-positive rate (TPR) of used contact pairs (CP) in percent, number of restraining CP used, number of native contacts, and number of non-native contacts. A visualization of the used contacts can be looked up in S1 Fig to S6 Fig in S1 Appendix.

## Materials and methods

### Molecular dynamics

Computer simulations are often used to complement or interpret results of real experiments. Molecular dynamics (MD) is such an *in silico* approach to study the movements of atoms or biomolecules. The interactions between all atoms are calculated by applying a force field, e.g. AMBER [35], CHARMM [36], GROMOS [37], or OPLS [38], to the system and solving Newton’s equations of motions. Depending on the goal and type of simulation, some force fields are better suited than others due to different approaches to modeling atomic interactions. Comparisons between the force fields and their performance can be found in many studies [39]. Typical time steps of a simulation are in the order of 1 to 2 fs. Using MD, it is then possible to observe details of molecular mechanisms such as protein folding or ligand binding by analyzing the simulated trajectory.

### Replica exchange

Replica-exchange molecular dynamics (REMD), also referred to as parallel tempering, is an enhanced sampling technique for MD [32–34]. This efficient method is commonly applied to overcome kinetic entrapment resulting from the multiple-minima problem during MD simulations. REMD simulates *N* non-interacting copies (“replicas”) of a system at different temperatures *T*_*i*_. Each replica corresponds to one of *N* MD simulations performed simultaneously. After a fixed time *dt*, the atom positions and momenta of replicas can be exchanged. The exchange probability is given by the Metropolis criterion [32]

$$\begin{array}{c} w_{X_{i}\to X_{j}}=\text{min}\left(1,e^{-\Delta }\right), \end{array}$$ (1)

$$\begin{array}{c} \Delta =\left(\beta_{j}-\beta_{i}\right)\left(E_{i}-E_{j}\right), \end{array}$$ (2)

where *X*_*i*_ denotes the state of replica *i*, $\beta_{i}^{-1}=k_{B}T$ the inverse temperature, and *E*_*i*_ the energy of state *X*_*i*_. Since exchange rates are significantly lower for large temperature differences, which can be seen from Δ in Eq (2), it is sufficient to only exchange adjacent replicas.

The intention of REMD is to enhance sampling of both high and low energy states, and the temperature range has to be chosen accordingly. Replicas at the highest occurring temperatures should have sufficient energy to overcome potential barriers. Meanwhile, low-temperature replicas are supposed to explore conformations close to local minima. In combination, REMD increases the chance of finding the global energy minimum and thus the native state of a protein. To achieve a random walk it is mandatory to aim for constant exchange rates across all replicas and to make sure that they are shuffled sufficiently.

### Replica-exchange temperature generator

In REMD simulations, every replica resembles the dynamics of a canonical ensemble, where the probability distribution of each microstate follows the Boltzmann distribution *e*^−*βE*^. Since exchange rates are proportional to the energy difference of two adjacent replicas (see Eq (2)), an exponential temperature distribution is needed to guarantee a random walk in conformation space [23, 32, 40]. This distribution is *a priori* unknown as it depends on the protein size and number of solvent molecules. However, an initial temperature distribution can be estimated [41]. A simple temperature generator is given by

$$T_{i}=T_{0}\cdot e^{k}.$$ (3)

*T*_*i*_ is the temperature of replica *i*, while *k* refers to the growth parameter which has to be modified based on the system size. To obtain more consistent exchange rates during the simulation across all replicas, we slightly modify the generator according to

$$T_{i}=T_{i-1}+a_{i}\cdot \Delta ,$$ (4)

$$\Delta =T_{0}\cdot (e^{ki}-e^{k(i-1)}).$$ (5)

Eq (4) recursively describes the temperature of replica *i*. Δ denotes the temperature difference of two adjacent replicas as specified in Eq (5), while *a*_*i*_ is a step-size modifying coefficient. With *a*_*i*_ = 1 ∀*i*, the generator will produce the same temperature distribution as given by Eq (3). To keep the exchange rates almost constant over the whole simulated temperature range, we increase *a*_*i*_ every ten replica by 4%. Used parameters and resulting temperature distributions for both Trp-Cage and VHP can be found in S2 Appendix.

### Sigmoid potential

In order to guide protein folding, we implement an attractive potential to increase the bias towards native-like conformations. Technically, we apply the force only between the C_*α*_ atoms of selected contact pairs via tabulated bonded interactions [42] by modifying the topology file. The potential is given by

$$\begin{array}{cc} V(r) & =k\;\sigma (r), \end{array}$$ (6)

with the force coefficient *k* and the sigmoid function

$$\begin{array}{cc} \sigma (r) & =\frac{A}{1+e^{-\alpha (r-r_{0})}}. \end{array}$$ (7)

**Fig 1:**
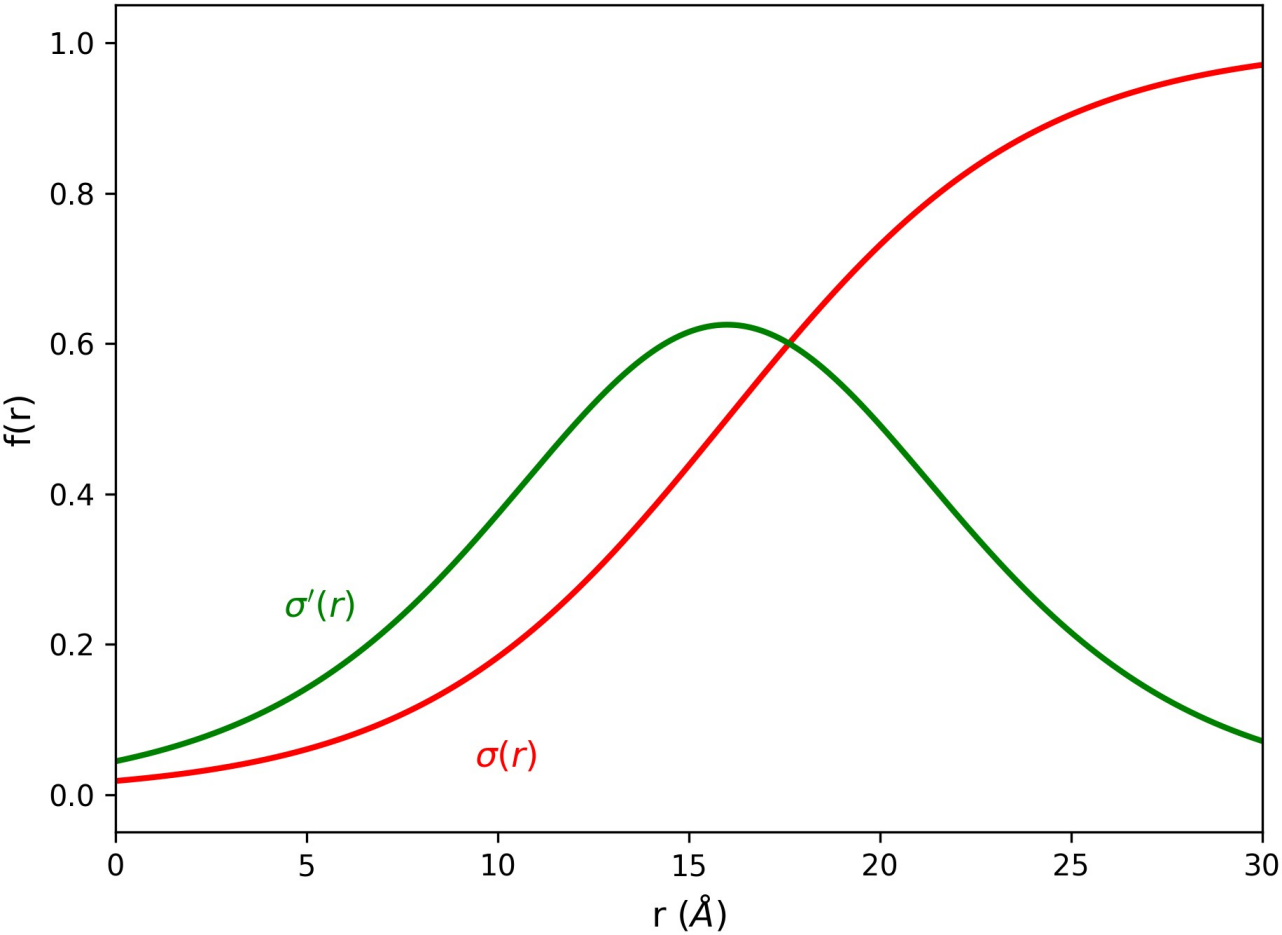
Sigmoid function of bias potential *V*(*r*). The used sigmoid function *σ*(*r*) with parameters *A* = 1, *α* = 2.5 nm^−1^, and *r*_0_ = 1.6 nm is represented by the red curve. The derivative *σ*′(*r*) is shown in green.

*A* corresponds to the sigmoid function’s limit, whereas *r* and *r*_0_ are the atom distance and equilibrium distance, respectively. *r*_0_ determines the position of the inflection point of the sigmoid function and thus the local maximum of the sigmoid function’s derivative. The parameter *α* affects the S-shape of the function, i.e. how fast the transition from low values to high values takes place. Fig 1 displays the used sigmoid function *σ*(*r*) in red and its derivative *σ*′(*r*) in green. For the sake of simplicity, we set *A* = 1 so that the force only depends on the force coefficient *k* = 10 kJ mol^-1^ (approximately 4*k*_B_ *T* at 300 K). *k* is chosen in such a way that the sigmoid potential at the native-defining distance *r*_nc_ = 0.6 nm (see Eq (8)) is in the order of a typical hydrogen bond strength [43]. *α* and *r*_0_ are set to 2.5 nm^−1^ and 1.6 nm, respectively, yielding a smooth transition of the potential while limiting its effective range locally. With this choice of *r*_0_, the potential affects biased contact pairs only for inter-contact distances *r*_*ij*_ ⪅ 3.2 nm. The greatest force appears at 1.6 nm, corresponding to two to three times of *r*_nc_. The system feels a drive towards its native state only if it already resembles that state to at least some extent. Otherwise, the potential has no effect at all and derived forces vanish as a result of its S-shape. With interactions above 3.2 nm virtually being neglected, the negative influence of improper contact information such as false-positive contacts from DCA can be limited [27]. Applying this potential to native contact pairs in REMD, the enhanced sampling of protein conformations can be utilized while simultaneously improving local refinement of the affected protein segments. Technically, the sigmoid potential can be used for arbitrarily large proteins because the contact bias only acts within the mentioned local region. The local interaction range can be changed by modifying the parameters *α* and *r*_0_. However, they should be kept rather small to prevent unphysical effects on a more global scale such as premature compaction of a structure. This potential can also be applied to multi-chain systems, in which case the pdb2gmx -merge all command of GROMACS is required to handle the multiple chains as one molecule type in a single topology file.

### Native contact enrichment

One key aspect of this study is to measure the influence and correlation of native and non-native contacts in REMD. For this purpose, we explicitly use randomly selected contact pairs from the known structures of the test systems. To quantify the TPR of chosen contact pairs, we define native contacts to fulfill the conditions

$$r_{ij}=|r_{i}-r_{j}|\leq 6Å\equiv r_{\text{nc}},$$ (8)

Δij=|i-j|≥4. (9)

While Eq (8) defines a maximum cutoff distance *r*_*ij*_ of 6 Å between C_*α*_ atoms of two residues *i* and *j*, Eq (9) excludes residue pairs very close to each other in the sequence which would appear on the main diagonal of a contact map. Based on these definitions, we create two separate lists with native and non-native contacts, respectively. More precisely, direct neighbors of native contacts within the contact map are omitted from the non-native list. For example, if residue pair (*i*, *j*) is native, then all nine combinations (*i*′, *j*′) with *i*′ ∈ {*i* − 1, *i*, *i* + 1} and *j*′ ∈ {*j* − 1, *j*, *j* + 1} are excluded. Lastly, we randomly select contact pairs from each list to construct different scenarios at fixed TPRs for the method performance study. Contact pairs (*i*, *j*) used as restraints can be looked up in the corresponding contact maps (cf. S1 Fig to S6 Fig in S1 Appendix).

### Global distance test

In the trajectory analyses, backbone root-mean-square deviation (RMSD) after structure alignment is considered to initially evaluate the method’s performance. In the context of protein structure determination, however, RMSD is a suboptimal measure of structural similarity as it strongly correlates with the largest displacement between mobile and target structure. This means if the mobile structure globally fits the target to a large extent and only one small segment is misaligned locally, the RMSD becomes disproportionately large. This problem is solved for the so-called Global Distance Test (GDT) [44–46], which is often used in CASP [12] to evaluate the accuracy of protein structures. Analogously to the RMSD, the mobile structure is first aligned to the target structure. Then, the displacement distance of each residual C_*α*_ atom is calculated and compared with various cutoff thresholds to estimate how similar the two structures are. In a last step, percentages of residues with displacements below a considered threshold are used to calculate score values. The two most common scores are the total score (TS),

$$\begin{array}{c} GDT_{\text{TS}}=\frac{1}{4}\left(P_{1}+P_{2}+P_{4}+P_{8}\right), \end{array}$$ (10)

and the high-accuracy (HA) score,

$$\begin{array}{c} GDT_{\text{HA}}=\frac{1}{4}\left(P_{0.5}+P_{1}+P_{2}+P_{4}\right). \end{array}$$ (11)

Here, variables *P*_*x*_ denote the percentage of residues with displacements below a distance cutoff of *x* Å. Note that both scores range between 0 and 100 and their interpretation is based on the “fit resolution” set by the applied cutoff distances. A protein model is considered topologically accurate for *GDT*_TS_ scores above 50 [12].

### Setup of REMD and MD simulations

All simulations were performed in GROMACS 2016.3 [42, 47]. We used the AMBER99SB-ILDN force field [35] and TIP3P explicit-solvent model [48]. Starting from the pdb structure, the protein was equilibrated in short NVP and NPT runs for 200 ps each and then unfolded in a normal MD simulation at a high temperature *T* = 500 K. We manually selected an unfolded state with high RMSD and minimal amount of remaining secondary structure as initial structure for the simulations. A REMD temperature generator based on Eqs (4) and (5) yielded the temperature distribution of *N* replicas (cf. S2 Appendix). After verifying sufficient exchange rates in short REMD simulations (every 1000 steps, rate approximately 16%), the sigmoid potential in Eq (6) was provided as a look-up table. Each REMD simulation comprised a simulated time of 250 ns with a time step of 2 fs and 60 and 100 replicas for Trp-Cage and Villin Headpiece, respectively. Restraints were added via tabulated bonded interactions [42] to the topology file. To compare the REMD results with MD simulations, we additionally performed two normal MD simulations (500 ns simulated time, with and without restraints) at *T*_0_ for each tested protein, starting from the same unfolded state as their REMD counterparts. S3 and S4 Appendices show the used mdp settings for REMD and MD simulations, respectively.

All production runs were performed on the ForHLR II computer cluster. We used thin nodes consisting of two Deca-Core Intel Xeon E5-2660 v3 processors (Haswell) with a base clock rate of 2.6 GHz (max. turbo-clock rate 3.3 GHz), 64 GB main memory, and 480 GB local SSD storage.

## Results and discussion

### Test systems

**Fig 2:**
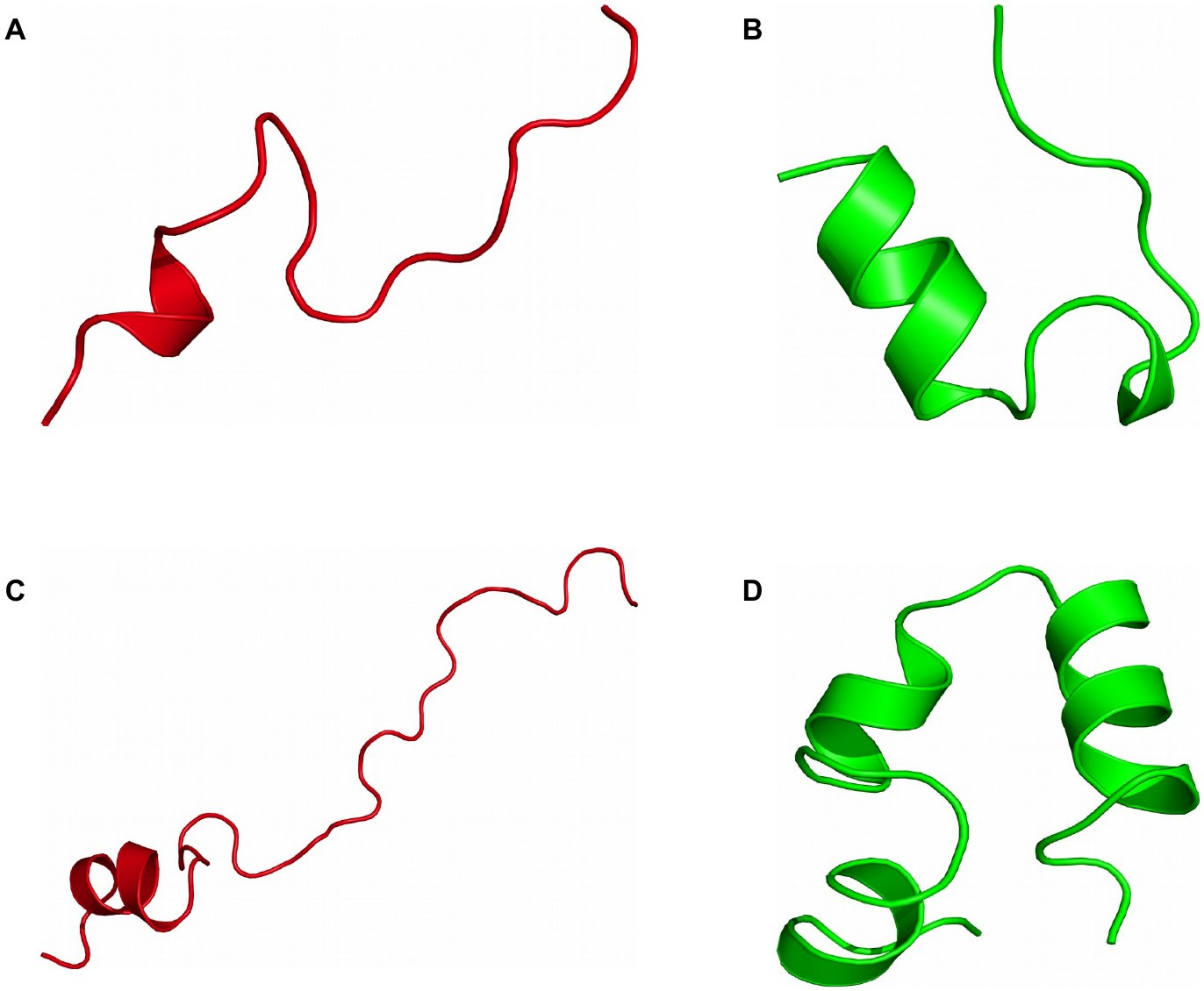
Initial and target conformations of considered test systems. Based on their high RMSDs with respect to the target state and a minimal amount of remaining secondary structure, initial conformations were manually selected from short unfolding simulations using MD at *T* = 500 K. Initial (A) and target (B) conformation of Trp-Cage have an RMSD of 8.8 Å with respect to each other. For VHP, the initial conformation (C) has an RMSD of 16.2 Å with respect to the target conformation (D).

We considered two well-known proteins for our systematic study of contact-guided REMD simulations. The first candidate is the 20-residue miniprotein Trp-Cage (PDB: 1l2y [49]). Its tertiary structure consists of an *α*-helix followed by a turn and a 3/10-helix. Trp-Cage was specifically designed as a fast folder and reaches folding times of approximately 4 *μ*s [50]. Its folding temperature is in the range of 311 to 317 K [51]. The second test system is Villin Headpiece (VHP, PDB: 1vii [52]). It has a sequence length of 35 residues and forms a three-helix structure. Similar to Trp-Cage, VHP can also achieve folding times in the order of *μ*s [53, 54] and has a folding temperature of approximately 339 to 342 K [55]. Fig 2 illustrates initial and target conformations of both proteins.

### Trp-Cage

**Fig 3:**
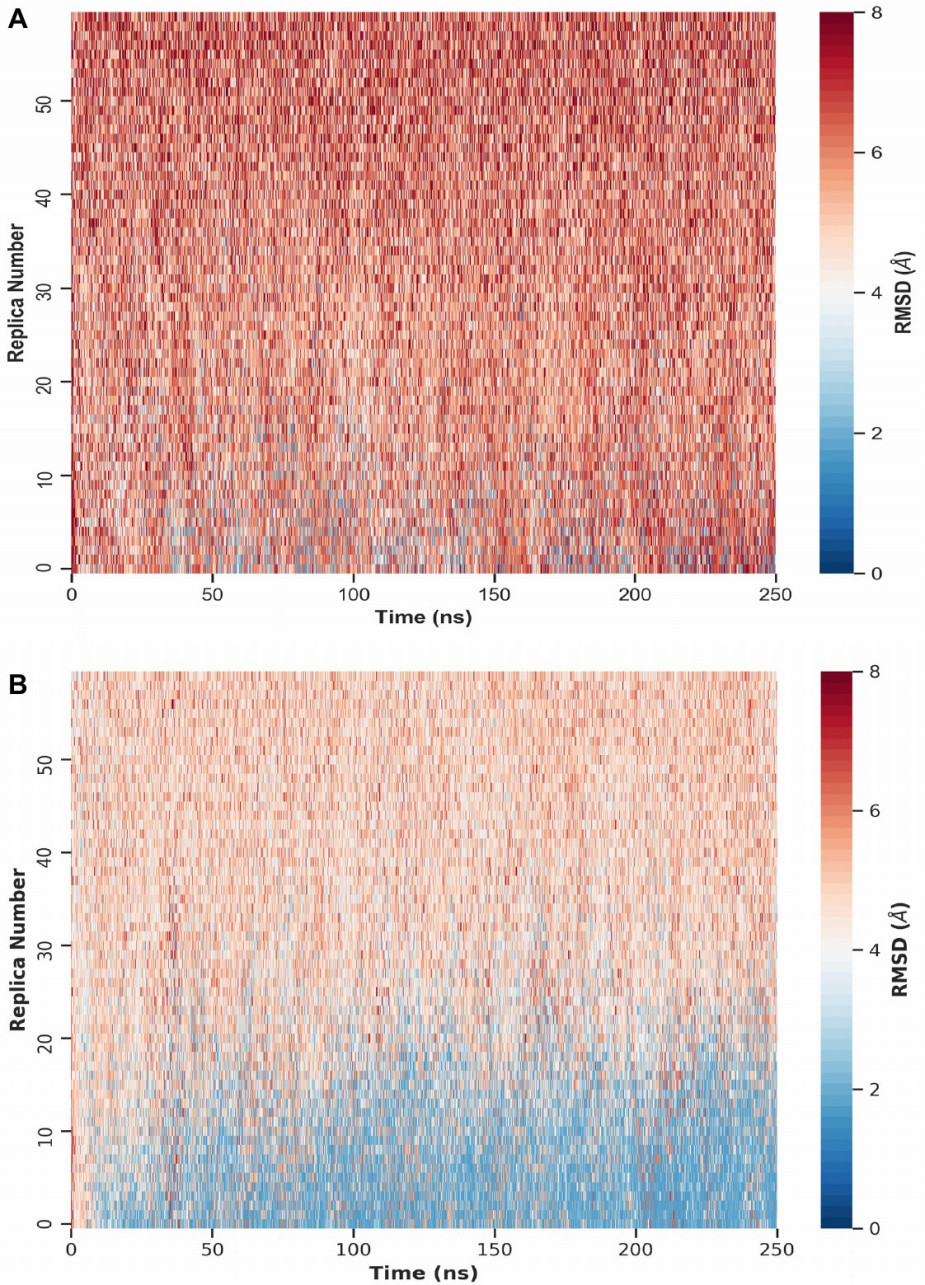
RMSD overview of Trp-Cage REMD simulations. Heatmaps display the backbone RMSD across all replicas. (A) Reference REMD simulation without any bias. (B) REMD simulation with bias of 12 native contacts at 100% TPR.

**Fig 4:**
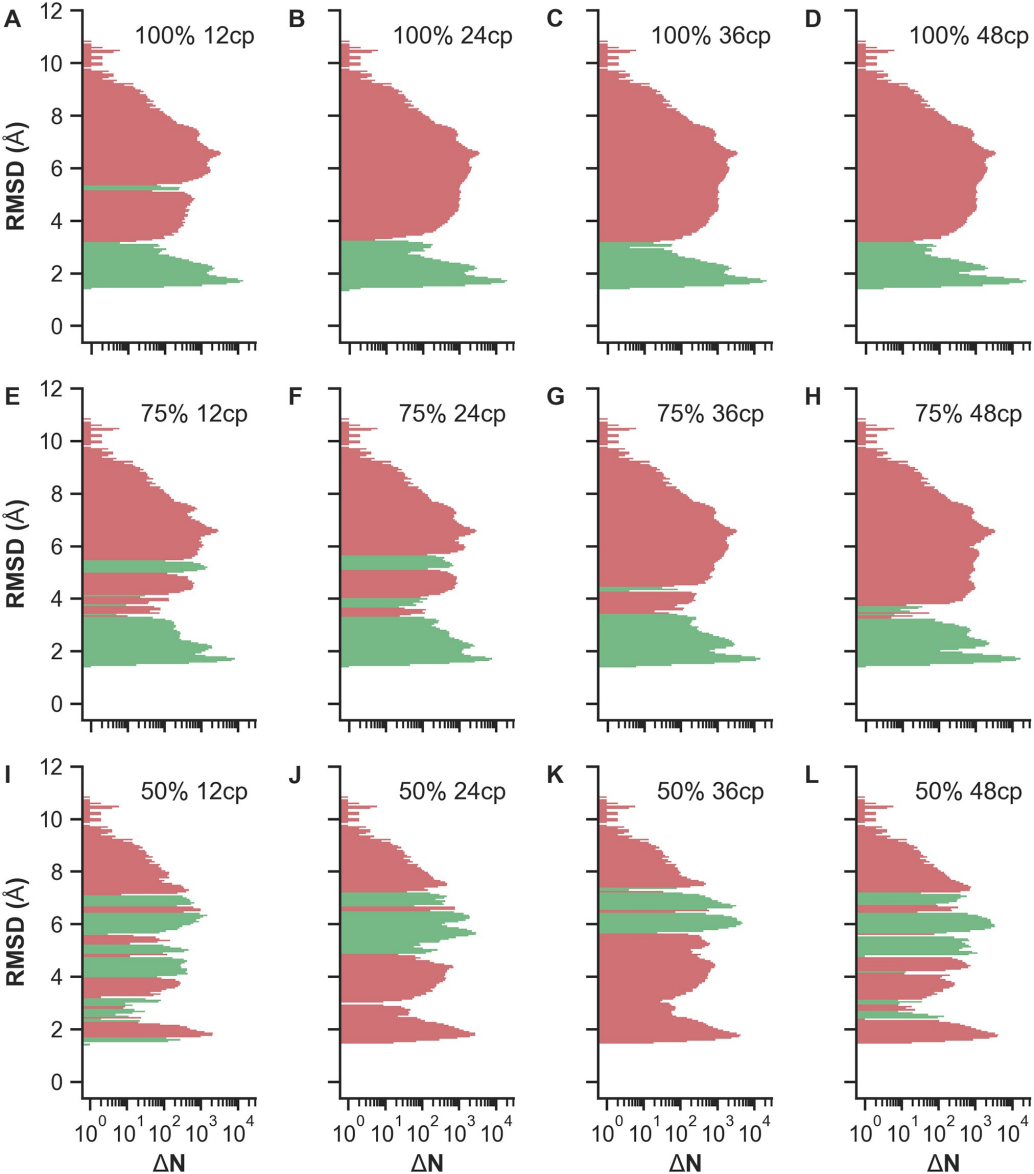
Δ*N* histograms of Trp-Cage REMD simulations. Histograms show the enrichment and depletion of conformations with a particular backbone RMSD at *T*_0_ = 300 K as compared to the reference. Histogram bins are defined by the RMSD axis, while the logarithmic Δ*N* axis illustrates the count difference between the tested REMD scenarios and the REMD reference simulation with Δ*N* = *N*_scenario_ − *N*_ref_. Positive (negative) values corresponding to enrichment (depletion) are shown in green (red). (A-D) Simulations with 100% TPR and 12, 24, 36, 48 contact pairs. (E-H) Simulations with 75% TPR and 12, 24, 36, 48 contact pairs. (I-L) Simulations with 50% TPR and 12, 24, 36, 48 contact pairs.

We performed REMD simulations of Trp-Cage for a total of 60 replicas ranging from *T*_0_ = 300 K to *T*_59_ = 625 K, each yielding a trajectory of 250 ns simulated time. The wide temperature range was deliberately chosen to encourage large-scale conformational transitions in each REMD turnaround before cooling down to the lowest temperatures. To better compare the different scenarios listed in Table 1, all REMD simulations initiate from the same unfolded conformation with a backbone RMSD of 8.8 Å with respect to the target state (cf. Fig 2A and 2B). A sigmoid potential as given in Eq (6) is assigned to each implemented restraint between a C_*α*_-C_*α*_ pair with the same coupling strength *k* = 10 kJ mol^−1^. The resulting force is distance-dependent and acts only locally up to a C_*α*_-C_*α*_ threshold of approximately 3.2 nm, with the highest force at 1.6 nm here. Generally, this sigmoid potential can be used for proteins of any size as it will affect the selected contact pairs only in the mentioned local region. In this way, unphysical compaction of structures on a global scale may be prevented while improving accuracy of structural motifs locally. The short-range limitation of the potential also minimizes the influence of erroneously used non-native contact pairs. Intra-contact interactions above 3.2 nm distances are virtually neglected due to the asymptotic behavior of the sigmoid potential. First, we investigated whether the coupling strength of the restraints is sufficiently strong to guide the protein towards native-like structures during REMD. For this purpose, we determined the time-dependent backbone RMSD with respect to the native structure for all replicas in each test case. To obtain a general overview and qualitatively compare the RMSD statistics across the scenarios, color-coded RMSD values are displayed as heatmaps. Fig 3A and 3B exemplarily show RMSD heatmaps of the reference simulation and the simulation with 12 native contacts, respectively. The reference case mostly features RMSDs greater than 4 Å (red), with the majority being about 6 Å and a few occurrences of low RMSDs in lower-temperature replicas. As evident from Fig 3B, a bias potential with purely native contact restraints strongly improves RMSDs of lower-temperature replicas. In contrast to the reference simulation, RMSDs mostly decrease below 4 Å (blue) for replicas with temperatures below *T*_9_ ≈ 332 K and *T*_17_ ≈ 366 K after only 10 and 100 ns of simulated time, respectively. Heatmaps of the other ideal cases, i.e. with contact pairs at 100% TPR, are shown in S7 Fig to S9 Fig in S1 Appendix. Compared to the unbiased reference simulation, the blue region grows with enhanced “correct” bias towards the native conformation. The number of implemented contact restraints appears to be correlated with the enrichment of native-like conformations, and sufficiently good folds may even be obtained from higher-temperature replicas. However, since native conformations are considered the lowest-energy states, it is most important to compare the absolute RMSDs of low-temperature replicas as well as their improvement or deterioration between the different scenarios. The greatest incremental improvement for lower-temperature replicas is observed for the transition from 6 to 12 native contacts (cf. S7 Fig and S8 Fig in S1 Appendix). Additional contacts only marginally improve the results as the low-temperature region is already sufficiently saturated with low-RMSD conformations. However, with any sort of contact information typically being error-prone, it is realistically impossible to apply a perfect bias corresponding to a TPR of 100%. To estimate the bias quality threshold required to easily obtain native-like structures within contact-guided REMD, mixed scenarios containing both true- and false-positive contacts were performed. Heatmaps of simulations using restraints at 75% TPR are shown in S10 Fig and S11 Fig in S1 Appendix, whereas the simulations with highly error-prone restraints at only 50% TPR are depicted in S12 Fig and S13 Fig in S1 Appendix. A qualitative comparison of 100% and 75% scenarios shows the same overall tendencies, i.e. the blue region corresponding to conformations with RMSDs below 4 Å expands towards higher temperatures and saturates faster with increasing number of used contact pairs. As expected, this effect is significantly stronger for scenarios implementing only native contacts. For example, REMD simulations with 100% TPR and 12 or 24 contacts are very similar to the cases with 75% TPR and 36 (27 native, 9 non-native) or 48 (36 native, 12 non-native) contacts, respectively. Taking the RMSDs of all replicas into account, non-native contact restraints appear to strongly deteriorate the overall results. This becomes particularly obvious when inspecting scenarios with only 50% TPR. Here, RMSD heatmaps contain mainly values between 4 and 6 Å across all replicas and are on a par to or slightly worse than the unrestrained reference REMD simulation. Next, we primarily analyzed RMSDs of the lowest-temperature replica at *T*_0_ = 300 K to see which conformations effectively become enriched. This allows us to estimate the limitations of contact-guided REMD and compare the results at the most relevant temperature more quantitatively. A so-called Δ*N* histogram displays the count difference of observed backbone RMSDs of each tested scenario with respect to the reference simulation. For Trp-Cage, such histograms are presented in Fig 4, summarizing all performed REMD simulations. Simulations with purely native contacts (Fig 4A–4D) show a strong enrichment of conformations with RMSDs between 1.6 and 3.0 Å as indicated by the green bins. Counts of conformations with RMSDs above 3.0 Å reduced accordingly. As evident from Fig 4A–4D, the net gain of native-like folds does not improve when the bias exceeds 12 restraints, corresponding to approximately *L*/2 contact pairs with the sequence length *L*. Test cases with mixed contacts at 75% TPR (Fig 4E–4H) show a similar behavior. Additionally, the scenarios with 12 and 24 mixed contacts also enrich conformations with RMSDs around 5.0 Å, apparently biased by the first few non-native contacts. Scenarios with a TPR of only 50% (Fig 4I–4L), where low-RMSD conformations become depleted and not enriched, show far worse statistics compared to the reference simulation. On this account, they turn out to be inappropriate for the intended purpose of structure determination within contact-guided REMD. A theoretical edge case occurs for equally contributing native and non-native contacts at 50% TPR. While half of the contacts (true-positives) would lower the global minimum, the other half (false-positives) would either lower existing local minima or introduce new unphysical ones. As the global minimum remains global under these circumstances, obtaining the native state is still possible with the help of enhanced sampling in REMD. This edge case is, however, usually not met as the used contacts are not equally contributing due to the distance dependency. Furthermore, if the implemented restraints are clustered within the contact map, the local bias adds up and can be strong enough to entrap the protein in unfavorable conformations. To compensate such effects, the force coefficient *k* of the sigmoid potential (see Eq (6)) needs to be reduced when using large numbers of highly error-prone contact restraints with respect to the protein length.

The disadvantage of RMSD-based evaluation of structure quality is that local deviations between mobile and target structure from, e.g., poor modeling of loop regions in an otherwise reasonably fitting structure already result in a disproportionate increase. This is why we transition to the so-called Global Distance Test (GDT) which takes local misalignments better into account. GDT (also *GDT*_TS_ for “total score”) is a structural similarity measure for two different conformations of the same protein. It is computed as the largest set of amino-acid C_*α*_ atoms in the model structure lying within a defined cutoff distance of their position in the reference structure after superposition. Thus, the higher GDT, the more a given model conforms to the reference structure. The GDT algorithm originally calculates the fraction of C_*α*_ atoms lying within the radius of 20 consecutive distance cutoffs (0.5 Å, 1.0 Å, 1.5 Å,…,10.0 Å) from their individual positions in the target structure. This fraction generally increases with the cutoff, where a plateau usually indicates significant divergence, meaning that no additional atoms are included in any cutoff of a reasonable distance. The more rigorous high-accuracy version *GDT*_HA_ uses smaller cutoff distances, usually half the size of *GDT*_TS_. The distributions of the two scores *GDT*_TS_ and *GDT*_HA_ provide an additional perspective to the estimation of the bias quality necessary for effective integration of contact information into REMD. Table 2 gives an overview of occurring percentiles of *GDT*_TS_ and *GDT*_HA_ scores for all performed REMD and MD simulations of Trp-Cage at *T* = 300 K. All corresponding histograms are displayed in S30 Fig to S43 Fig in S1 Appendix. For each score variant, shaded table cells indicate improved percentiles *P*_x_ compared to the REMD reference *P*_x,ref_, i.e. cells with

$$\begin{array}{c} P_{x}\geq P_{x,\text{ref}}. \end{array}$$ (12)

Additionally, a bold font is applied to values which satisfy

$$\begin{array}{c} P_{x}\geq P_{x,\text{ref}}+w\cdot \left(P_{100,\text{ref}}-P_{x,\text{ref}}\right) \end{array}$$ (13)

to highlight a significant improvement. In Eq (13), each *P*_x_ is compared to a percentile-specific threshold depending only on corresponding reference values. The threshold is defined as the sum of the percentile itself and a weighted difference of this percentile to the highest observed value. The difference indicates the practically possible improvement in relation to the reference. So as to reflect significant improvement, we set the coefficient *w* to 50%. In scenarios with TPRs of at least 75%, the TS distribution is clearly shifted from 53.75 to scores above 96 already at the 80th percentile. This means that 20% of the simulated structures in the trajectory already adopted conformations which are almost identical to the native conformation. HA scores similarly show a significant improvement. It is particularly remarkable that the reference simulation yielded an exceptional HA score of 98.75. The comparison of 50% TPR simulations to the reference shows that highly error-prone contact bias has a very negative influence and is insufficient to effectively increase the frequency of native-like structures in contact-guided REMD.

**Table 2:** Total Score (TS) and High Accuracy (HA) percentiles of Trp-Cage simulations.

Method	TPR (%)	# CP	$P_{\text{TS}}^{80}$	$P_{\text{TS}}^{85}$	$P_{\text{TS}}^{90}$	$P_{\text{TS}}^{95}$	$P_{\text{TS}}^{100}$	$P_{\text{HA}}^{80}$	$P_{\text{HA}}^{85}$	$P_{\text{HA}}^{90}$	$P_{\text{HA}}^{95}$	$P_{\text{HA}}^{100}$
REMD	ref	0	53.75	88.75	93.75	96.25	100.00	30.00	67.50	76.25	81.25	98.75
REMD	100	6	**96.25**	**96.25**	**97.50**	97.50	**100.00**	**80.00**	81.25	82.50	85.00	96.25
REMD	100	12	**96.25**	**97.50**	**97.50**	**98.75**	**100.00**	**81.25**	82.50	83.75	86.25	97.50
REMD	100	24	**97.50**	**97.50**	**97.50**	**98.75**	**100.00**	**82.50**	**83.75**	85.00	86.25	97.50
REMD	100	36	**97.50**	**97.50**	**97.50**	**98.75**	**100.00**	**82.50**	**83.75**	85.00	86.25	97.50
REMD	100	48	**97.50**	**97.50**	**98.75**	**98.75**	**100.00**	**82.50**	**83.75**	85.00	86.25	97.50
REMD	75	12	**95.00**	**96.25**	**97.50**	97.50	**100.00**	**78.75**	80.00	82.50	85.00	**98.75**
REMD	75	24	**95.00**	**96.25**	96.25	97.50	**100.00**	**77.50**	80.00	81.25	83.75	96.25
REMD	75	36	**96.25**	**96.25**	**97.50**	97.50	**100.00**	**80.00**	81.25	82.50	85.00	96.25
REMD	75	48	**96.25**	**96.25**	**97.50**	**98.75**	**100.00**	**80.00**	81.25	83.75	85.00	97.50
REMD	50	12	41.25	47.50	85.00	95.00	**100.00**	16.25	23.75	62.50	77.50	96.25
REMD	50	24	40.00	42.50	47.50	91.25	**100.00**	17.50	20.00	23.75	71.25	96.25
REMD	50	36	36.25	38.75	41.25	43.75	95.00	15.00	17.50	20.00	22.50	82.50
REMD	50	48	37.50	38.75	42.50	46.25	93.75	15.00	17.50	18.75	23.75	76.25
MD	ref	0	33.75	36.25	40.00	43.75	56.25	11.25	13.75	16.25	20.00	32.50
MD	100	12	53.75	55.00	56.25	57.50	68.75	28.75	30.00	31.25	33.75	45.00

Overview of observed Global Distance Test (GDT) percentiles. Statistics were taken from trajectories at *T* = 300 K over 250 ns for REMD and 500 ns for MD, respectively. Listed are the simulation method, the true-positive rate (TPR) in percent, used number of restraining contact pairs (CP), GDT total score percentiles (*P*_TS_), and GDT high accuracy percentiles (*P*_HA_). Values equal to or greater than the respective reference are shaded in gray. According to Eq (13) significantly greater values are bold.

**Fig 5:**
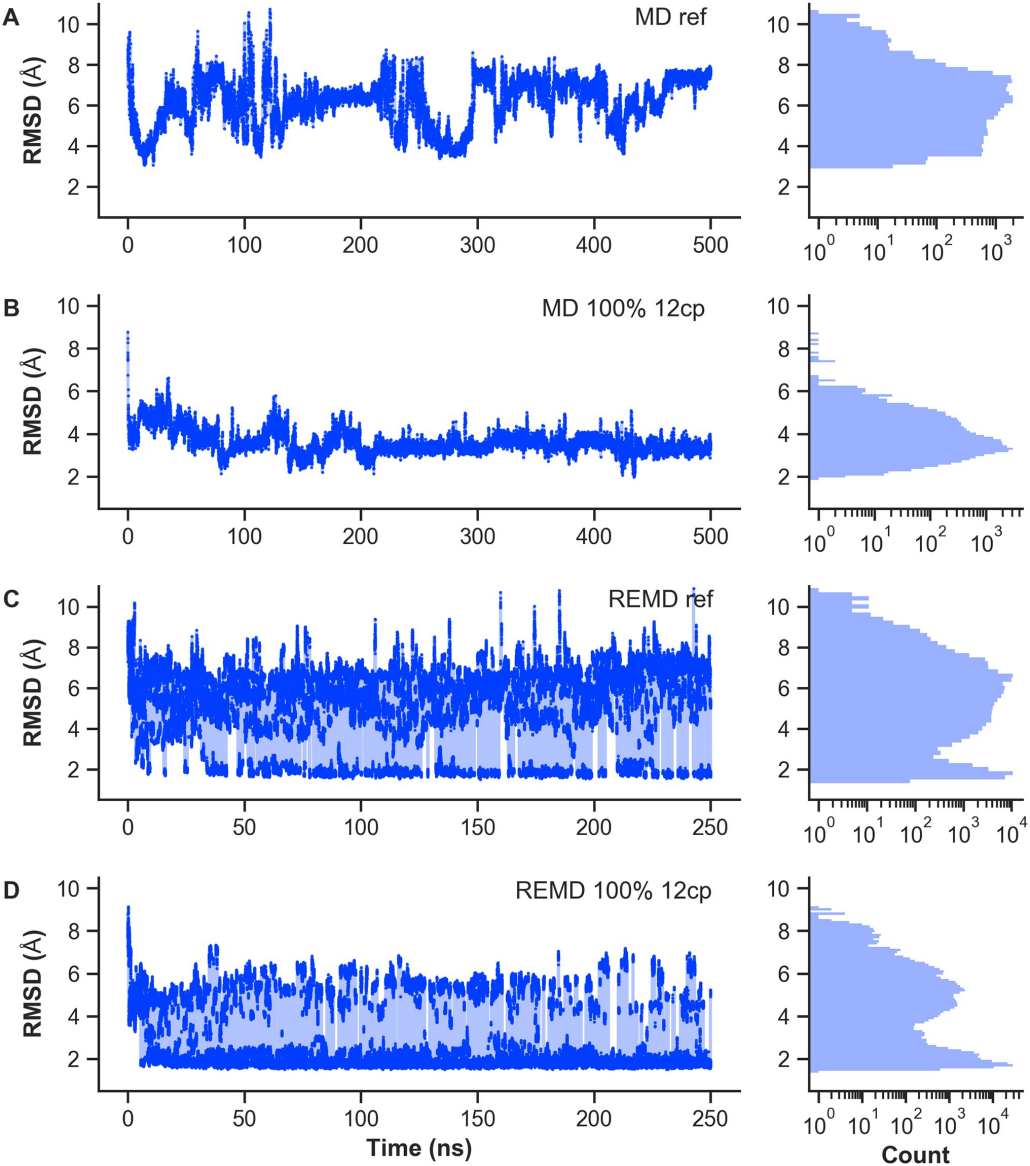
Comparison of Trp-Cage MD and REMD simulations. Figure shows the backbone RMSD time evolution at *T* = 300 K and the corresponding histogram with logarithmic count axis. Values were taken from 500 ns MD and 250 ns REMD trajectories, respectively. (A) MD reference simulation without additional bias. (B) MD simulation with 12 native contact restraints. (C) REMD reference simulation without additional bias. (D) REMD simulation with 12 native contact restraints.

Lastly, to justify the higher computational costs resulting from simulating multiple replicas in parallel, we conducted additional MD simulations without contact integration and with 12 native contacts as restraints. Fig 5 shows the RMSD evolution with simulated time at 300 K and the resulting histograms as compared to the corresponding REMD scenarios. With an initial RMSD of 8.8 Å from the native state, the reference MD simulation exhibits a completely random behavior as expected. Note that the MD trajectories comprise 500 ns instead of just 250 ns as for REMD. Observed conformations mainly show RMSDs between 4 and 8 Å. The best achieved values of approximately 3.0 to 3.5 Å are reached only a few times. With the majority of RMSDs around 3.5 Å and best values at 2 Å, the MD simulation with 12 native contact restraints yields significantly better statistics. The protein apparently became trapped in a certain configuration favored by the implemented contact bias and henceforth changes only marginally throughout the simulation. Switching to unrestrained REMD, we clearly observe a much broader variety of occurring RMSDs, ranging from approximately 1.7 Å to more than 11 Å. Although we see a high frequency of native-like conformations, the majority still has RMSDs in the range of 4 to 8 Å. Including contact restraints in the biased REMD simulation yields a drastic improvement compared to the unbiased case. Native-like conformations occur more frequently and now even form the majority of counts. In this light, we conclude that the increased computing demands can be justified based on the observed performances. Besides, even many long MD simulations are not guaranteed to ever reach the native fold due to kinetic entrapment while REMD does not encounter this problem. For completeness, additional RMSD curves and histograms of the lowest-temperature replica for Trp-cage REMD simulations can be looked up in S14 Fig to S17 Fig in S1 Appendix.

### Villin headpiece

**Fig 6:**
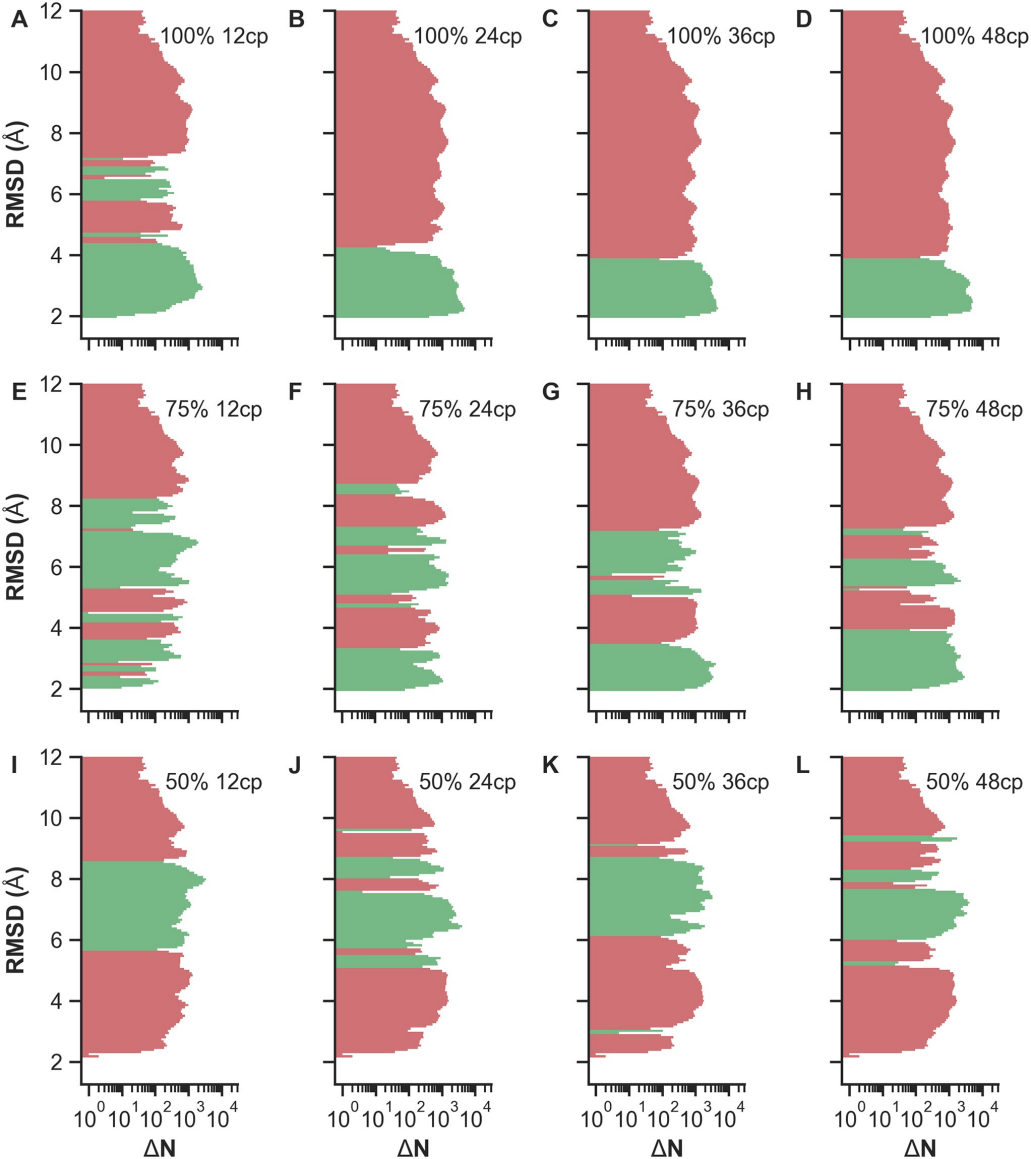
Δ*N* histograms of VHP REMD simulations. Histograms show the enrichment and depletion of conformations with a particular backbone RMSD at *T*_0_ = 300 K as compared to the reference. Histogram bins are defined by the RMSD axis, while the logarithmic Δ*N* axis illustrates the count difference between the tested REMD scenarios and the REMD reference simulation with Δ*N* = *N*_scenario_ − *N*_ref_. Positive (negative) values corresponding to enrichment (depletion) are shown in green (red). (A-D) Simulations with 100% TPR and 12, 24, 36, 48 contact pairs. (E-H) Simulations with 75% TPR and 12, 24, 36, 48 contact pairs. (I-L) Simulations with 50% TPR and 12, 24, 36, 48 contact pairs.

VHP REMD simulations were performed under the same conditions as for Trp-Cage. The selected initial conformation has a backbone RMSD of 16.2 Å compared to the target conformation (cf. Fig 2C and 2D). Due to the increased system size, 40 additional replicas were required to achieve nearly constant exchange rates across the considered temperature range. RMSD heatmaps (cf. S18 Fig to S24 Fig in S1 Appendix) show a similar behavior regarding the observed RMSD statistics when increasing the bias quality or number of used contact restraints. Compared to Trp-Cage, however, the improvements are less prominent. The reference shows very poor RMSD statistics throughout as seen in S18A Fig in S1 Appendix. A. The majority of the heatmap displays RMSDs well above 8 Å. The lower-temperature replicas mainly yielded RMSDs in the range of 4 to 6 Å. Despite the enhanced sampling of REMD we observe that the simulated time span of 250 ns is too short for VHP to be guided towards the native structure without additional bias. When the bias potential is added, we again observe improved RMSDs for the lower temperatures, reflected by a color transition from red to white to blue within the heatmap plots. In case of REMD with 100% TPR and 12 contact pairs (cf. S19A Fig in S1 Appendix), replicas with temperatures below *T*_19_ ≈ 340 K yielded many conformations with RMSDs around 4 Å (white). Increasing the number of used contacts to 24, RMSDs further reduced to values around 2.5 to 3.0 Å(blue). Besides, the greatest incremental improvement of all 100% TPR REMD simulations was observed for the transition from 12 to 24 native contacts. Scenarios with 36 and 48 native contacts are qualitatively as good as the case with 24 native contacts. Analogous to Trp-Cage, scenarios with 75% TPR also lead to a general improvement of RMSDs in lower-temperature replicas, while scenarios with only 50% TPR have the opposite effect as shown in S21 Fig to S24 Fig in S1 Appendix. Δ*N* histograms visualizing the enrichment and depletion of conformations with specific RMSD values at *T*_0_ = 300 K are summarized in Fig 6. Scenarios under perfect conditions, i.e. at 100% TPR, are illustrated in Fig 6A–6D. Large improvements are made up to 24 restraints, while scenarios with 36 and 48 restraints show almost identical results consistent with the qualitative comparison of the previously discussed heatmaps. Conformation counts with RMSDs above 4.0 Å were greatly reduced, while those with RMSDs between approximately 2.0 and 4.0 Å became enriched. Scenarios with mixed contacts at 75% TPR are displayed in Fig 6E–6H. Here, we still observe an enrichment of low-RMSD conformations but also a drastic increase of conformations with RMSDs around 5.0 to 8.0 Å. The enrichment of the higher-RMSD conformations is strongly influenced by the first three non-native contacts of the first 12 contact pairs (cf. cyan contacts in S5 Fig in S1 Appendix), which were randomly selected for this test case. Long-range contacts (*i*, *j*), which are far off the main diagonal in the contact map, can contribute a stronger bias compared to contacts with a small difference in their sequence numbers Δ_*ij*_ = |*i* − *j*| close to the main diagonal. This results from the choices of parameters in the sigmoid potential, determining its local field of action as well as the intra-contact distance with the greatest force. Since contacts close to the main diagonal are spatially close, it is likely that the attractive force is always below its possible maximum. Long-range contacts, however, can have distances even beyond the potential’s effective range and thus may experience the maximum possible force at some point during the simulation. This means that not all implemented contacts contribute equally and those with a certain displacement to the main diagonal can yield a much stronger bias. Nonetheless, all simulations with a TPR of 75% show a net gain of native-like conformations and therefore should be favored over the unbiased scenario. Scenarios with a TPR of 50% are displayed in Fig 6I–6L. Here, we observe that both low- and high-RMSD conformations appeared less frequently compared to the reference, whereas conformations between 5.5 and 8.5 Å became enriched. This once again shows that a low ratio of native to non-native contacts yields worse results than the unbiased case.

**Fig 7:**
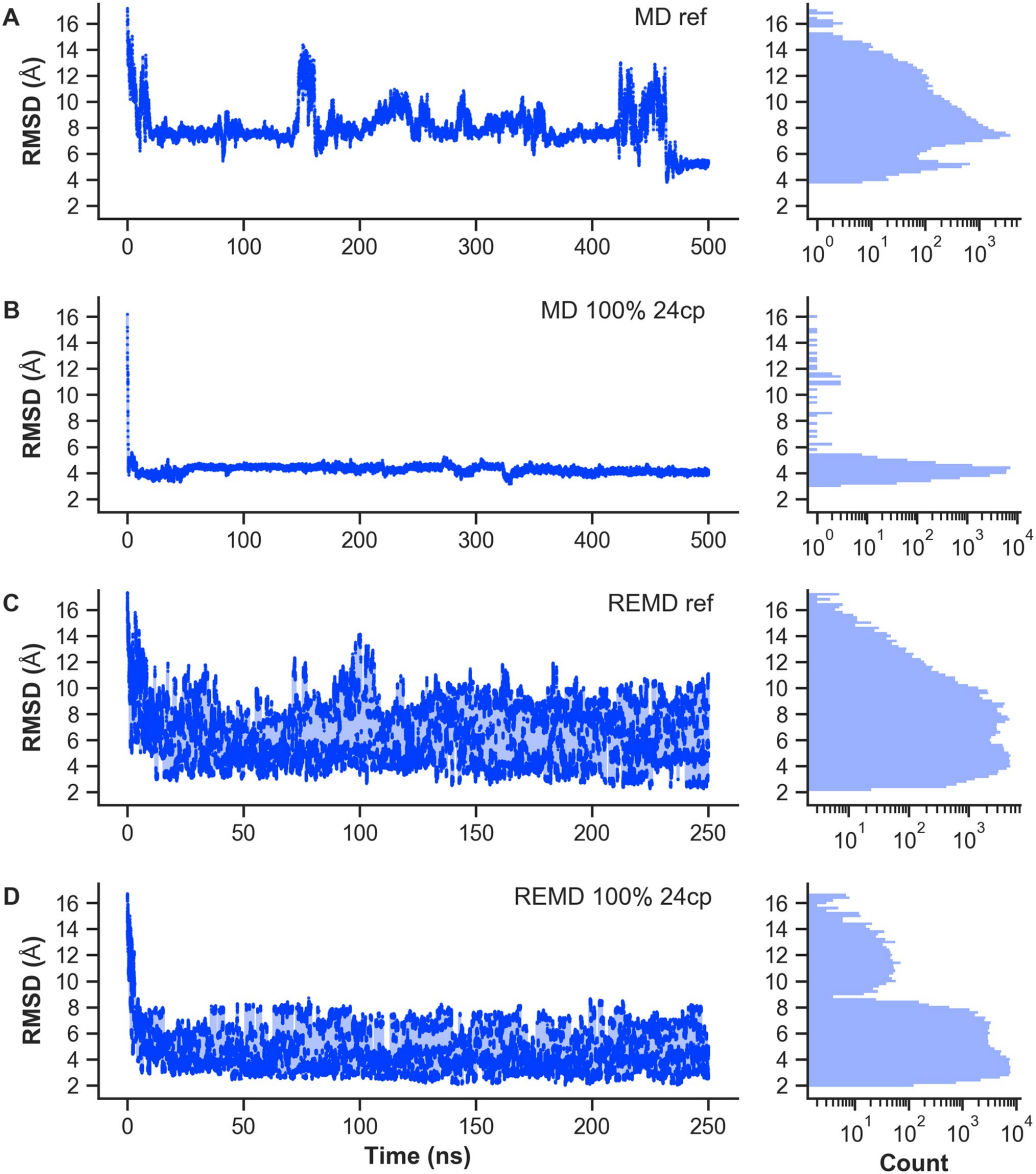
Comparison of VHP MD and REMD simulations. Figure shows the backbone RMSD time evolution at *T* = 300 K and the corresponding histogram with logarithmic count axis. Values were taken from 500 ns MD and 250 ns REMD trajectories, respectively. (A) MD reference simulation without additional bias. (B) MD simulation with 24 native contact restraints. (C) REMD reference simulation without additional bias. (D) REMD simulation with 12 native contact restraints.

Table 3 specifies the observed percentiles of *GDT*_TS_ and *GDT*_HA_ score distributions for VHP REMD and MD simulations at *T* = 300 K. The corresponding histograms are shown in S30 Fig to S43 Fig in S1 Appendix. We find that the REMD simulations benefited from restraints with a TPR of 75% or higher, resulting in significantly better GDT scores according to Eq (13). Here, ideal results in mixed scenarios with 75% TPR are observed for restraint numbers in the order of the protein length *L*. Analogous to previous observations during the RMSD-based discussion, simulations with only 50% TPR were worse compared to the reference scenario. We conclude that a bias of such poor quality is generally unsuited to achieve useful results within contact-guided REMD simulations. Fig 7 summarizes the comparison of REMD versus MD simulations, each with and without 24 native contact restraints. RMSD curves at 300 K as well as corresponding histograms are depicted. Starting from an unfolded state with an RMSD of 16.2 Å with respect to the native fold, the reference MD simulation shows a typical folding curve. The protein quickly collapses to conformations with RMSDs around 8 Å. During the simulation, the protein undergoes a few larger structural changes, even achieving conformations with an RMSD of around 4 Å after 460 ns. In the MD simulation with 24 restraining native contacts, the protein immediately transitions to conformations around 4 Å and continues to be trapped within this RMSD range. Best achieved RMSDs are as low as 3 Å. The reference REMD simulation displays a broad variety of RMSDs between 2 and 17 Å. Overall, a random behavior without clear tendencies towards certain conformations is observed. REMD with 24 native contacts yielded a strong depletion of conformations between 9 and 17 Å as compared to the reference REMD. Accordingly, conformations with RMSDs lower than approximately 8 Å appeared more frequently with a slight tendency towards lower values such as 3 to 4 Å. The best achieved RMSD during this simulation was 1.9 Å. Cf. S25 Fig to S28 Fig in S1 Appendix. for additional RMSD curves and histograms of VHP REMD simulations.

**Table 3:** Total Score (TS) and High Accuracy (HA) percentiles of VHP simulations.

Method	TPR (%)	# CP	$P_{\text{TS}}^{80}$	$P_{\text{TS}}^{85}$	$P_{\text{TS}}^{90}$	$P_{\text{TS}}^{95}$	$P_{\text{TS}}^{100}$	$P_{\text{HA}}^{80}$	$P_{\text{HA}}^{85}$	$P_{\text{HA}}^{90}$	$P_{\text{HA}}^{95}$	$P_{\text{HA}}^{100}$
REMD	ref	0	50.00	53.47	57.64	63.89	79.17	27.08	30.56	34.72	40.98	58.34
REMD	100	6	**66.67**	**68.75**	**71.53**	**75.00**	**87.50**	**43.06**	**45.14**	**47.92**	**51.39**	**68.06**
REMD	100	12	61.11	63.19	65.97	69.44	**86.11**	37.50	39.58	42.36	45.84	**65.28**
REMD	100	24	**71.53**	**73.61**	**75.00**	**77.08**	**88.89**	**47.92**	**49.30**	**51.39**	**53.47**	**68.75**
REMD	100	36	**71.53**	**73.61**	**75.00**	**77.08**	**88.89**	**47.92**	**50.00**	**51.39**	**54.16**	**70.83**
REMD	100	48	**72.22**	**73.61**	**75.00**	**77.08**	**87.50**	**48.61**	**50.00**	**51.39**	**53.47**	**68.06**
REMD	75	12	47.92	50.70	54.17	59.02	**87.50**	24.30	27.08	30.56	34.72	**65.97**
REMD	75	24	49.30	54.86	59.02	70.14	**84.72**	25.00	30.56	34.72	45.83	**64.58**
REMD	75	36	**68.06**	**71.53**	**74.31**	**77.08**	**88.89**	**43.75**	**47.22**	**50.00**	**53.47**	**70.83**
REMD	75	48	62.50	65.97	**69.44**	**73.61**	**85.42**	38.89	42.36	45.83	49.30	**63.89**
REMD	50	12	34.03	38.89	44.44	50.70	**79.17**	13.20	17.36	21.53	27.08	55.56
REMD	50	24	31.25	34.03	36.80	44.45	73.61	10.42	11.81	14.58	22.22	50.00
REMD	50	36	28.47	31.94	36.11	40.28	70.83	9.03	11.11	14.58	18.06	49.30
REMD	50	48	28.47	30.56	34.03	36.81	59.72	9.03	9.72	12.50	15.28	36.11
MD	ref	0	25.70	27.08	28.47	35.42	50.00	9.03	9.72	11.11	13.19	26.39
MD	100	24	41.66	42.36	43.06	44.44	57.64	17.36	18.06	18.75	20.14	32.64

Overview of observed Global Distance Test (GDT) percentiles. Statistics were taken from trajectories at *T* = 300 K over 250 ns for REMD and 500 ns for MD, respectively. Listed are the simulation method, the true-positive rate (TPR) in percent, used number of restraining contact pairs (CP), GDT total score percentiles (*P*_TS_), and GDT high accuracy percentiles (*P*_HA_). Values equal to or greater than the reference values are shaded in gray. According to Eq (13) significantly greater values are bold.

**Fig 8:**
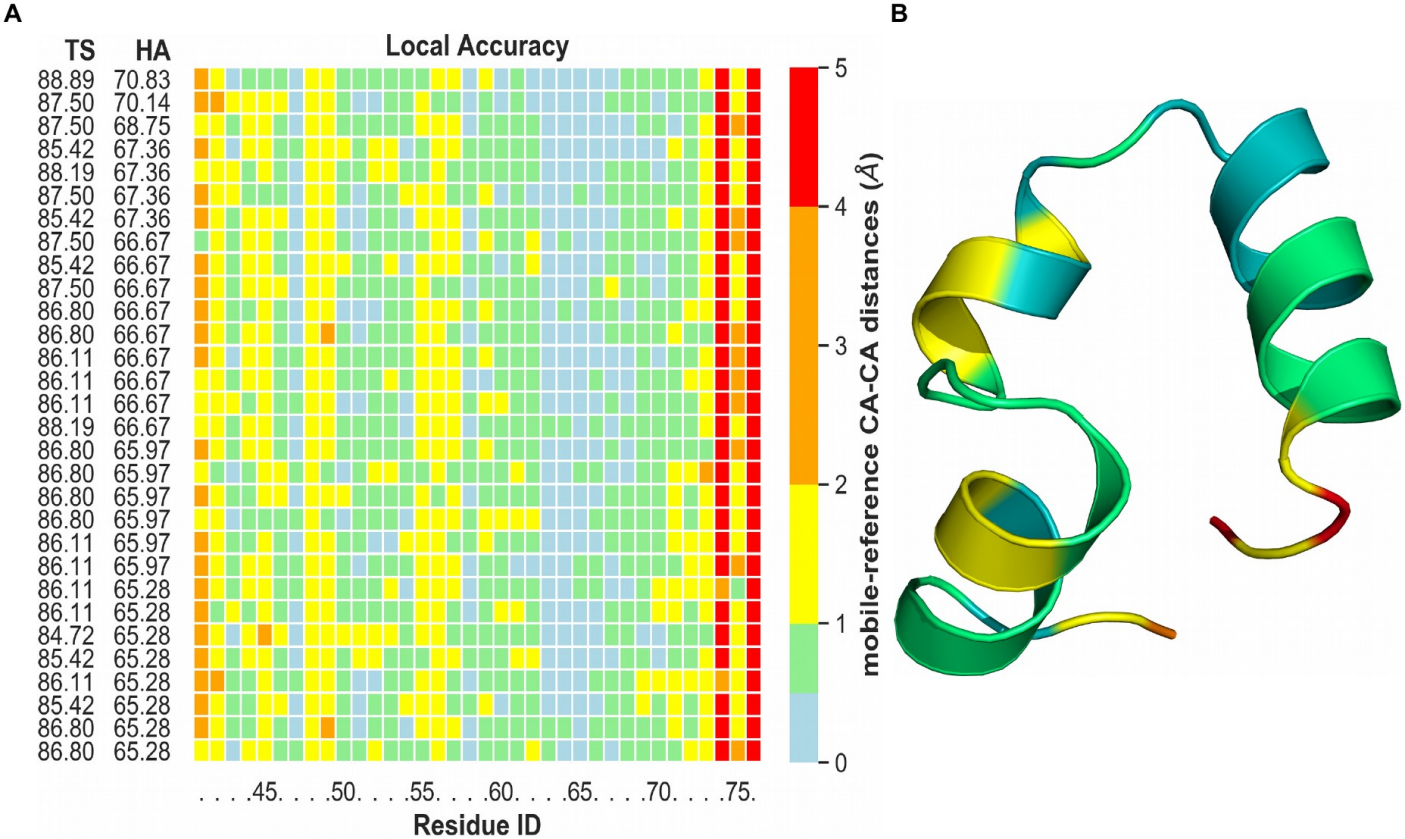
Local accuracy of VHP REMD simulation (36 contacts, 75% TPR). (A) Displayed are the best structures ranked by High Accuracy (HA) score and color-coded based on the C_*α*_-C_*α*_ distance to visualize the local accuracy. (B) Best observed tertiary structure of VHP corresponding to the first line of subfigure A.

To highlight the local accuracy and visualize how well the simulated structures fit the native state, Fig 8A gives an overview of the best observed structures during the 75% TPR simulation ranked by *GDT*_HA_. Each protein residue is assigned a colored rectangle representing the C_*α*_-C_*α*_ distance between the mobile and reference state after a least-squares fit. As evident from Fig 8A, many simulated structures greatly resemble the target structure, manifesting in C_*α*_ displacements below 2 Å. The best observed structure with an HA score of 70.83 corresponding to the first line is displayed in Fig 8B. For comparison, local accuracy figures of VHP with 36 restraints and varying TPR can be looked up in S44 Fig to S46 Fig in S1 Appendix.

## Conclusion

Contact information on its own usually is insufficient to fully determine a protein’s three-dimensional fold. Here we showed that including such information into replica-exchange molecular dynamics (REMD) delivers proper structural models of a protein’s conformational state by significantly enriching native-like folds. Our method combines a contact-based sigmoidal bias potential with the advantages of enhanced sampling in replica exchange, creating a high chance of observing (near-)native conformations within a single run. Contacts derived from various sources can easily be included and thus interpreted in terms of structural ensembles. As a physico-empirical method, contact-guided REMD is conceptually transparent in contrast to purely data-driven methods like AlphaFold [14] and does not require elaborate adjustment of *a priori* unknown model parameters.

To test the capabilities and limitations of our method, we presented a systematic study on two well-known proteins comprising 14 scenarios with different bias quality in terms of true-positive rate (TPR) and number of used contact restraints. In order to equally consider the influence of both native and non-native contacts, all restraints were assigned the same coupling strength. As long as the bias quality was above an apparent TPR threshold of 75%, significant enrichment of native-like conformations could be observed. We find that highly error-prone contact information as in 50% TPR scenarios is insufficient for effective structure determination within REMD. Typically, both experimentally and theoretically derived contact information can contain false positives, and it is *a priori* unknown which of the used contacts are actually native. In contact-guided REMD, negative effects resulting from false-positive contacts should already be minimized by design due to the sigmoid potential’s strength and range limitation. One approach to further resolve this issue is a dynamic weighting of contact bias. During a guided REMD simulation, contacts of initially equal strength can be monitored on a regular basis and those remaining unrealized may be weakened or deactivated accordingly. Furthermore, we evaluated the incremental improvement with increasing number of contacts for each set of scenarios at fixed TPR. For an error-free bias, the chance of finding native-like structures increases with the number of implemented contacts according to our expectations. In the more realistic cases with a 75% TPR, we observed significant performance improvements compared to the unrestrained reference when including *L*/2 to *L* contacts, with *L* being the protein sequence length. Taking DCA-derived contacts as an example for contact enrichment in a *de novo* prediction, the TPR strongly depends on the alignment quality and size of the investigated MSA. In view of the currently already rich and continuously fast growing protein sequence databases, TPRs of 75% are feasible for the first ranked *L* contacts [56].

To justify the comparably high computational cost of REMD resulting from simulating *N* replicas in parallel, we applied this empirical rule to both MD and REMD simulations each with and without contact bias. Within biased REMD, we obtained considerably better conformations in terms of RMSD more frequently, making this method feasible for obtaining (near-)native conformations in a single run. Even when performing multiple MD runs successively, finding native(-like) folds cannot be guaranteed due to the high chance of kinetic entrapment which can be overcome in REMD. Provided sufficient turnarounds and simulation time, we showed that contact-guided REMD is able to find physically reasonable folds starting from an unfolded state. Due to the high computational costs of REMD, it is beneficial to start from a pre-estimated folded state and use contact-guided REMD only for local refinement. We suggest this approach especially for larger proteins, as it can drastically reduce the system size. Various existing tools can be used to obtain pre-estimated structures. For example, protein decoys can be generated with PyRosetta [57] and subsequently be ranked according to their suitability as a starting conformation using Rosetta’s scoring functions. This is expected to greatly increase the computational performance of each REMD run. A further reduction of computational costs can be achieved by narrowing down the temperature range to, e.g., 280 to 430 K. This will, however, effectively lower the chances of large-scale conformational change as the reservoir of unfolded conformations is reduced. Lastly, it is also possible to lower the computational demands by using an implicit-solvent model.

Although computationally rather involved, we consider contact-guided REMD to be particularly suitable for final refinement of often less resolved local structural motifs or available low-resolution structural models. Underlying MD force fields contain rich information on the various physical interactions determining protein dynamics. Since the bias is known, it can retrospectively be balanced out. Thus, it is also possible to infer a free energy landscape with statistical techniques such as the weighted histogram analysis method [58] using the whole data set instead of focusing on the lowest-temperature replicas exclusively.

## Supporting information

S1 Appendix Collection of all supplementary figures. (PDF)

S2 Appendix Temperature distributions of REMD simulations. (PDF)

S3 Appendix Sample mdp file for REMD simulations. (PDF)

S4 Appendix Sample mdp file for MD simulations. (PDF)

S1 File Minimal dataset. (ZIP)
