# Supplementary material for: Including residual contact information into replica-exchange MD simulations significantly enriches native-like conformations
Source: PLoS One. 2020 Nov 16;15(11):e0242072. doi: 10.1371/journal.pone.0242072 (PMC7668583; doi:10.1371/journal.pone.0242072)
Supplement: S1 Appendix — (PDF) [file pone.0242072.s001.pdf]

Supporting Information

Including residual contact information into replica-exchange MD simulations significantly enriches native-like conformations

Arthur Voronin, Marie Weiel, Alexander Schug

S1 Appendix: Collection of all supplementary figures

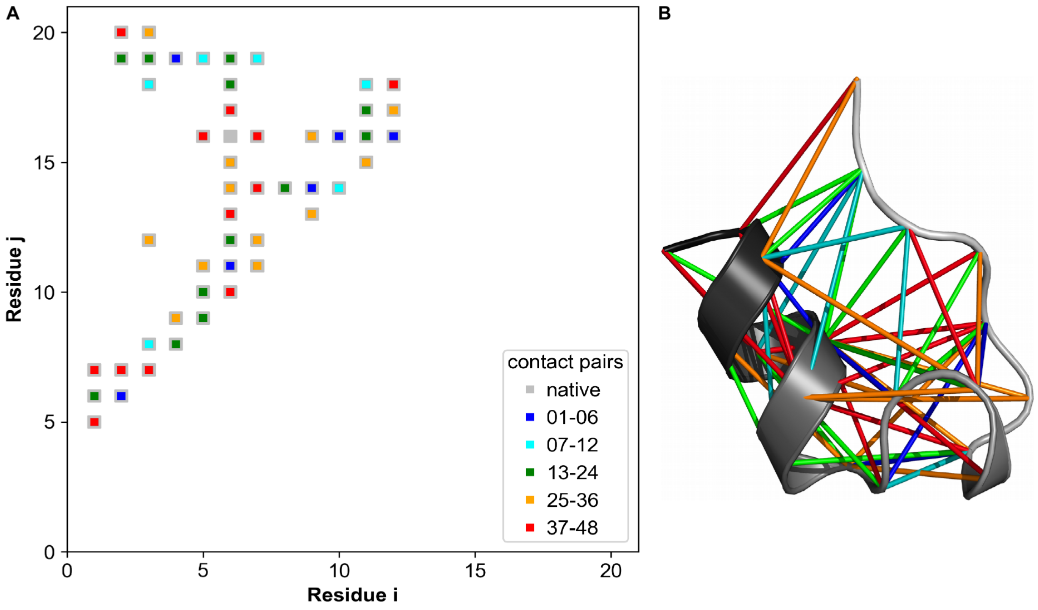

S1 Fig. Restraints used in Trp-Cage REMD simulations at 100% TPR.
(A) Contact map displays native contacts as gray squares. Randomly selected contact pairs which were used as restraints are colored based on their batch. (B) Tertiary structure of Trp-Cage showing the contact pairs in the same color as in the contact map.

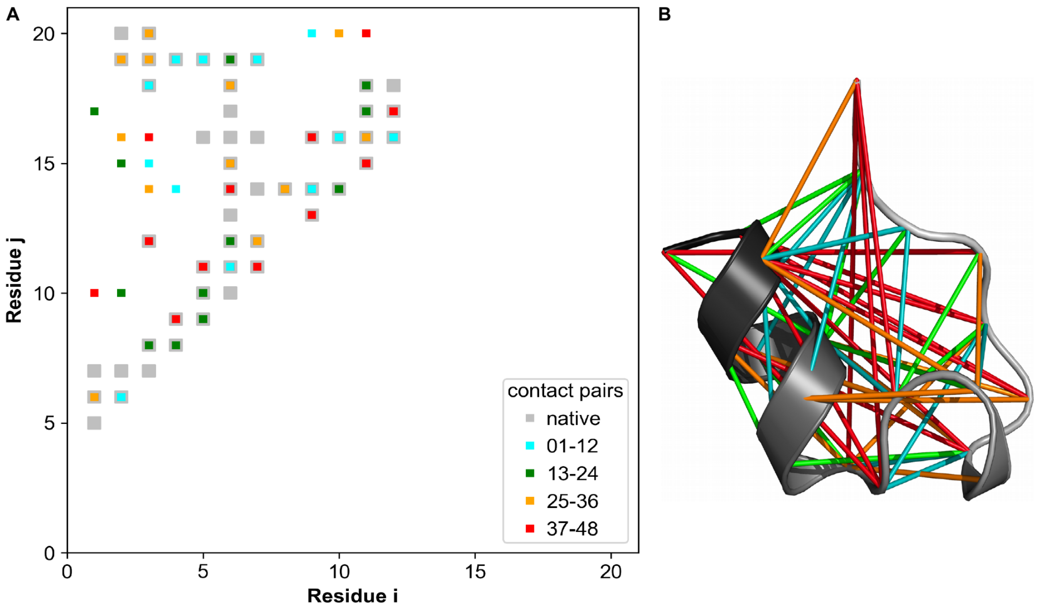

S2 Fig. Restraints used in Trp-Cage REMD simulations at 75% TPR.
(A) Contact map displays native contacts as gray squares. Randomly selected contact pairs which were used as restraints are colored based on their batch. (B) Tertiary structure of Trp-Cage showing the contact pairs in the same color as in the contact map.

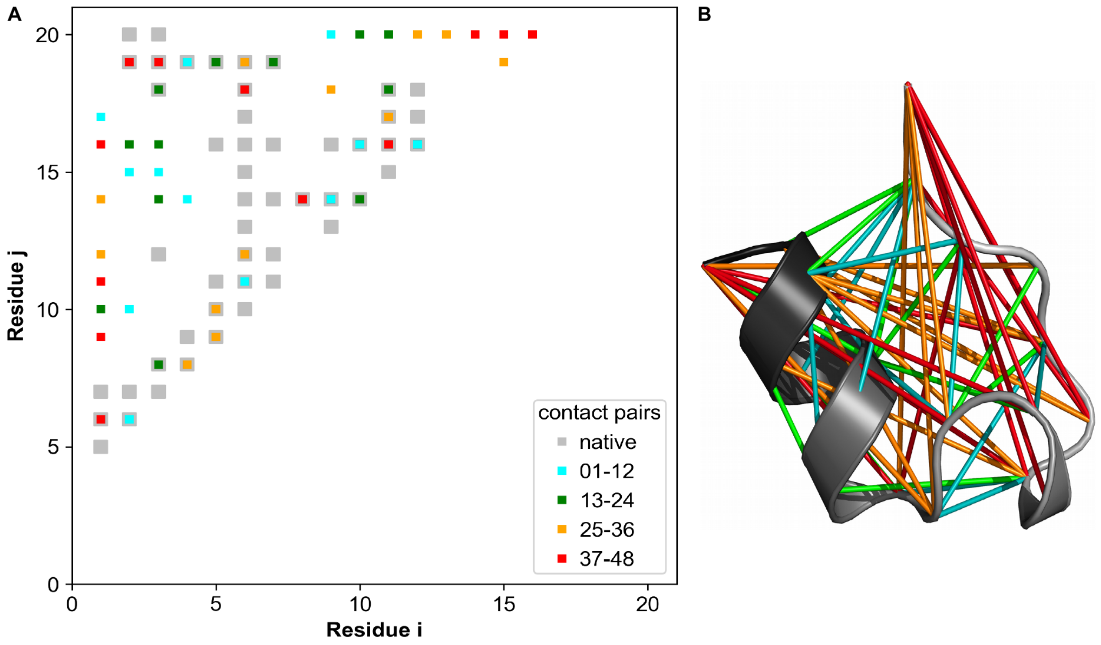

S3 Fig. Restraints used in Trp-Cage REMD simulations at 50% TPR.
(A) Contact map displays native contacts as gray squares. Randomly selected contact pairs which were used as restraints are colored based on their batch. (B) Tertiary structure of Trp-Cage showing the contact pairs in the same color as in the contact map.

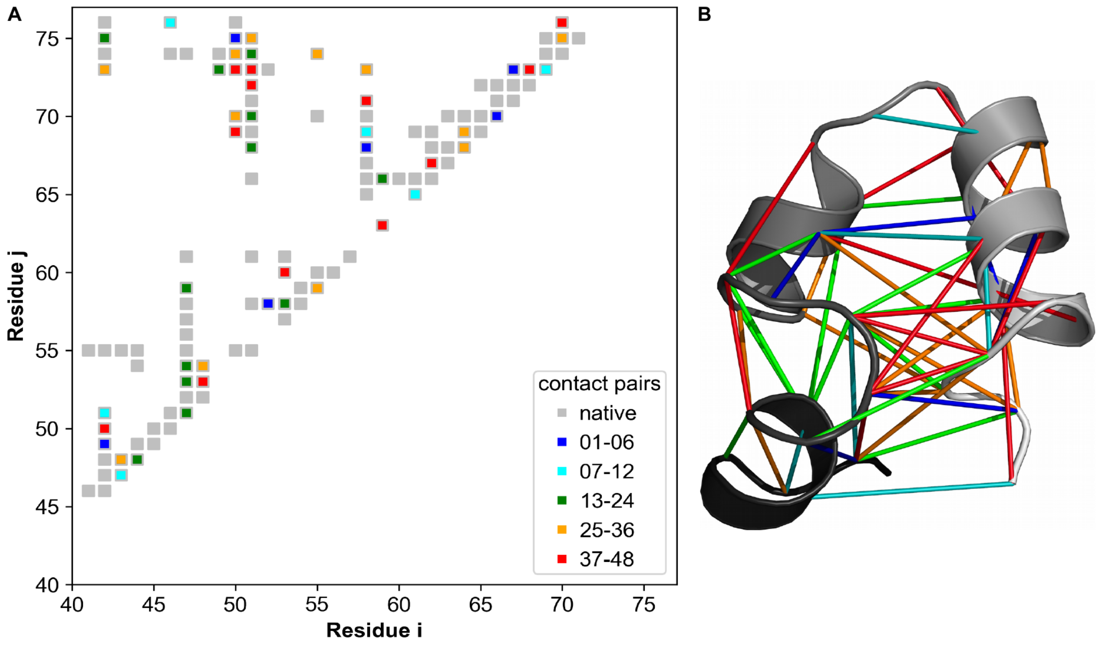

S4 Fig. Restraints used in VHP REMD simulations at 100% TPR.
(A) Contact map displays native contacts as gray squares. Randomly selected contact pairs which were used as restraints are colored based on their batch. (B) Tertiary structure of VHP showing the contact pairs in the same color as in the contact map.

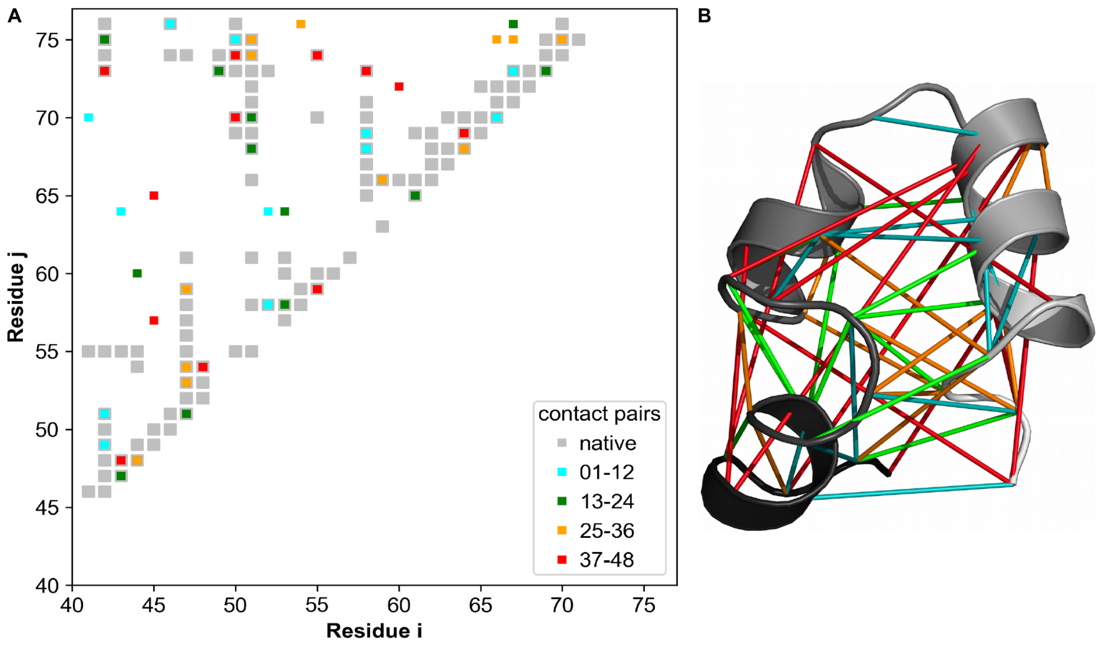

S5 Fig. Restraints used in VHP REMD simulations at 75% TPR.
(A) Contact map displays native contacts as gray squares. Randomly selected contact pairs which were used as restraints are colored based on their batch. (B) Tertiary structure of VHP showing the contact pairs in the same color as in the contact map.

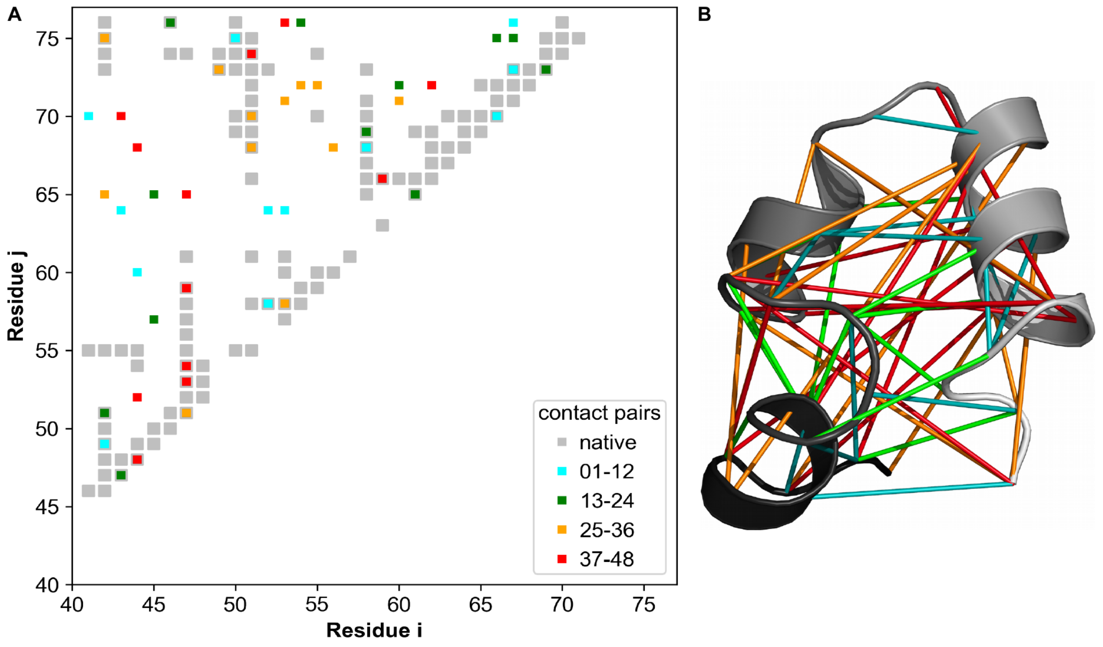

S6 Fig. Restraints used in VHP REMD simulations at 50% TPR.
(A) Contact map displays native contacts as gray squares. Randomly selected contact pairs which were used as restraints are colored based on their batch. (B) Tertiary structure of VHP showing the contact pairs in the same color as in the contact map.

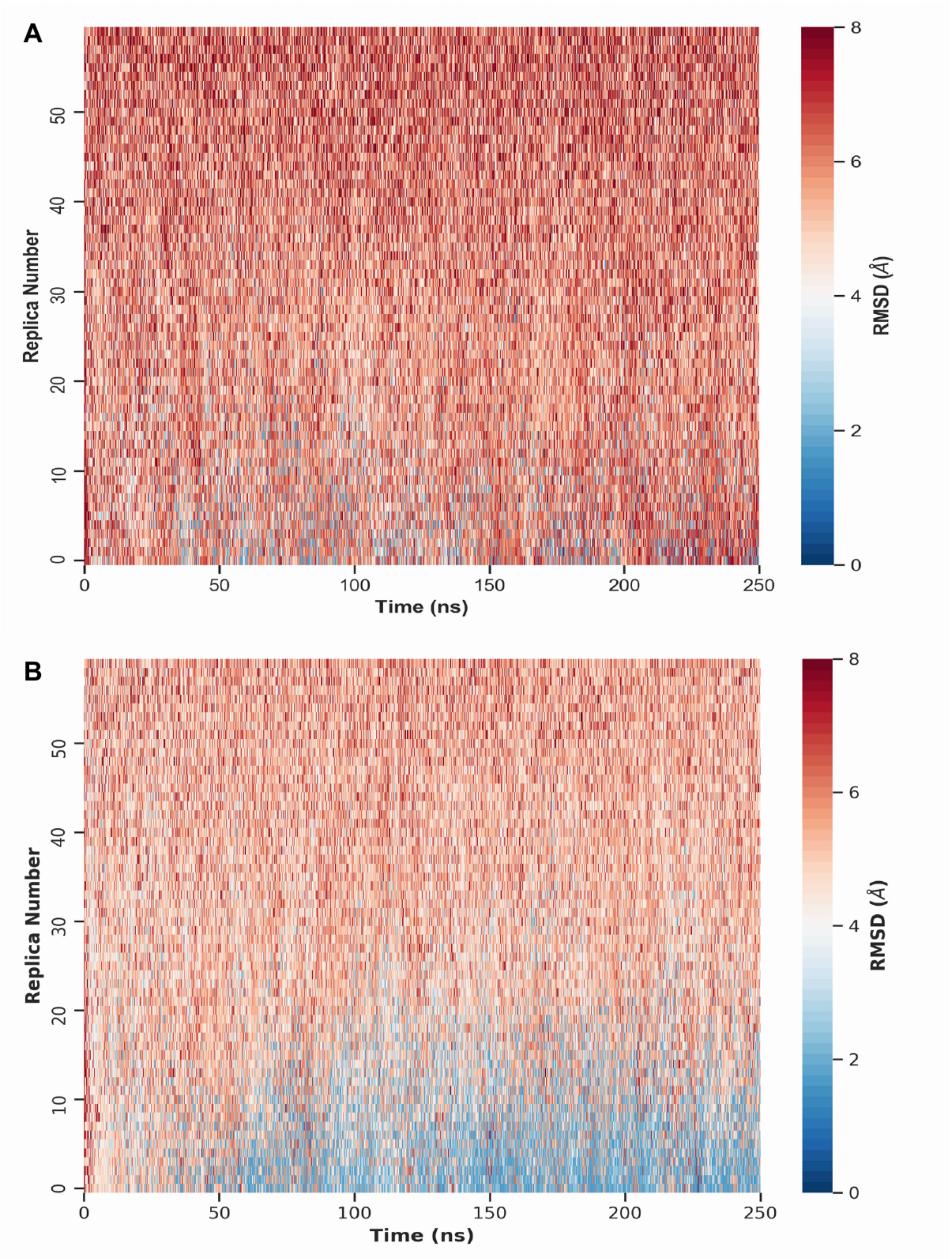

S7 Fig. RMSD overview of Trp-Cage REMD simulations.
Heatmaps display the backbone RMSD across all replicas. (A) Reference REMD simulation without any additional bias. (B) REMD simulation with 100% TPR and 6 native restraining contacts.

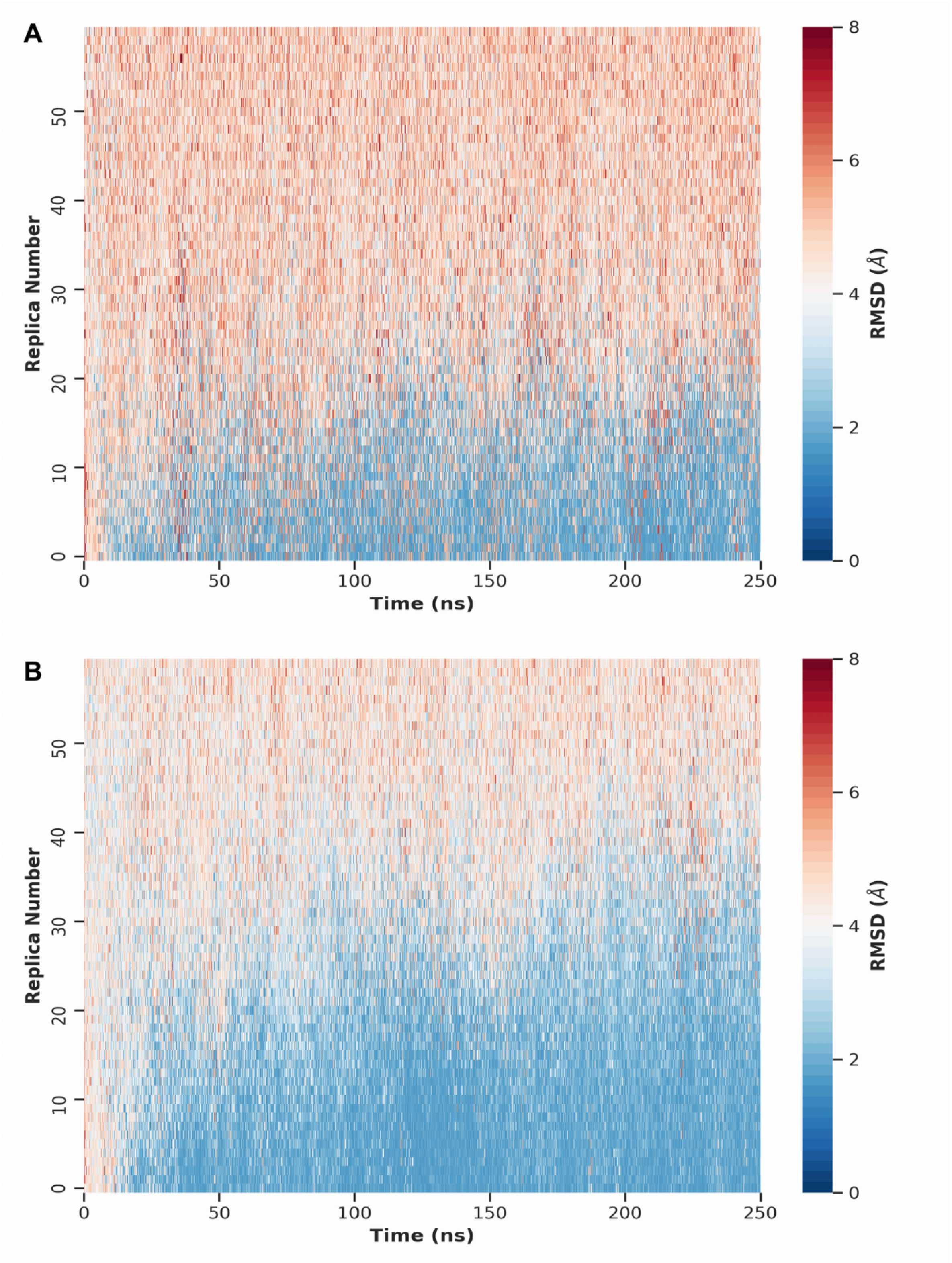

S8 Fig. RMSD overview of Trp-Cage REMD simulations.
Heatmaps display the backbone RMSD across all replicas. (A) REMD simulation with 100% TPR and 12 native restraining contacts. (B) REMD simulation with 100% TPR and 24 native restraining contacts.

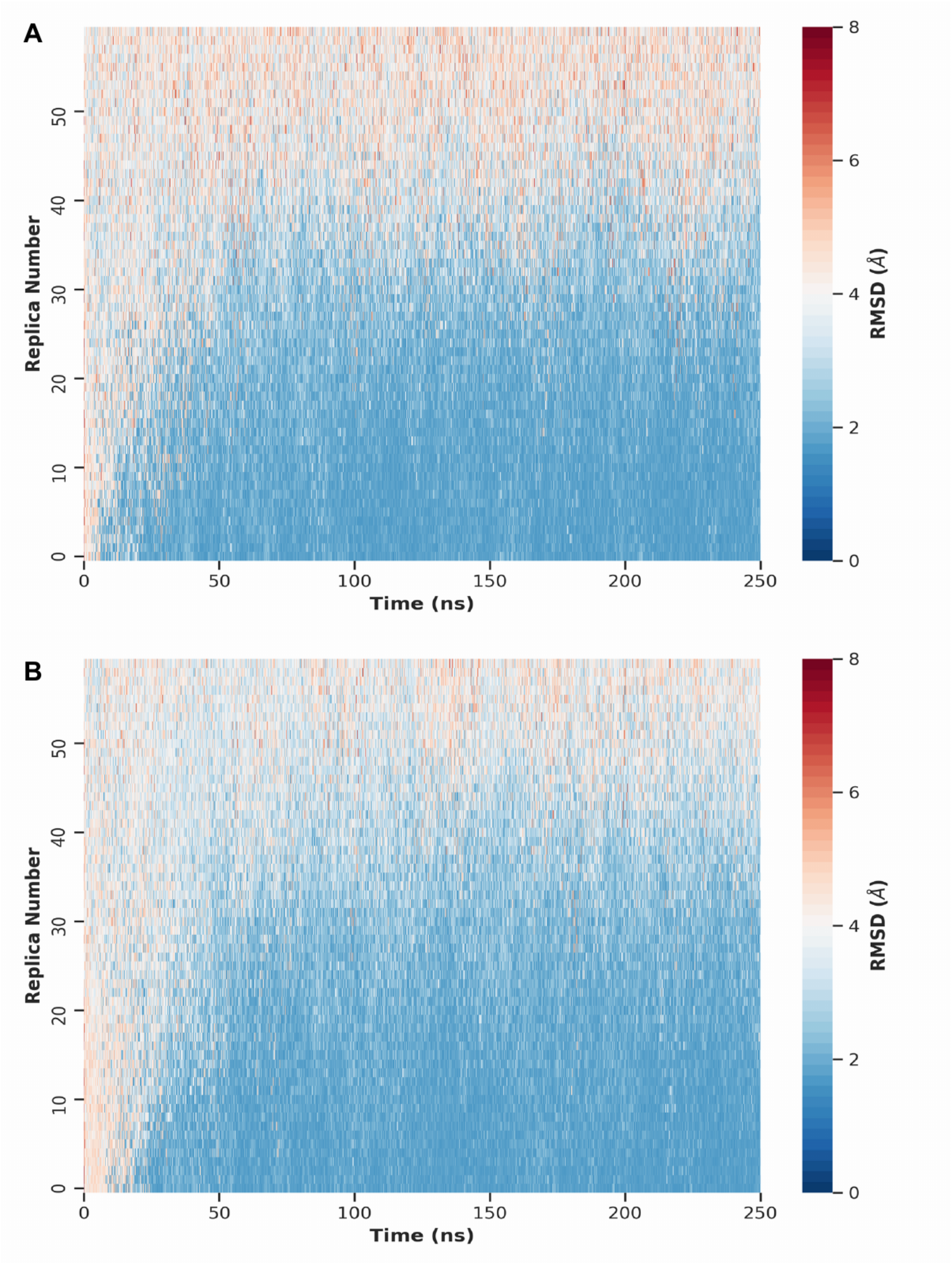

S9 Fig. RMSD overview of Trp-Cage REMD simulations.
Heatmaps display the backbone RMSD across all replicas. (A) REMD simulation with 100% TPR and 36 native restraining contacts. (B) REMD simulation with 100% TPR and 48 native restraining contacts.

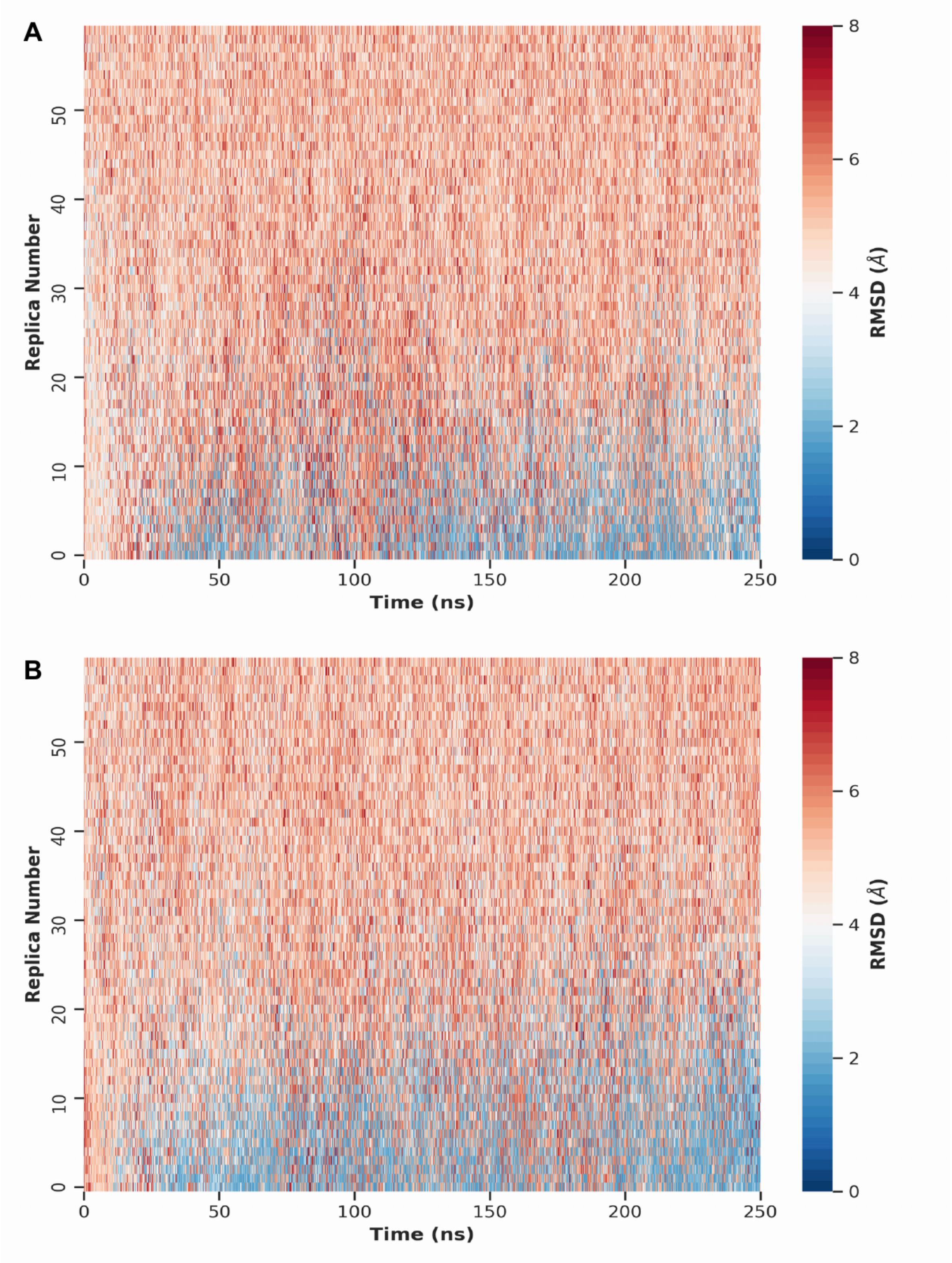

S10 Fig. RMSD overview of Trp-Cage REMD simulations.

Heatmaps display the backbone RMSD across all replicas. (A) REMD simulation with 75% TPR and 12 restraining contacts (9 native, 3 non-native). (B) REMD simulation with 75% TPR and 24 restraining contacts (18 native, 6 non-native).

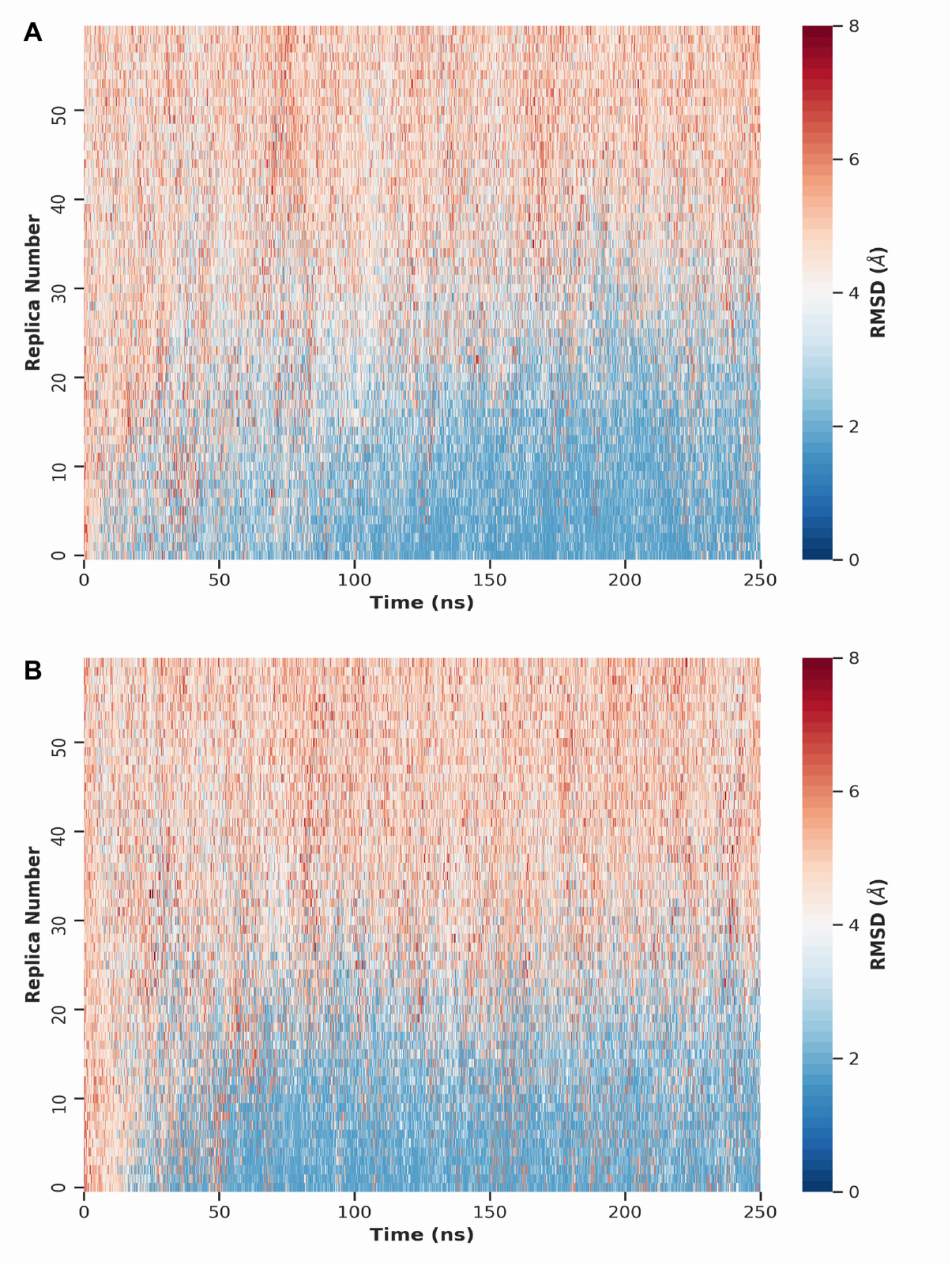

S11 Fig. RMSD overview of Trp-Cage REMD simulations.
Heatmaps display the backbone RMSD across all replicas. (A) REMD simulation with 75% TPR and 36 restraining contacts (27 native, 9 non-native). (B) REMD simulation with 75% TPR and 48 restraining contacts (36 native, 12 non-native).

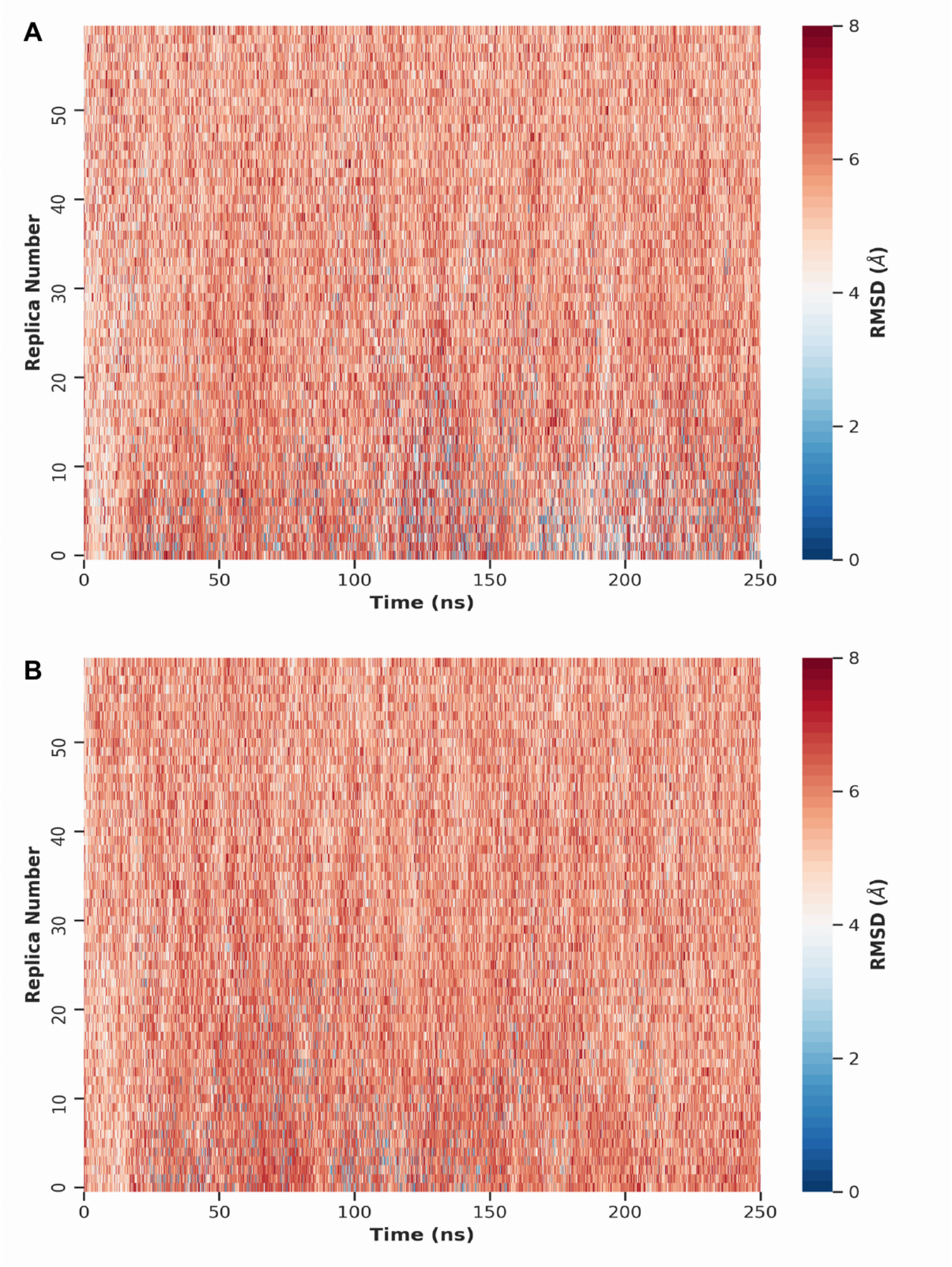

S12 Fig. RMSD overview of Trp-Cage REMD simulations.
Heatmaps display the backbone RMSD time evolution across all replicas. (A) REMD simulation with 50% TPR and 12 restraining contacts (6 native, 6 non-native). (B) REMD simulation with 50% TPR and 24 restraining contacts (12 native, 12 non-native).

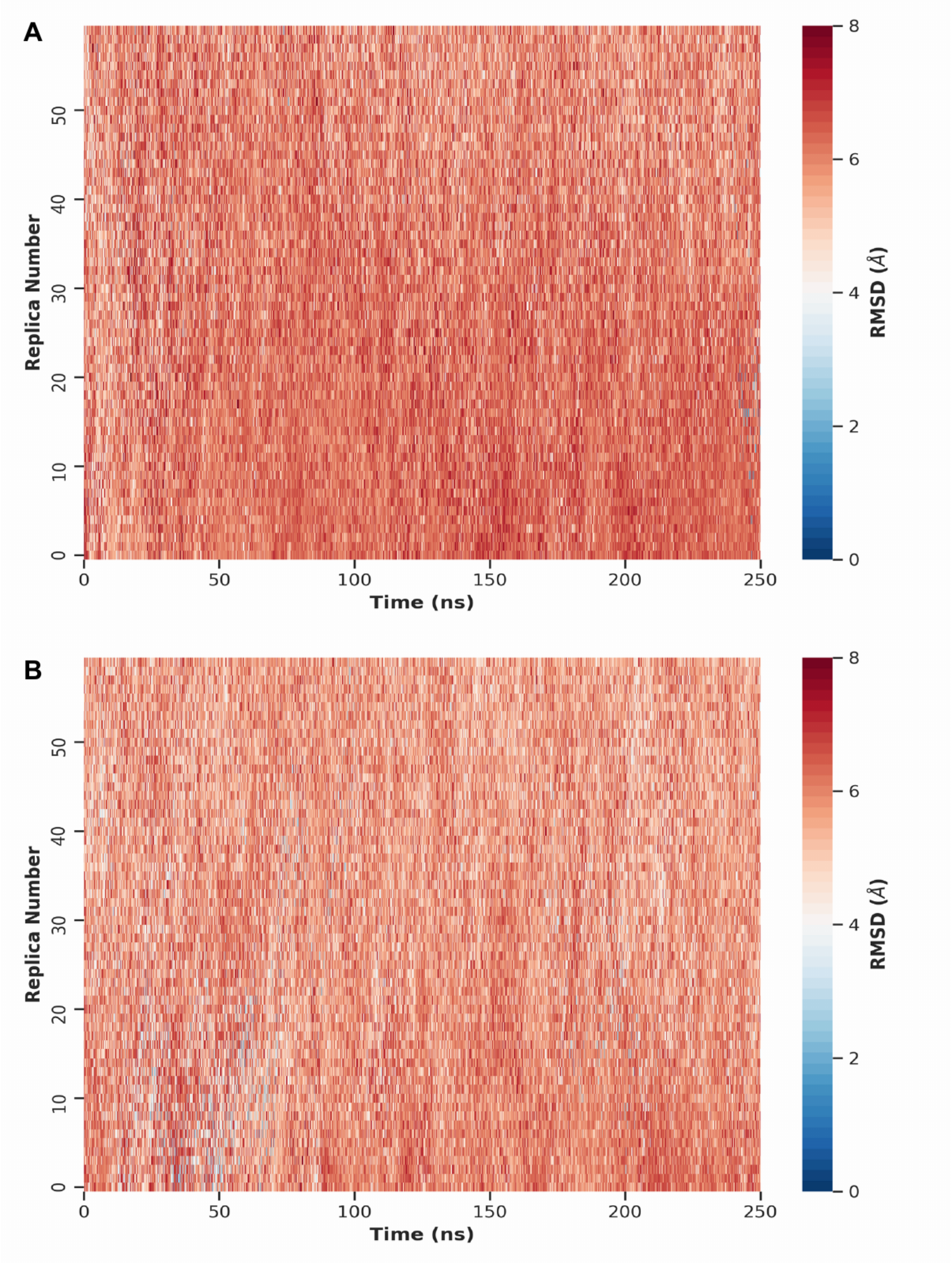

S13 Fig. RMSD overview of Trp-Cage REMD simulations.
Heatmaps display the backbone RMSD across all replicas. (A) REMD simulation with 50% TPR and 36 restraining contacts (18 native, 18 non-native). (B) REMD simulation with 50% TPR and 48 restraining contacts (24 native, 24 non-native).

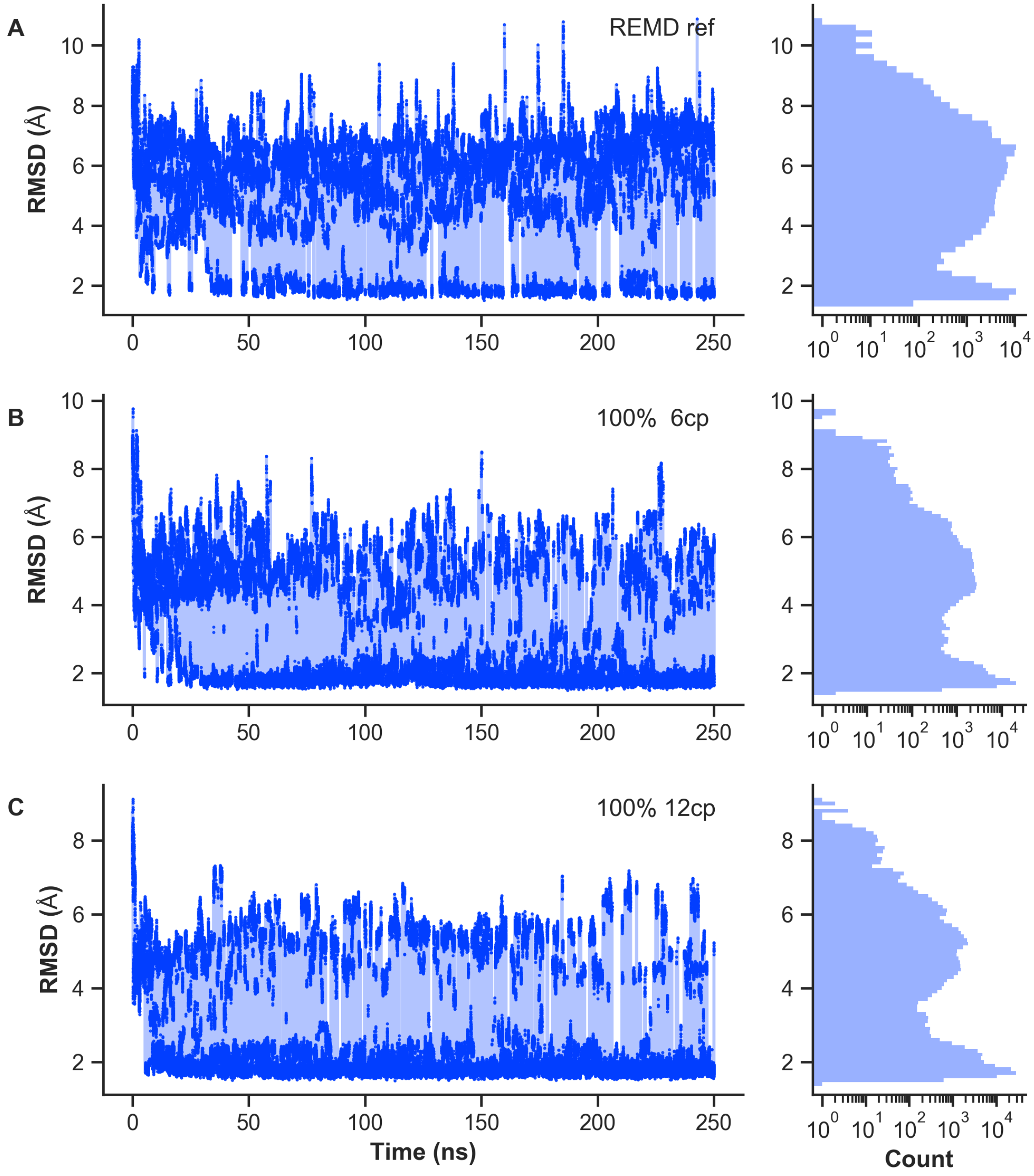

S14 Fig. RMSD curve and histogram of Trp-Cage REMD simulation.
Figure shows the backbone RMSD time evolution of the lowest-temperature replica at $T_0 = 300\,\mathrm{K}$ and the corresponding histogram with logarithmic count axis. (A) Reference REMD simulation without additional bias. (B) REMD simulation with 100% TPR and 6 native restraining contacts. (C) REMD simulation with 100% TPR and 12 native restraining contacts.

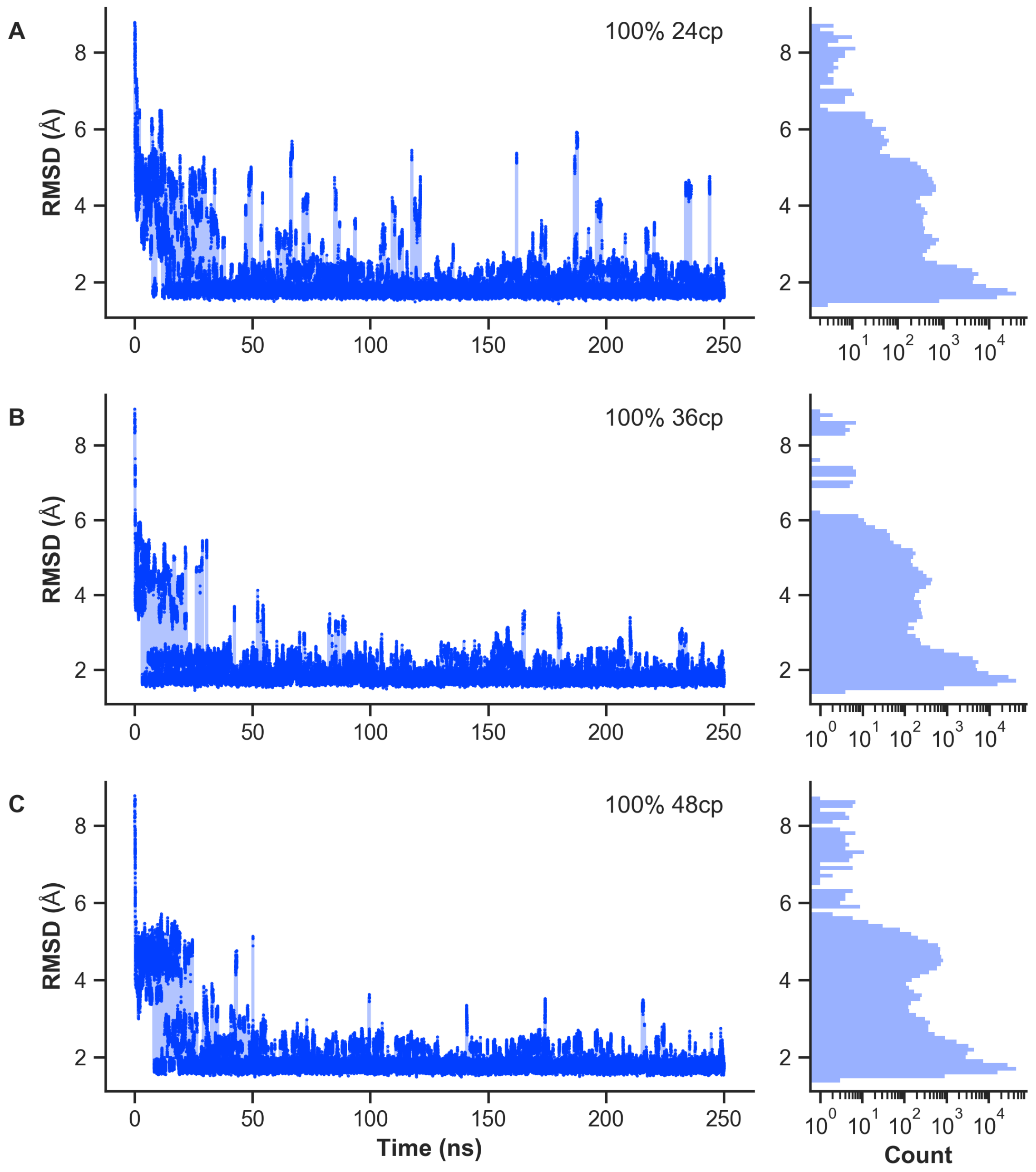

S15 Fig. RMSD curve and histogram of Trp-Cage REMD simulation.
Figure shows the backbone RMSD time evolution of the lowest-temperature replica at $T_0 = 300\,\mathrm{K}$ and the corresponding histogram with logarithmic count axis. (A) REMD simulation with 100% TPR and 24 native restraining contacts. (B) REMD simulation with 100% TPR and 36 native restraining contacts. (C) REMD simulation with 100% TPR and 48 native restraining contacts.

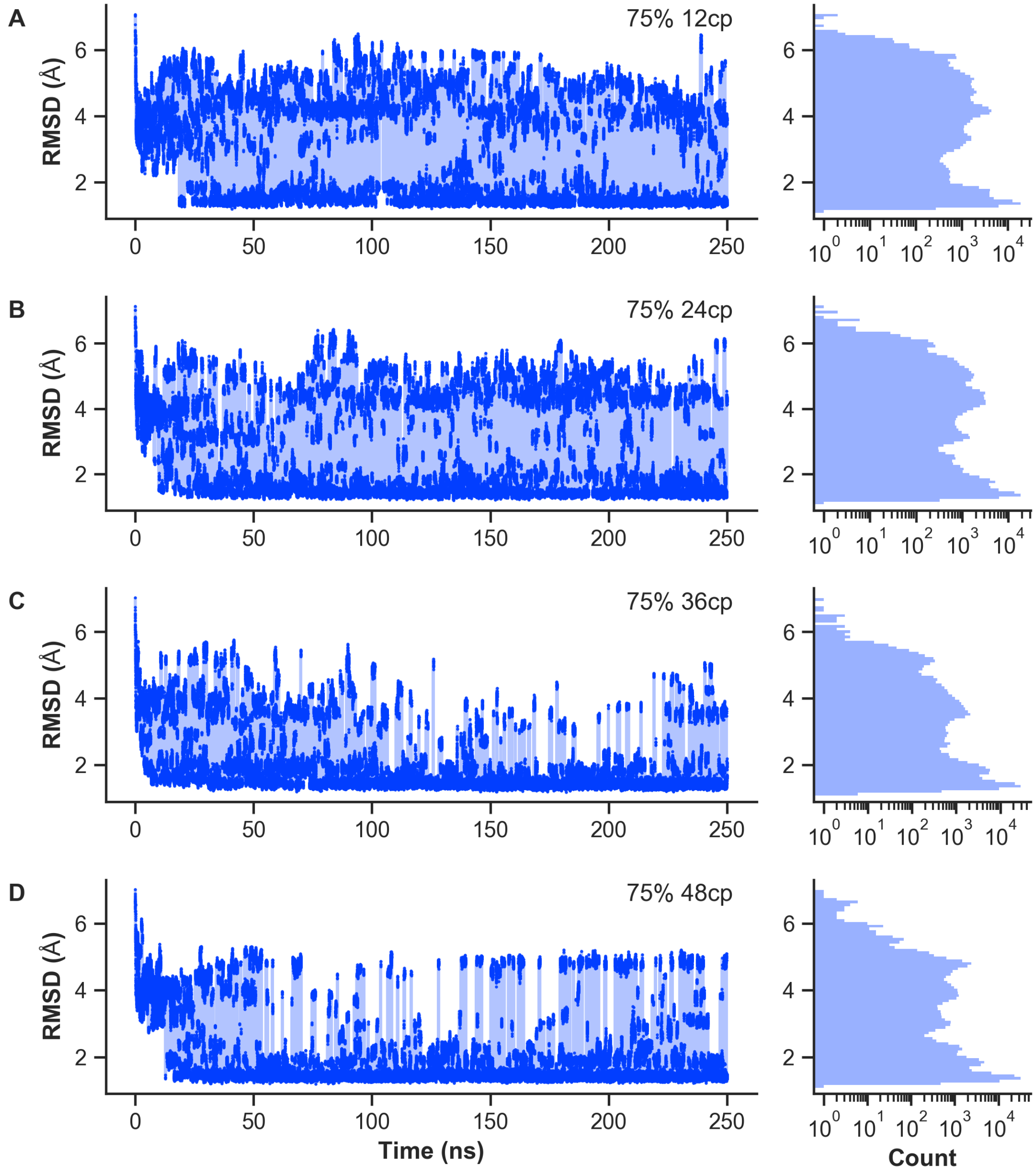

S16 Fig. RMSD curve and histogram of Trp-Cage REMD simulation.
Figure shows the backbone RMSD time evolution of the lowest-temperature replica at $T_0 = 300\,\mathrm{K}$ and the corresponding histogram with logarithmic count axis. (A) REMD simulation with 75% TPR and 12 restraining contacts (9 native, 3 non-native) (B) REMD simulation with 75% TPR and 24 restraining contacts (18 native, 6 non-native) (C) REMD simulation with 75% TPR and 36 restraining contacts (27 native, 9 non-native). (D) REMD simulation with 75% TPR and 48 restraining contacts (36 native, 12 non-native).

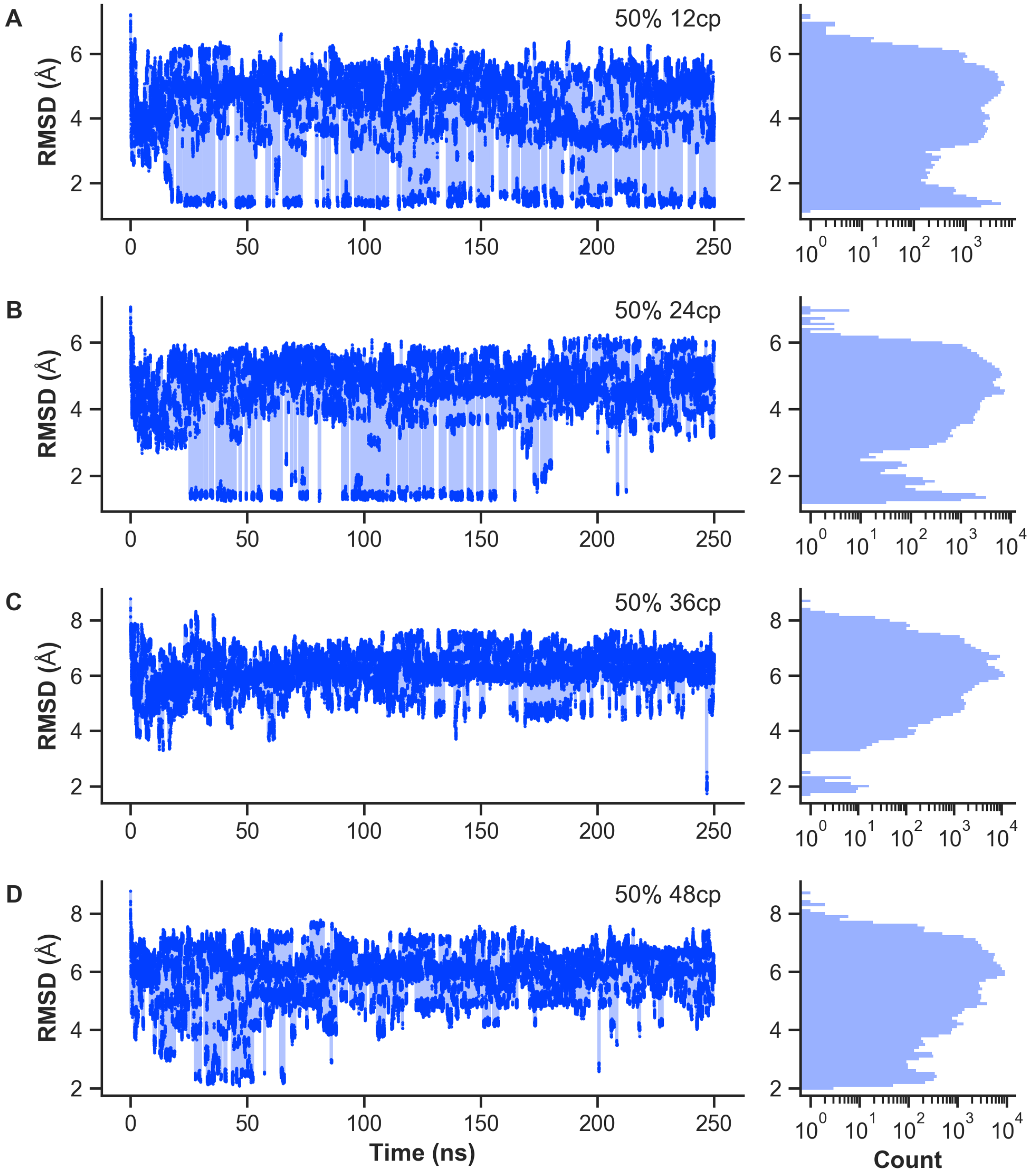

S17 Fig. RMSD curve and histogram of Trp-Cage REMD simulation.
Figure shows the backbone RMSD time evolution of the lowest-temperature replica at $T_0 = 300\,\mathrm{K}$ and the corresponding histogram with logarithmic count axis. (A) REMD simulation with 50% TPR and 12 restraining contacts (6 native, 6 non-native) (B) REMD simulation with 50% TPR and 24 restraining contacts (12 native, 12 non-native) (C) REMD simulation with 50% TPR and 36 restraining contacts (18 native, 18 non-native). (D) REMD simulation with 50% TPR and 48 restraining contacts (24 native, 24 non-native).

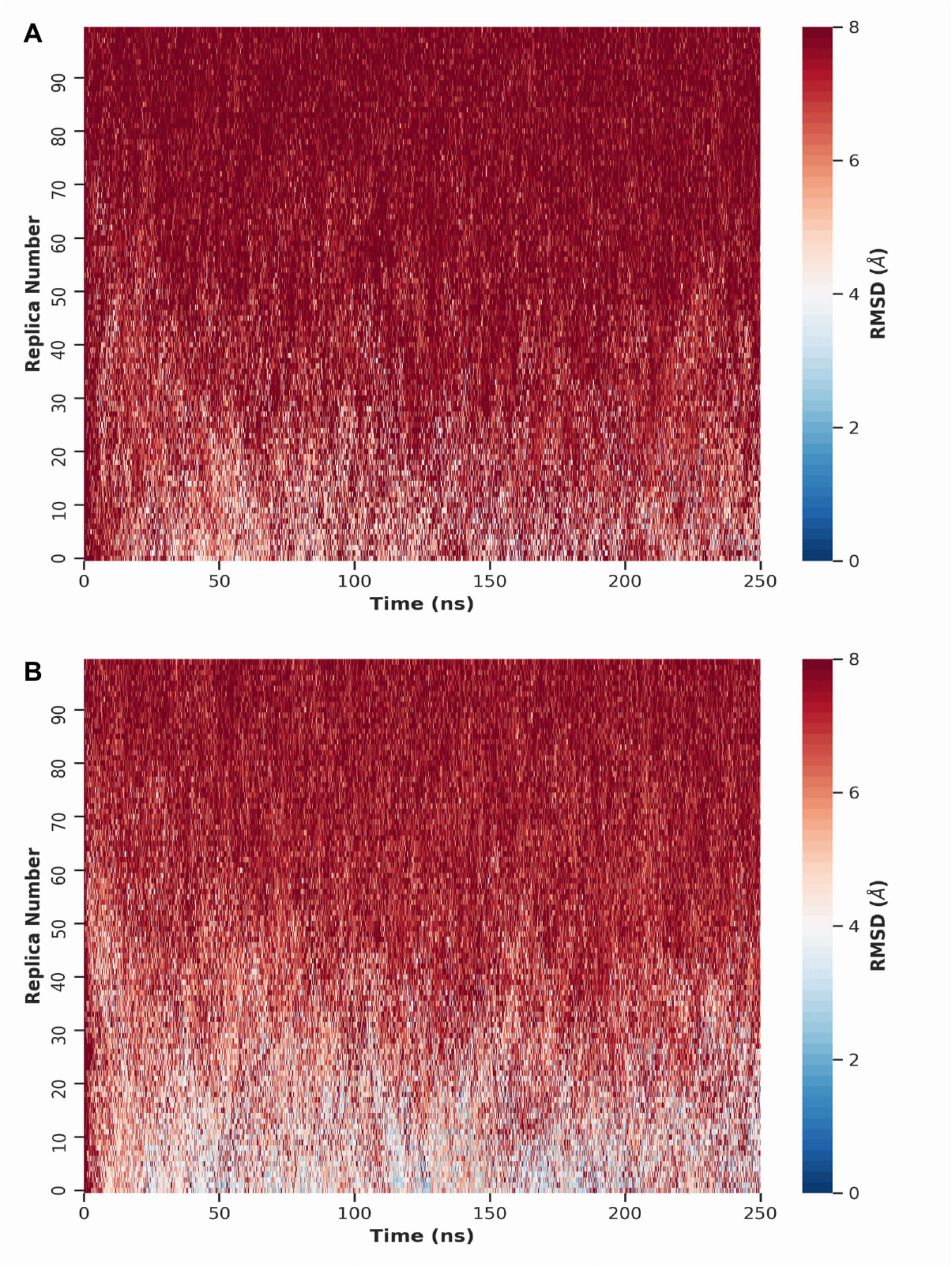

S18 Fig. RMSD overview of VHP REMD simulations.
Heatmaps display the backbone RMSD across all replicas. (A) Reference REMD simulation without additional bias. (B) REMD simulation with 100% TPR and 6 native restraining contacts.

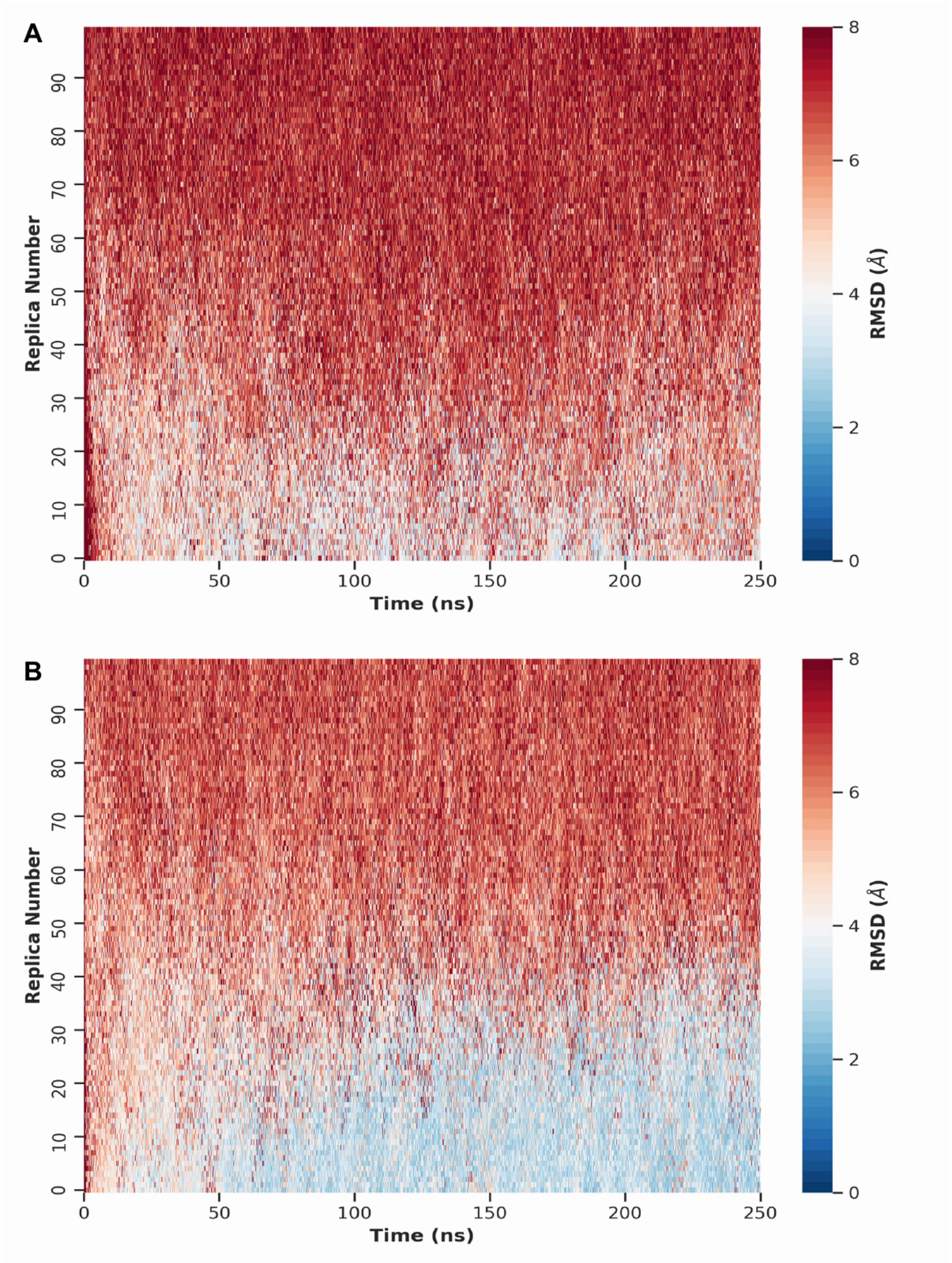

S19 Fig. RMSD overview of VHP REMD simulations.
Heatmaps display the backbone RMSD across all replicas. (A) REMD simulation with 100% TPR and 12 native restraining contacts. (B) REMD simulation with 100% TPR and 24 native restraining contacts.

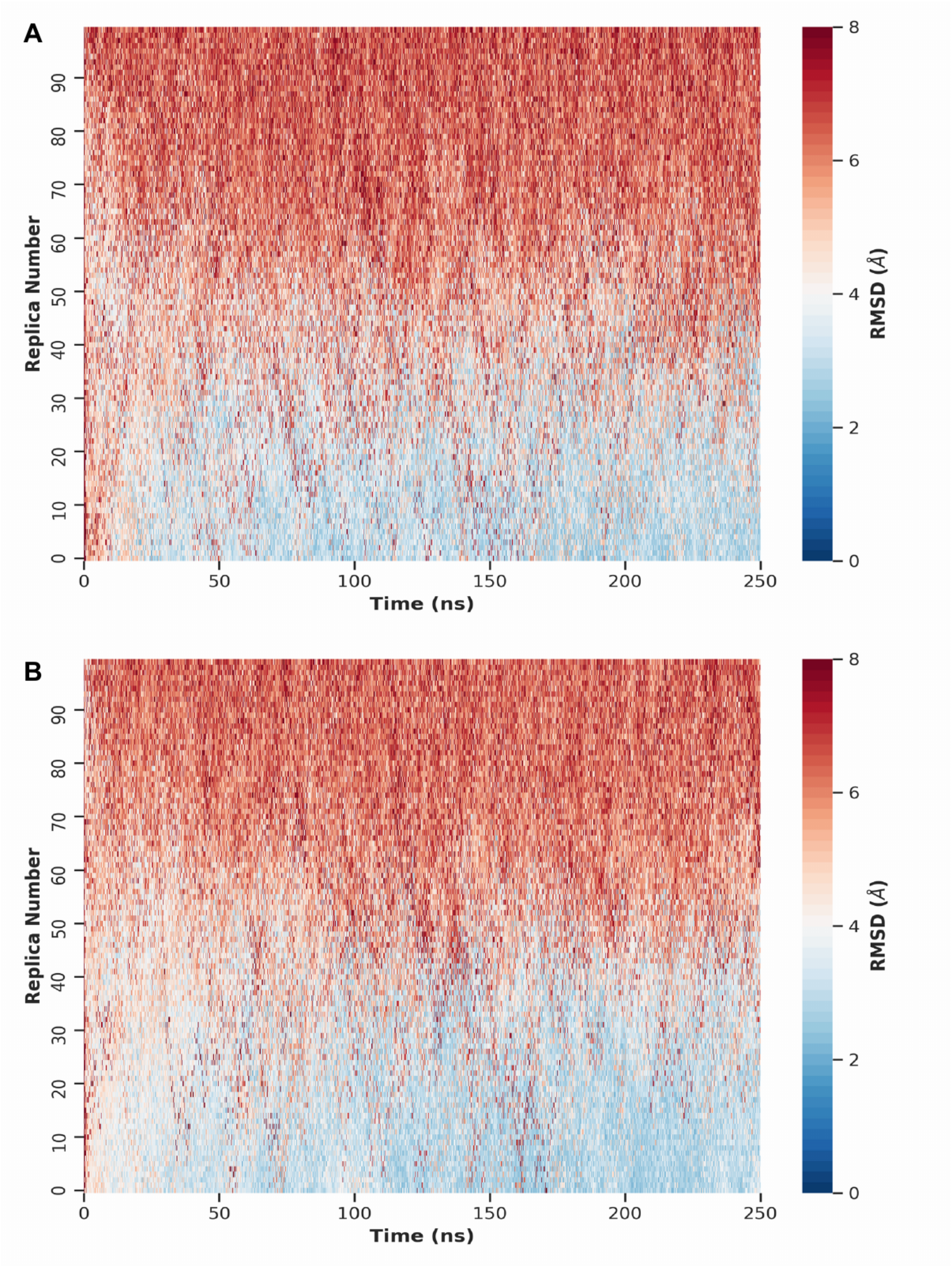

S20 Fig. RMSD overview of VHP REMD simulations.

Heatmaps display the backbone RMSD across all replicas. (A) REMD simulation with 100% TPR and 36 native restraining contacts. (B) REMD simulation with 100% TPR and 48 native restraining contacts.

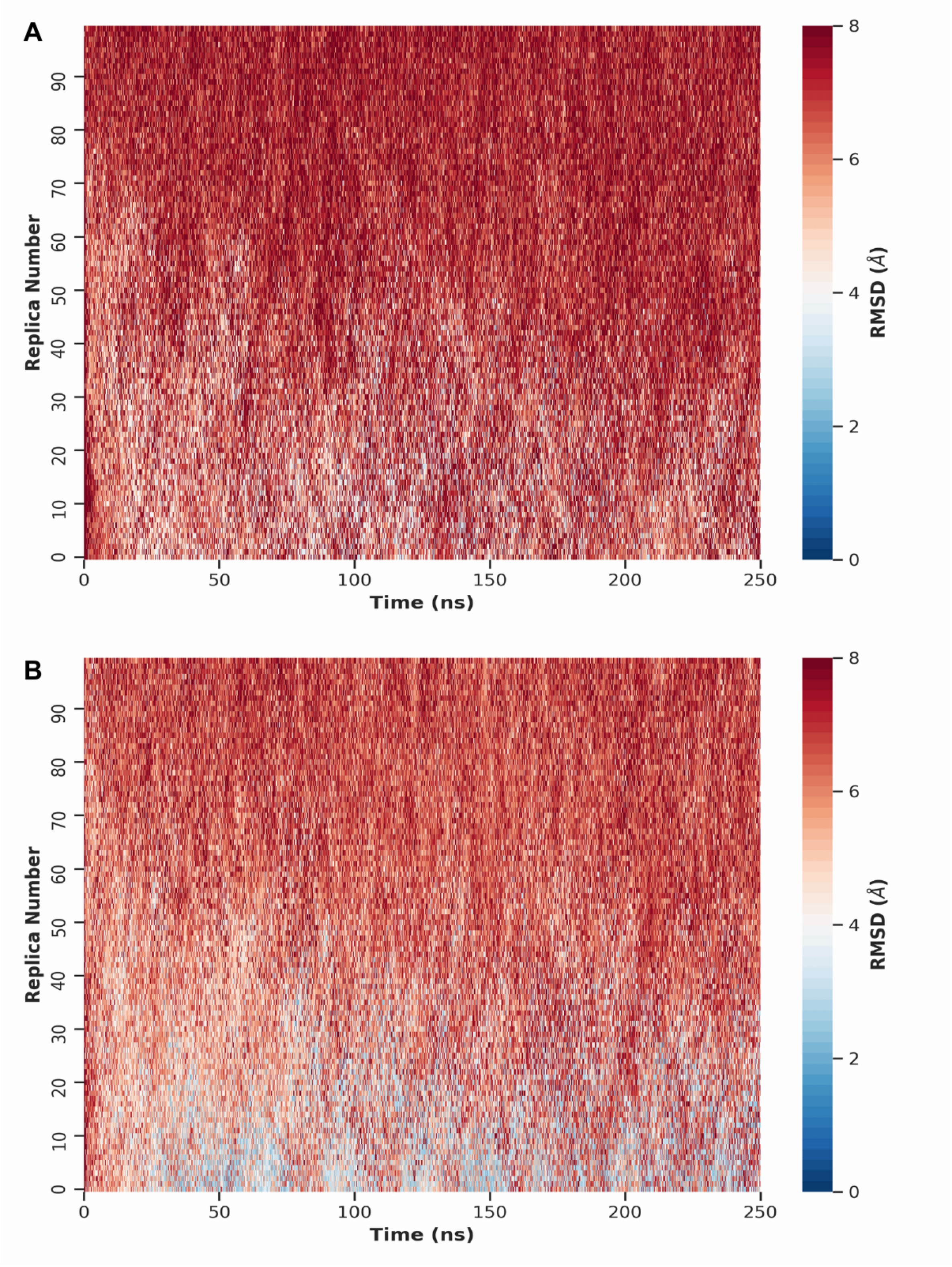

S21 Fig. RMSD overview of VHP REMD simulations.
Heatmaps display the backbone RMSD across all replicas. (A) REMD simulation with 75% TPR and 12 restraining contacts (9 native, 3 non-native). (B) REMD simulation with 75% TPR and 24 restraining contacts (18 native, 6 non-native).

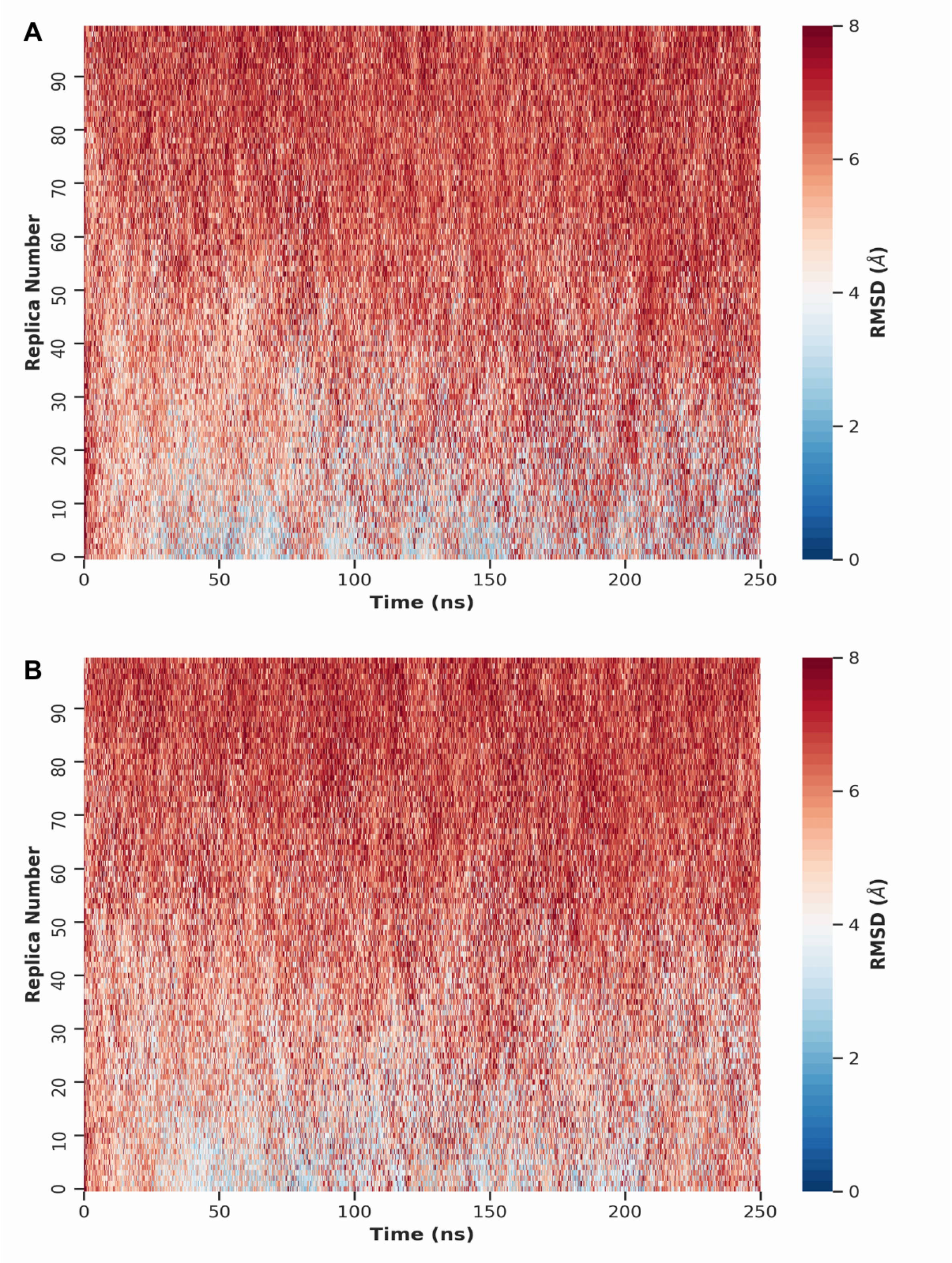

S22 Fig. RMSD overview of VHP REMD simulations.
Heatmaps display the backbone RMSD across all replicas. (A) REMD simulation with 75% TPR and 36 restraining contacts (27 native, 9 non-native). (B) REMD simulation with 75% TPR and 48 restraining contacts (36 native, 12 non-native).

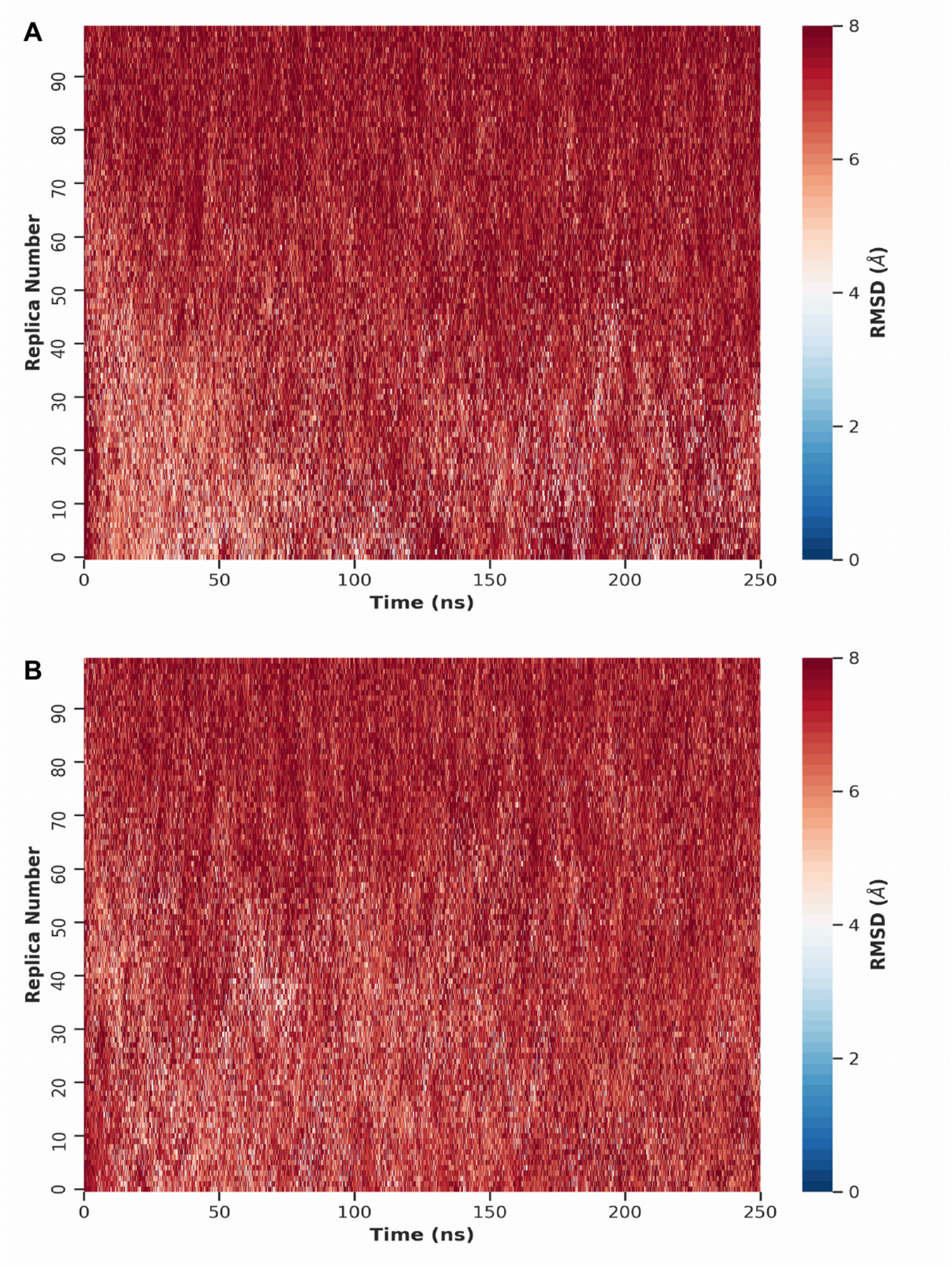

S23 Fig. RMSD overview of VHP REMD simulations.
Heatmaps display the backbone RMSD across all replicas. (A) REMD simulation with 50% TPR and 12 restraining contacts (6 native, 6 non-native). (B) REMD simulation with 50% TPR and 24 restraining contacts (12 native, 12 non-native).

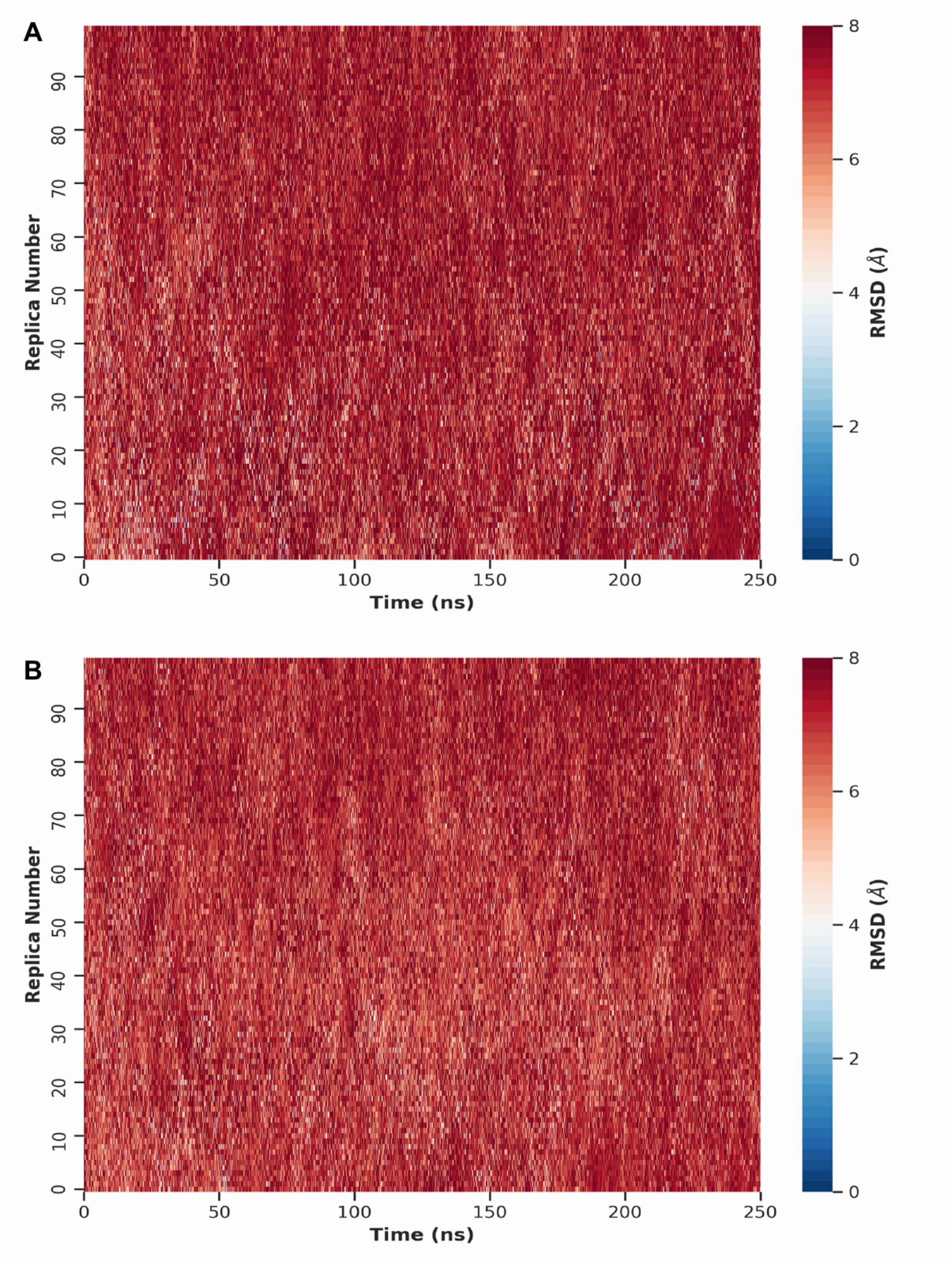

S24 Fig. RMSD overview of VHP REMD simulations.
Heatmaps display the backbone RMSD across all replicas. (A) REMD simulation with 50% TPR and 36 restraining contacts (18 native, 18 non-native). (B) REMD simulation with 50% TPR and 48 restraining contacts (24 native, 24 non-native).

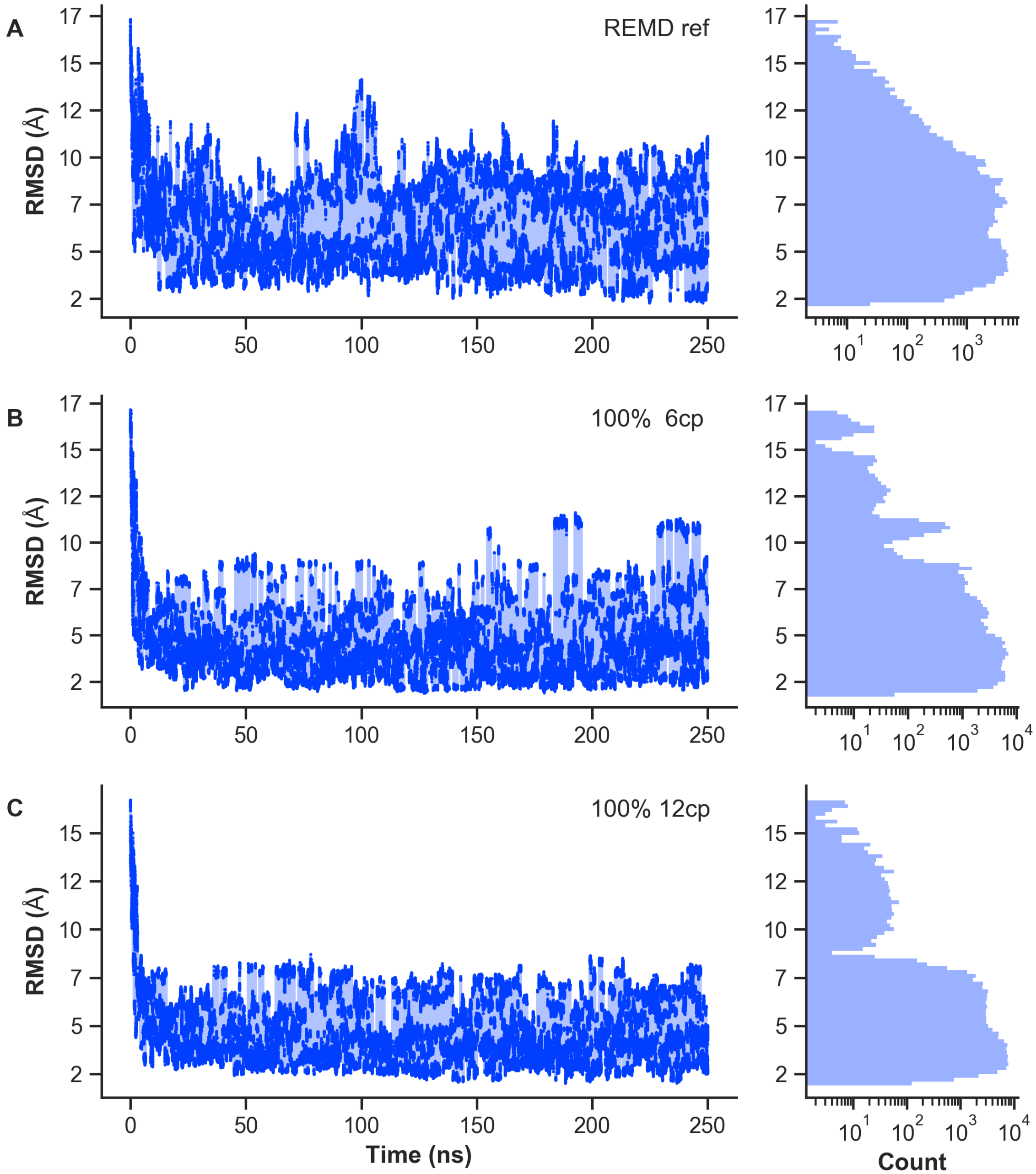

S25 Fig. RMSD curve and histogram of VHP REMD simulation.
Figure shows the backbone RMSD time evolution of the lowest-temperature replica at $T_0 = 300\,\mathrm{K}$ and the corresponding histogram with logarithmic count axis. (A) Reference REMD simulation without additional bias. (B) REMD simulation with 100% TPR and 6 native restraining contacts. (C) REMD simulation with 100% TPR and 12 native restraining contacts.

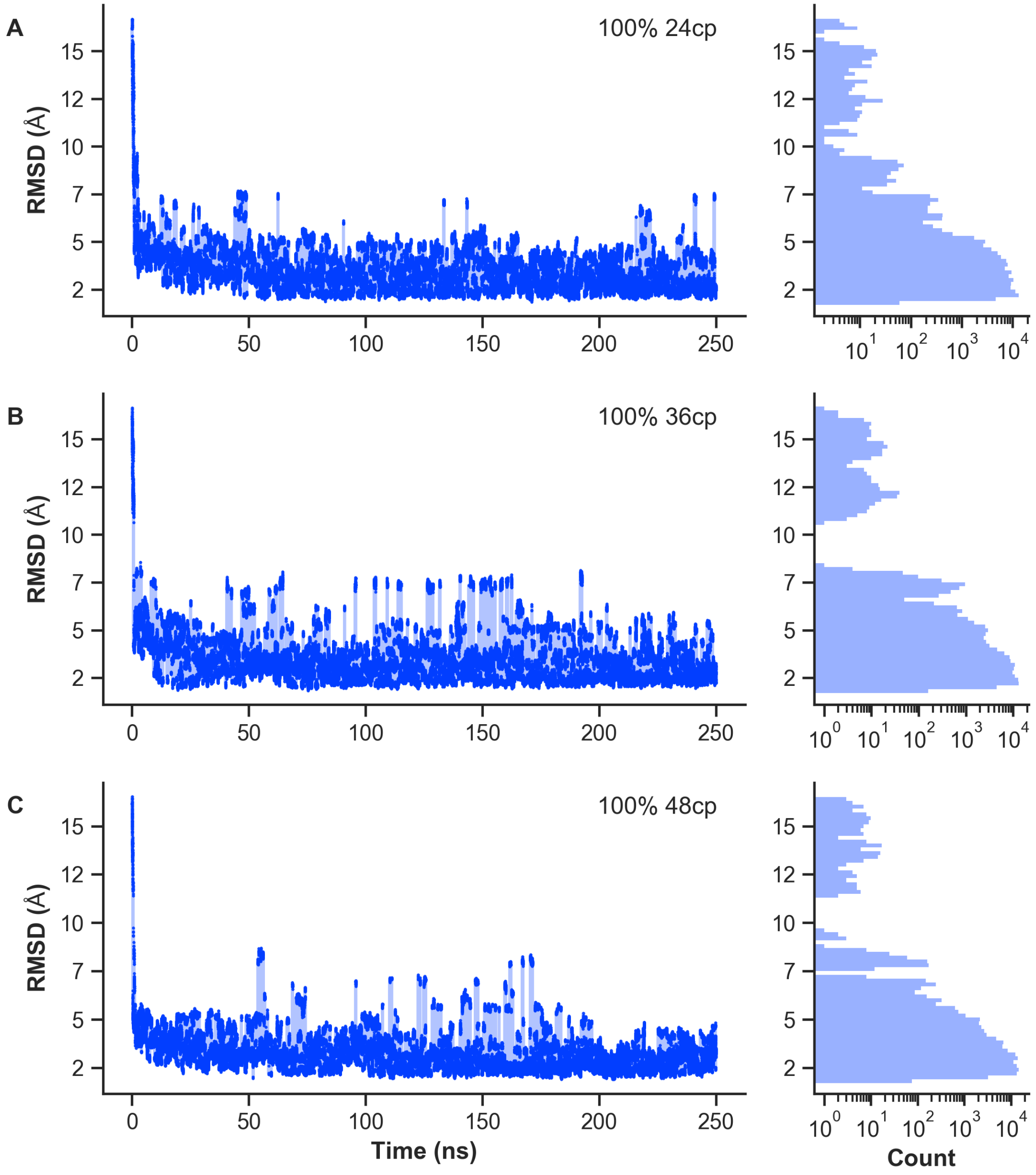

S26 Fig. RMSD curve and histogram of VHP REMD simulation.
Figure shows the backbone RMSD time evolution of the lowest-temperature replica at $T_0 = 300\,\mathrm{K}$ and the corresponding histogram with logarithmic count axis. (A) REMD simulation with 100% TPR and 24 native restraining contacts. (B) REMD simulation with 100% TPR and 36 native restraining contacts. (C) REMD simulation with 100% TPR and 48 native restraining contacts.

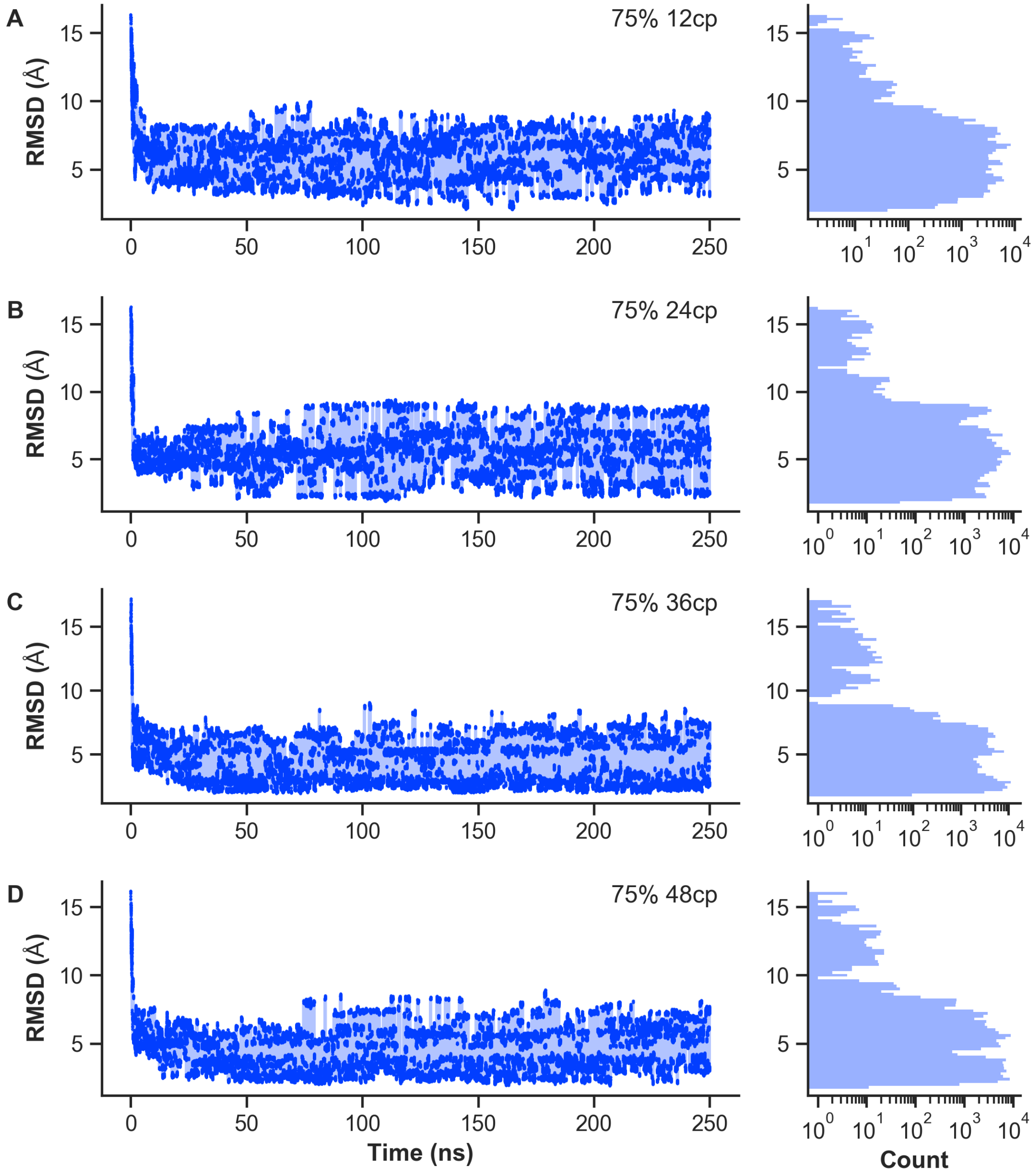

S27 Fig. RMSD curve and histogram of VHP REMD simulation.
Figure shows the backbone RMSD time evolution of the lowest-temperature replica at $T_0 = 300\,\mathrm{K}$ and the corresponding histogram with logarithmic count axis. (A) REMD simulation with 75% TPR and 12 restraining contacts (9 native, 3 non-native) (B) REMD simulation with 75% TPR and 24 restraining contacts (18 native, 6 non-native) (C) REMD simulation with 75% TPR and 36 restraining contacts (27 native, 9 non-native). (D) REMD simulation with 75% TPR and 48 restraining contacts (36 native, 12 non-native).

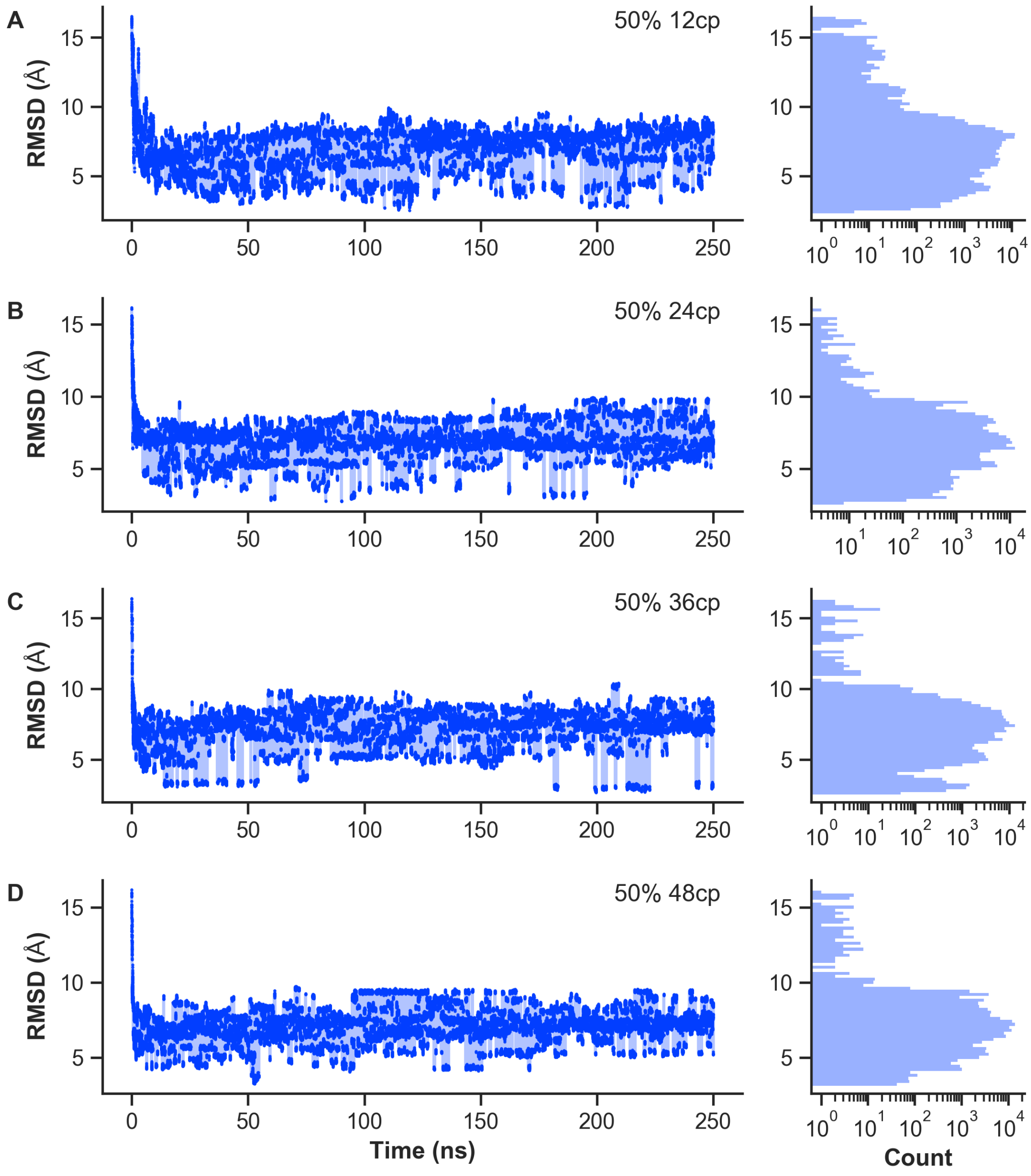

S28 Fig. RMSD curve and histogram of VHP REMD simulation.
Figure shows the backbone RMSD time evolution of the lowest-temperature replica at $T_0 = 300\,\mathrm{K}$ and the corresponding histogram with logarithmic count axis. (A) REMD simulation with 50% TPR and 12 restraining contacts (6 native, 6 non-native) (B) REMD simulation with 50% TPR and 24 restraining contacts (12 native, 12 non-native) (C) REMD simulation with 50% TPR and 36 restraining contacts (18 native, 18 non-native). (D) REMD simulation with 50% TPR and 48 restraining contacts (24 native, 24 non-native).

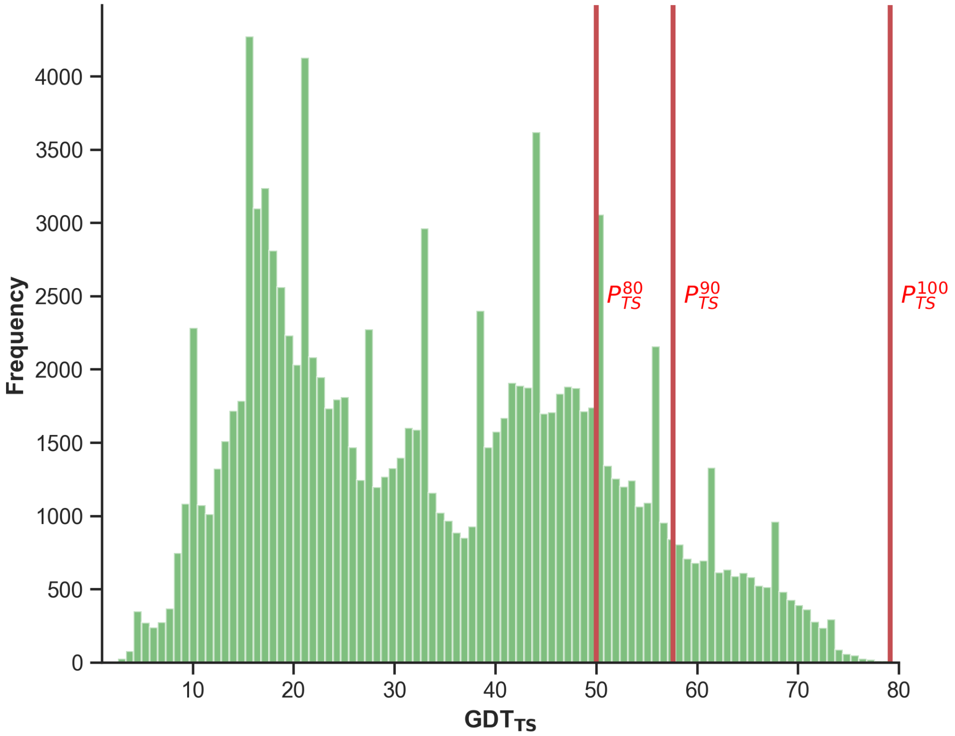

S29 Fig. Exemplary histogram with percentiles.
Histogram shows the frequencies of occurring global distance test total scores (GDT_{TS}) during the reference VHP REMD simulation. The 80th, 90th and 100th TS percentiles are indicated by red vertical lines.

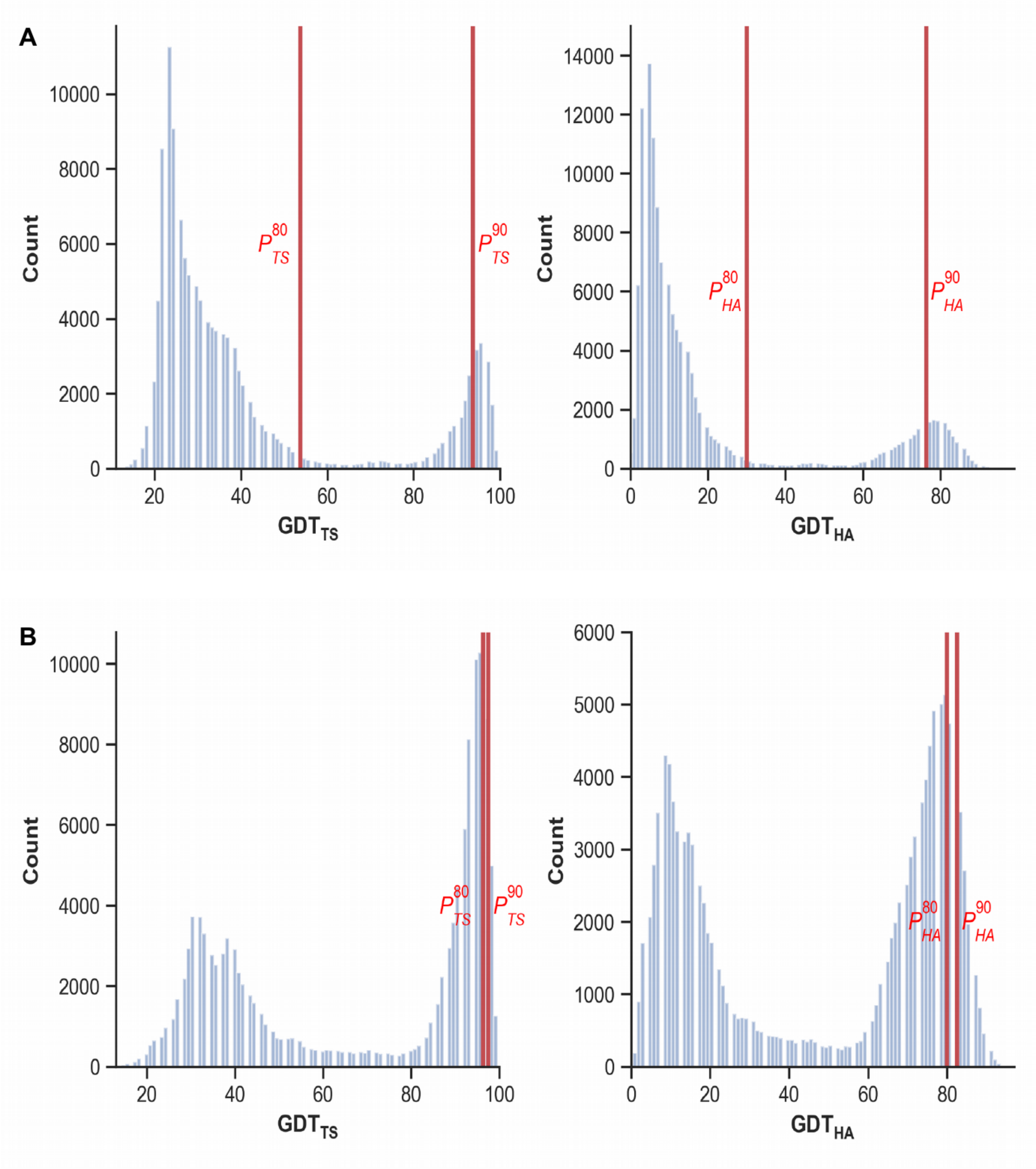

S30 Fig. GDT scores distribution of Trp-Cage REMD simulation (100% TPR Part 1).
Histograms show the counts of GDT_{TS} and GDT_{HA} scores of the lowest-temperature replica. Additionally, the 80th and 90th percentiles are displayed by red vertical lines. (A) reference REMD simulation without additional bias. (B) REMD simulation with 6 native contacts.

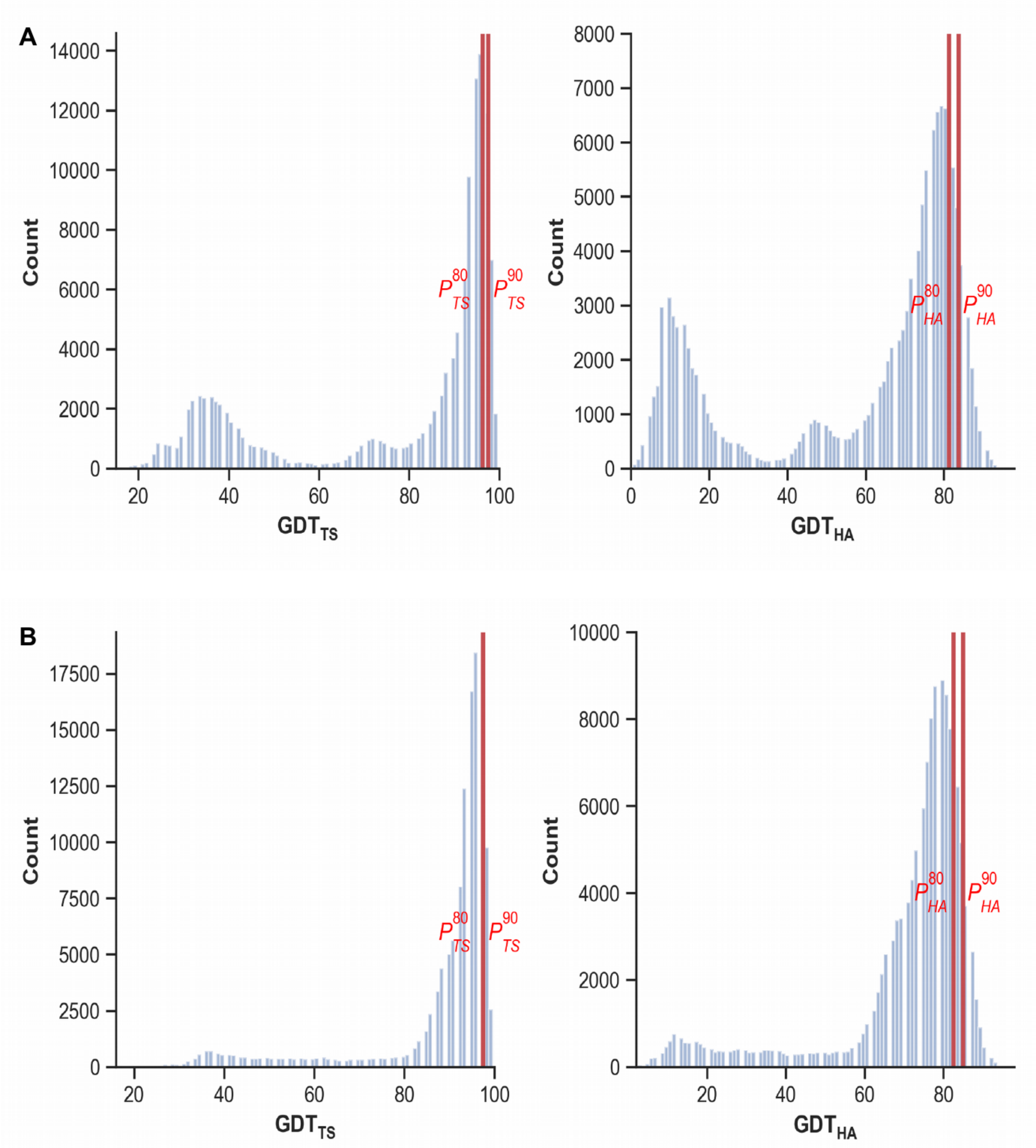

S31 Fig. GDT scores distribution of Trp-Cage REMD simulation (100% TPR Part 2).
Histograms show the counts of GDT_{TS} and GDT_{HA} scores. Additionally, the 80th and 90th percentiles are displayed by red vertical lines. (A) REMD simulation with 12 native contacts. (B) REMD simulation with 24 native contacts.

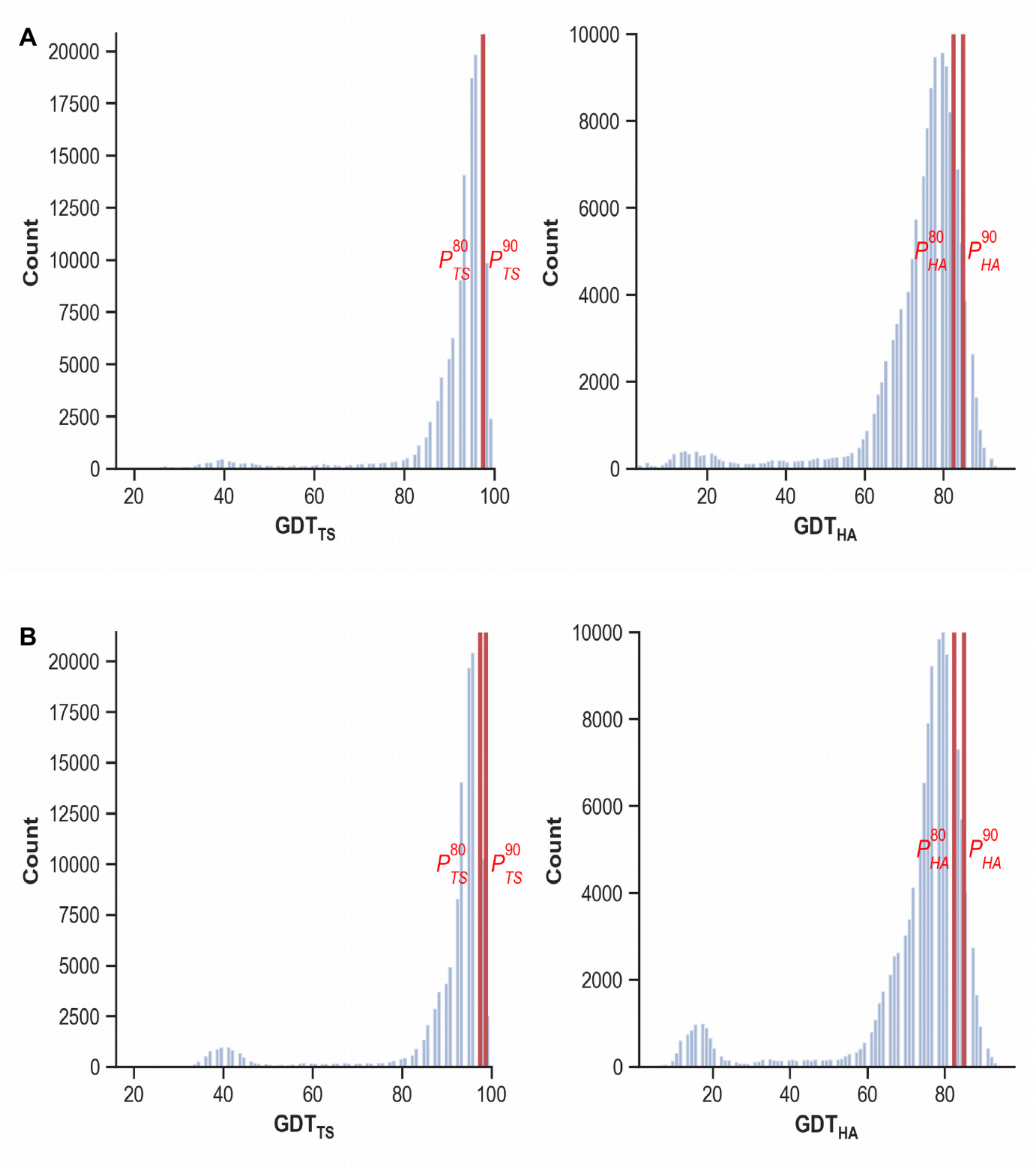

S32 Fig. GDT scores distribution of Trp-Cage REMD simulation (100% TPR Part 3).
Histograms show the counts of GDT_{TS} and GDT_{HA} scores of the lowest-temperature replica. Additionally, the 80th and 90th percentiles are displayed by red vertical lines. (A) REMD simulation with 36 native contacts. (B) REMD simulation with 48 native contacts.

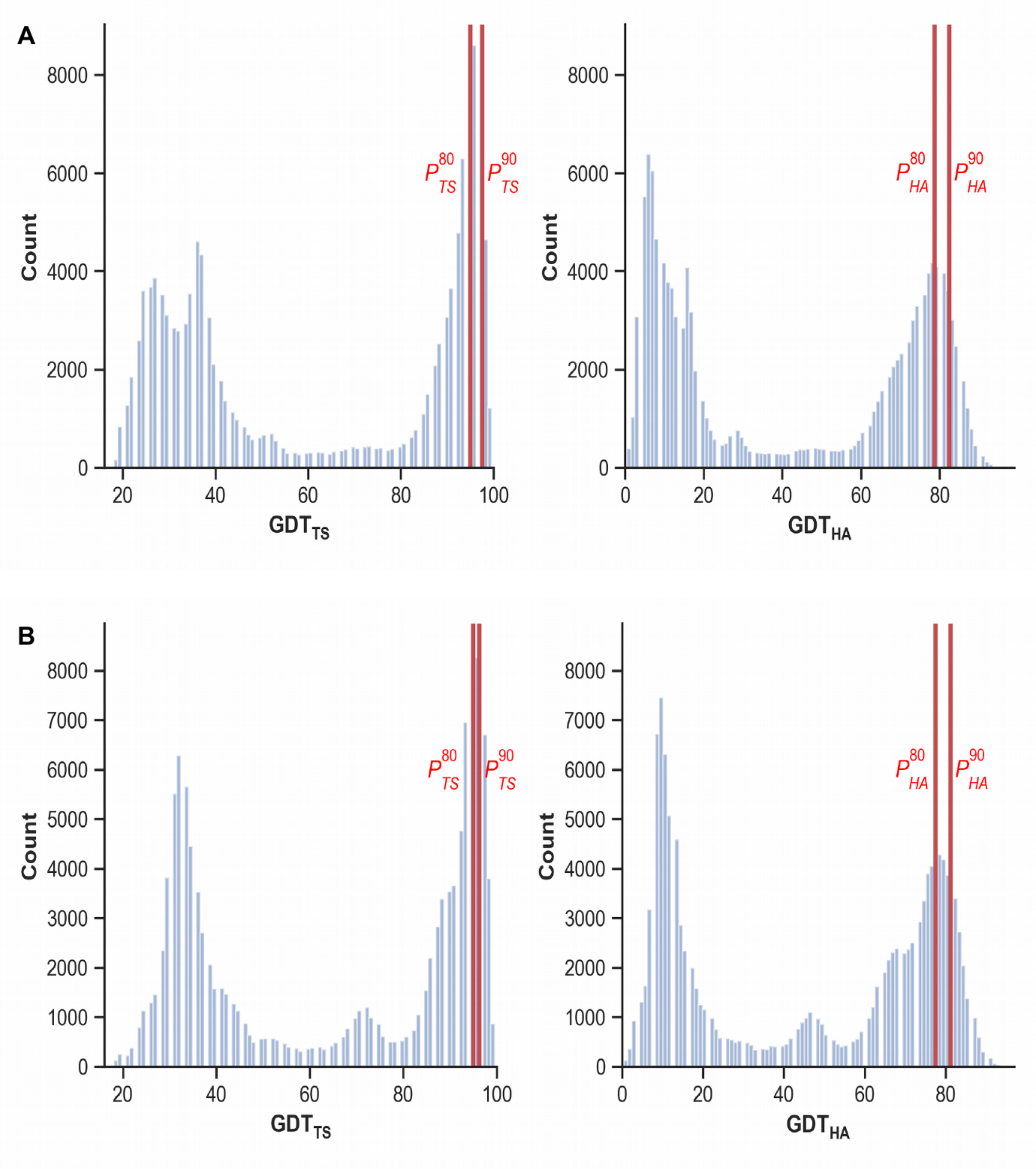

S33 Fig. GDT scores distribution of Trp-Cage REMD simulation (75% TPR Part 1).
Histograms show the counts of GDT_{TS} and GDT_{HA} scores of the lowest-temperature replica. Additionally, the 80th and 90th percentiles are displayed by red vertical lines. (A) REMD simulation with 12 contacts at 75% TPR. (B) REMD simulation with 24 contacts at 75% TPR.

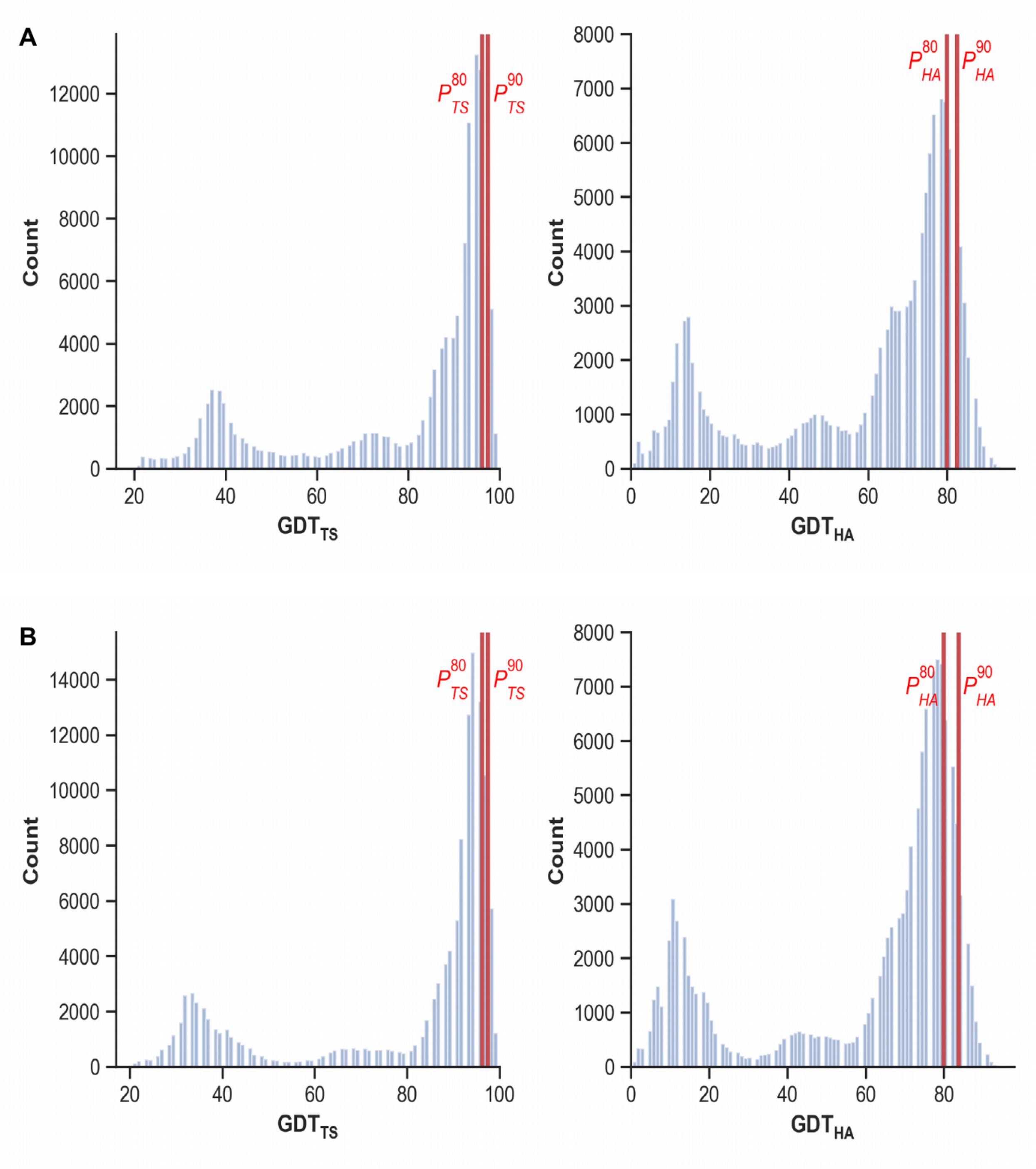

S34 Fig. GDT scores distribution of Trp-Cage REMD simulation (75% TPR Part 2).
Histograms show the counts of GDT_{TS} and GDT_{HA} scores of the lowest-temperature replica. Additionally, the 80th and 90th percentiles are displayed by red vertical lines. (A) REMD simulation with 36 contacts at 75% TPR. (B) REMD simulation with 48 contacts at 75% TPR.

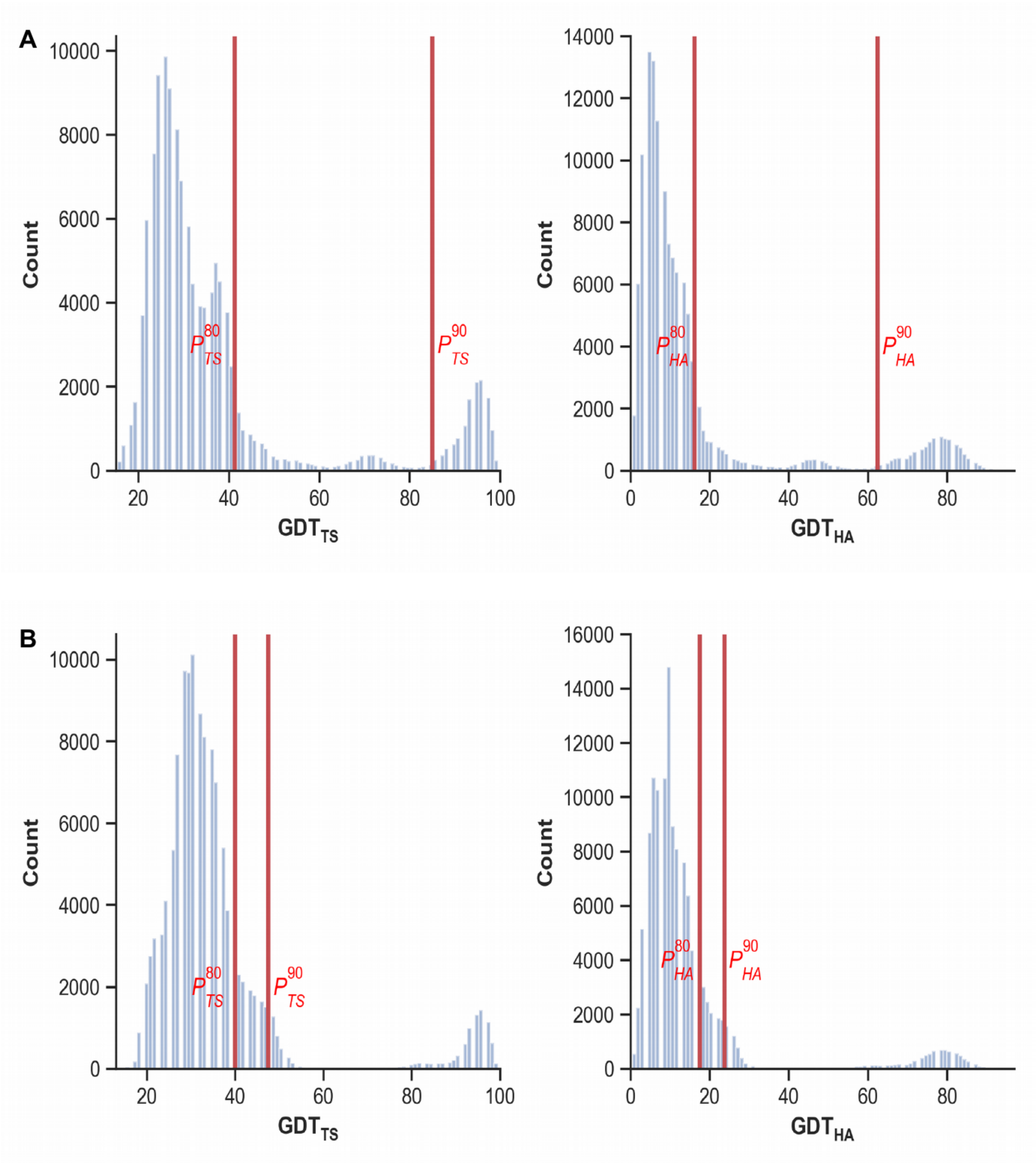

S35 Fig. GDT scores distribution of Trp-Cage REMD simulation (50% TPR Part 1).
Histograms show the counts of GDT_{TS} and GDT_{HA} scores of the lowest-temperature replica. Additionally, the 80th and 90th percentiles are displayed by red vertical lines. (A) REMD simulation with 12 contacts at 50% TPR. (B) REMD simulation with 24 contacts at 50% TPR.

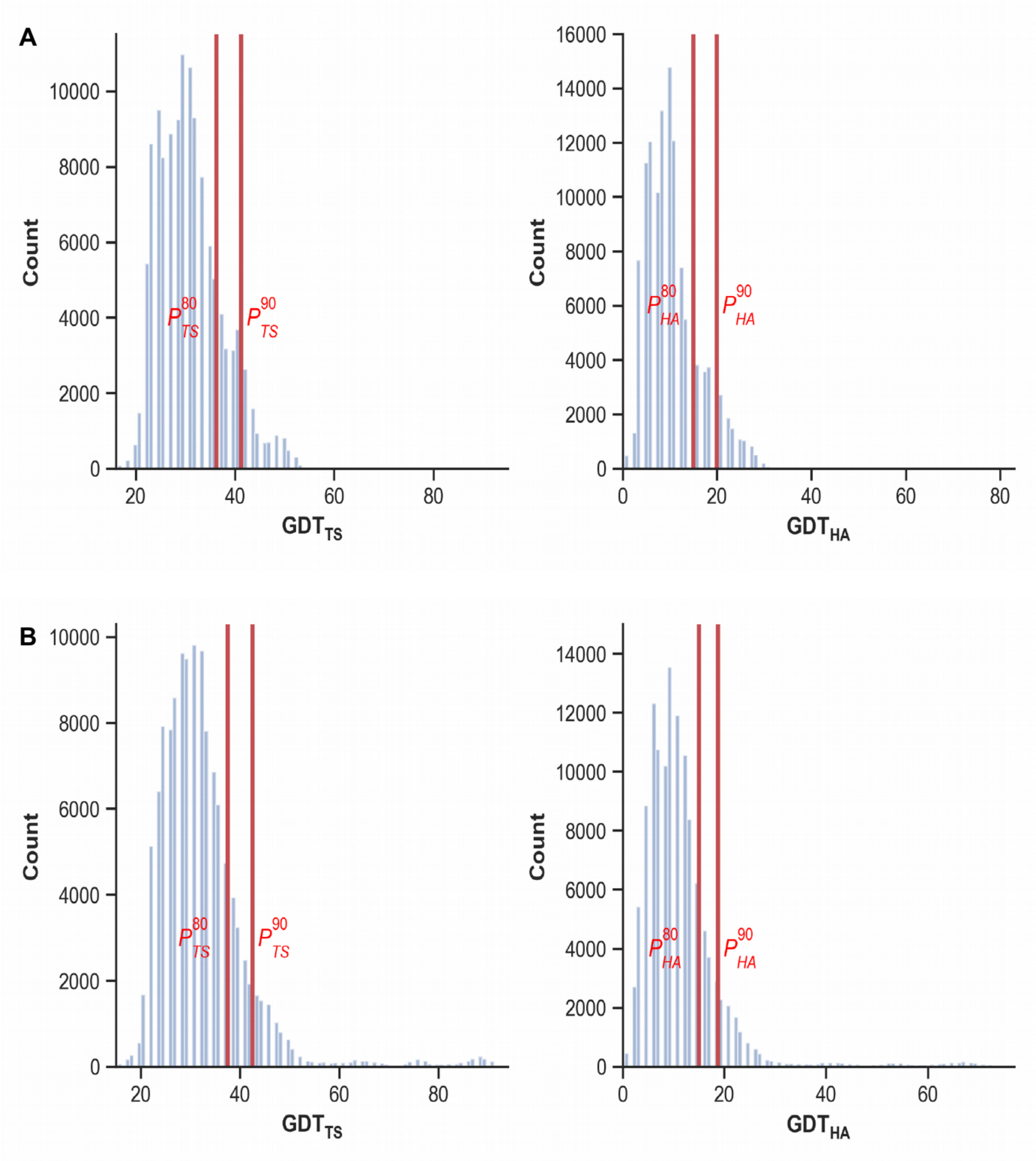

S36 Fig. GDT scores distribution of Trp-Cage REMD simulation (50% TPR Part 2).
Histograms show the counts of GDT_{TS} and GDT_{HA} scores of the lowest-temperature replica. Additionally, the 80th and 90th percentiles are displayed by red vertical lines. (A) REMD simulation with 36 contacts at 50% TPR. (B) REMD simulation with 48 contacts at 50% TPR.

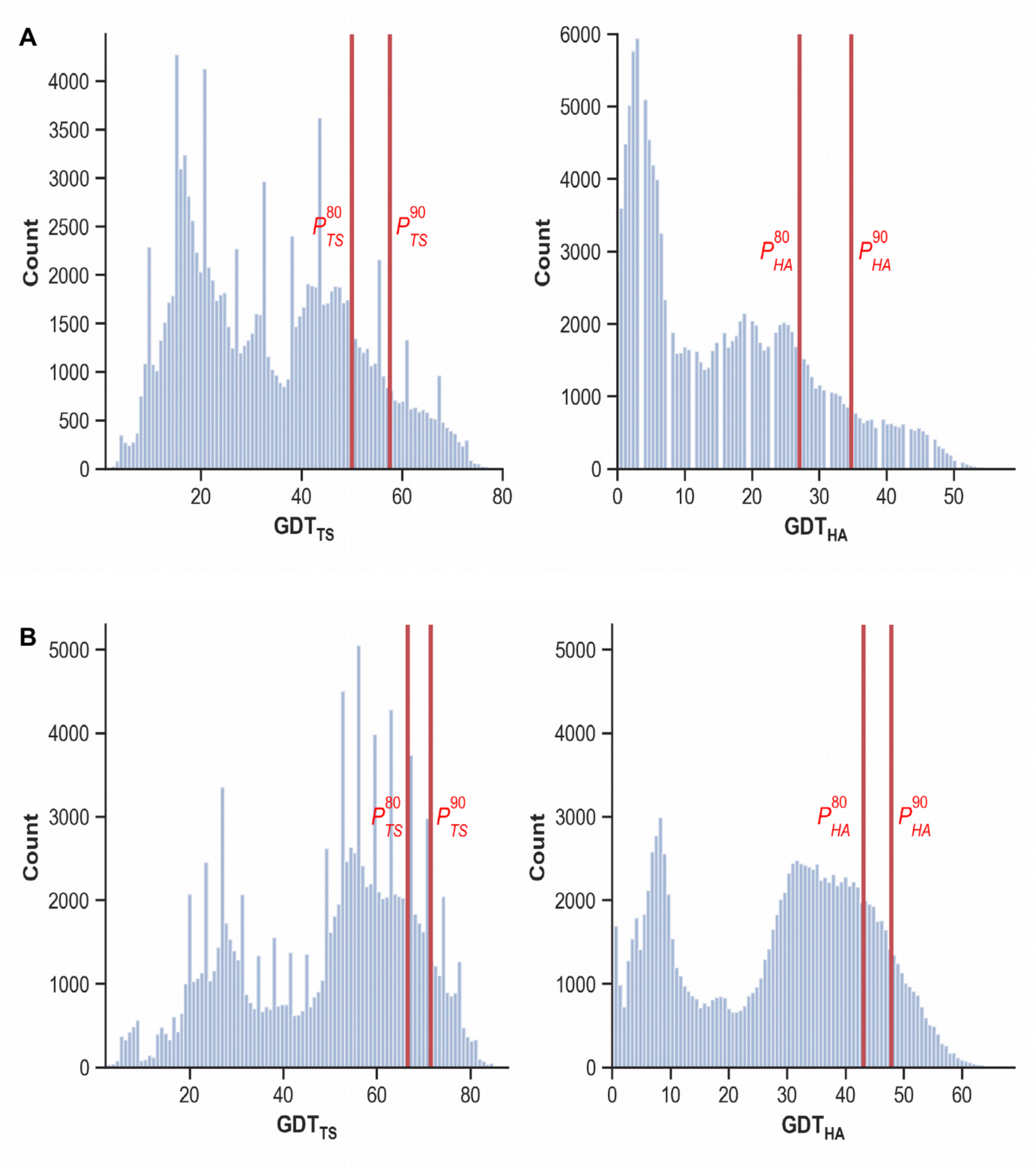

S37 Fig. GDT scores distribution of VHP REMD simulation (100% TPR Part 1).
Histograms show the counts of GDT_{TS} and GDT_{HA} scores of the lowest-temperature replica. Additionally, the 80th and 90th percentiles are displayed by red vertical lines. (A) reference REMD simulation without additional bias. (B) REMD simulation with 6 native contacts.

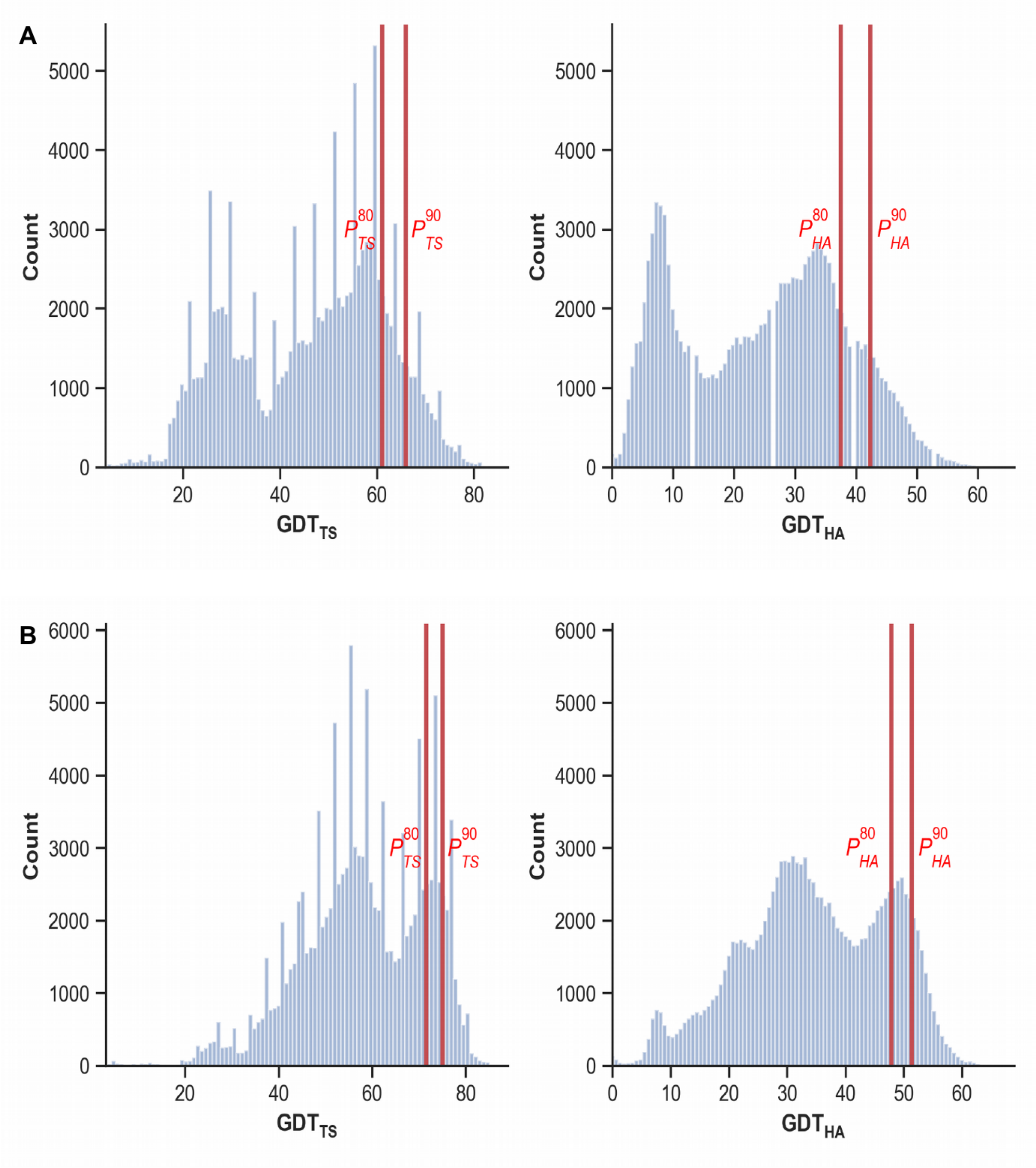

S38 Fig. GDT scores distribution of VHP REMD simulation (100% TPR Part 2).
Histograms show the counts of GDT_{TS} and GDT_{HA} scores of the lowest-temperature replica. Additionally, the 80th and 90th percentiles are displayed by red vertical lines. (A) REMD simulation with 12 native contacts. (B) REMD simulation with 24 native contacts.

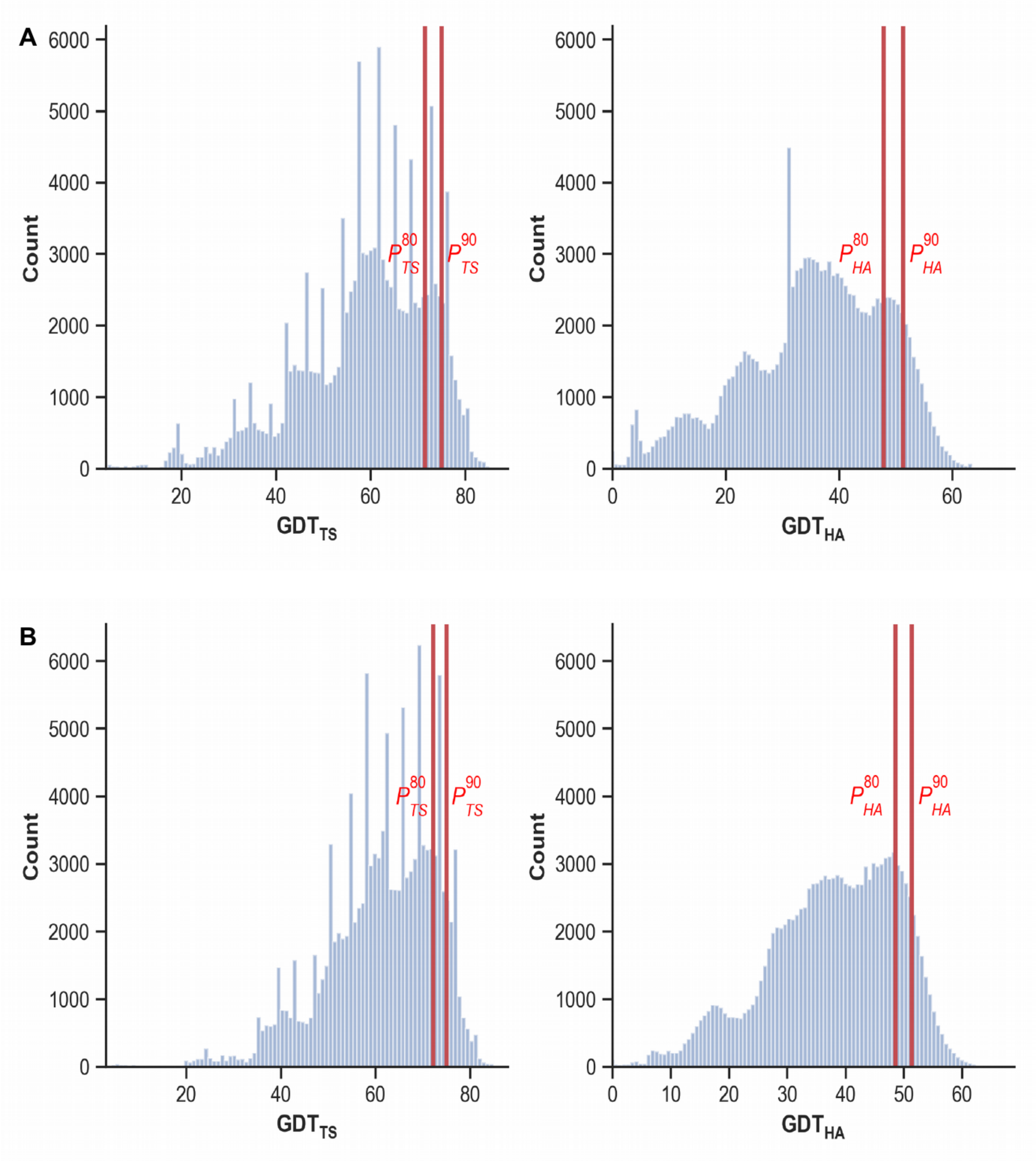

S39 Fig. GDT scores distribution of VHP REMD simulation (100% TPR Part 3).
Histograms show the counts of GDT_{TS} and GDT_{HA} scores of the lowest-temperature replica. Additionally, the 80th and 90th percentiles are displayed by red vertical lines. (A) REMD simulation with 36 native contacts. (B) REMD simulation with 48 native contacts.

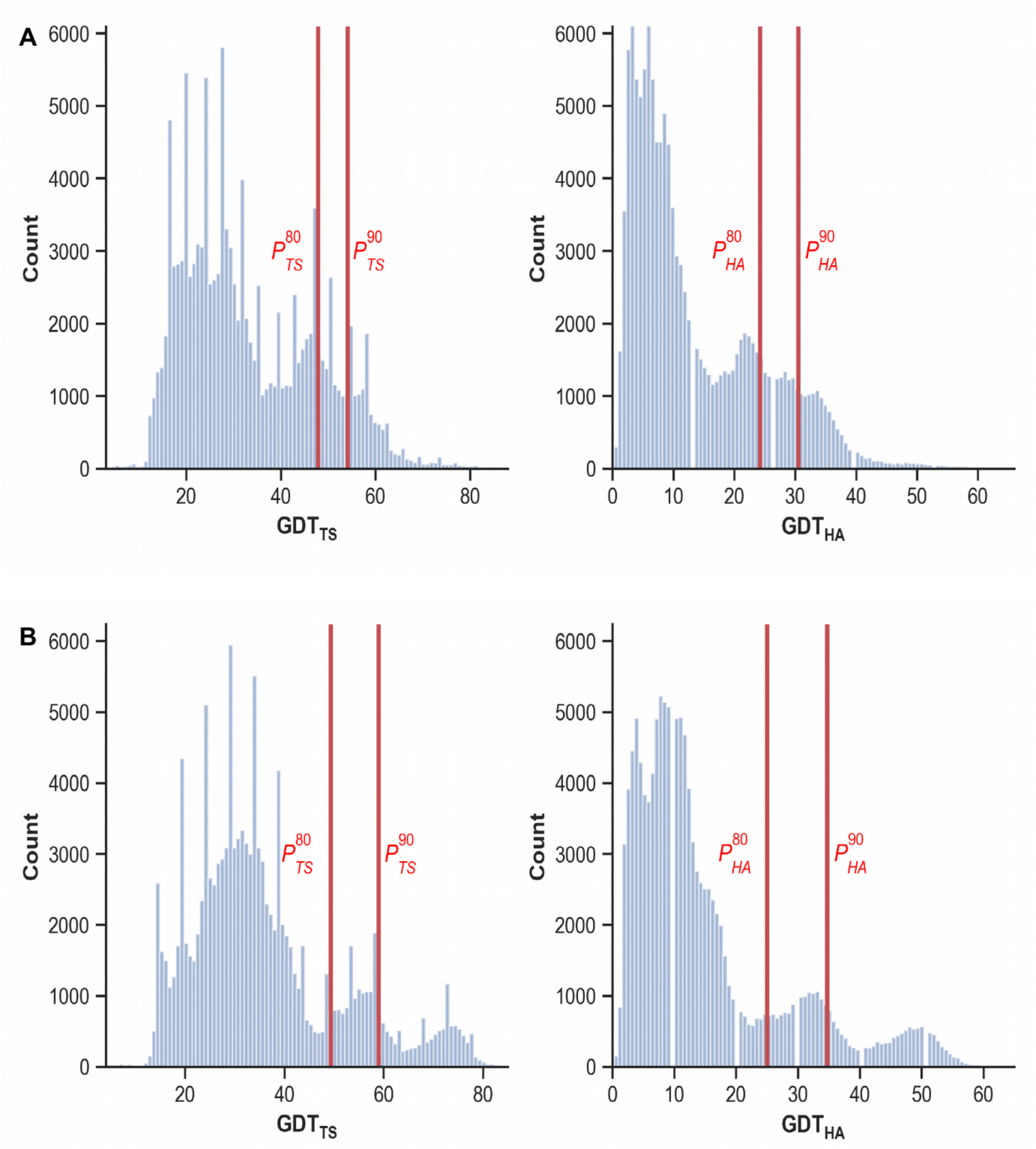

S40 Fig. GDT scores distribution of VHP REMD simulation (75% TPR Part 1).
Histograms show the counts of GDT_{TS} and GDT_{HA} scores of the lowest-temperature replica. Additionally, the 80th and 90th percentiles are displayed by red vertical lines. (A) REMD simulation with 12 contacts at 75% TPR. (B) REMD simulation with 24 contacts at 75% TPR.

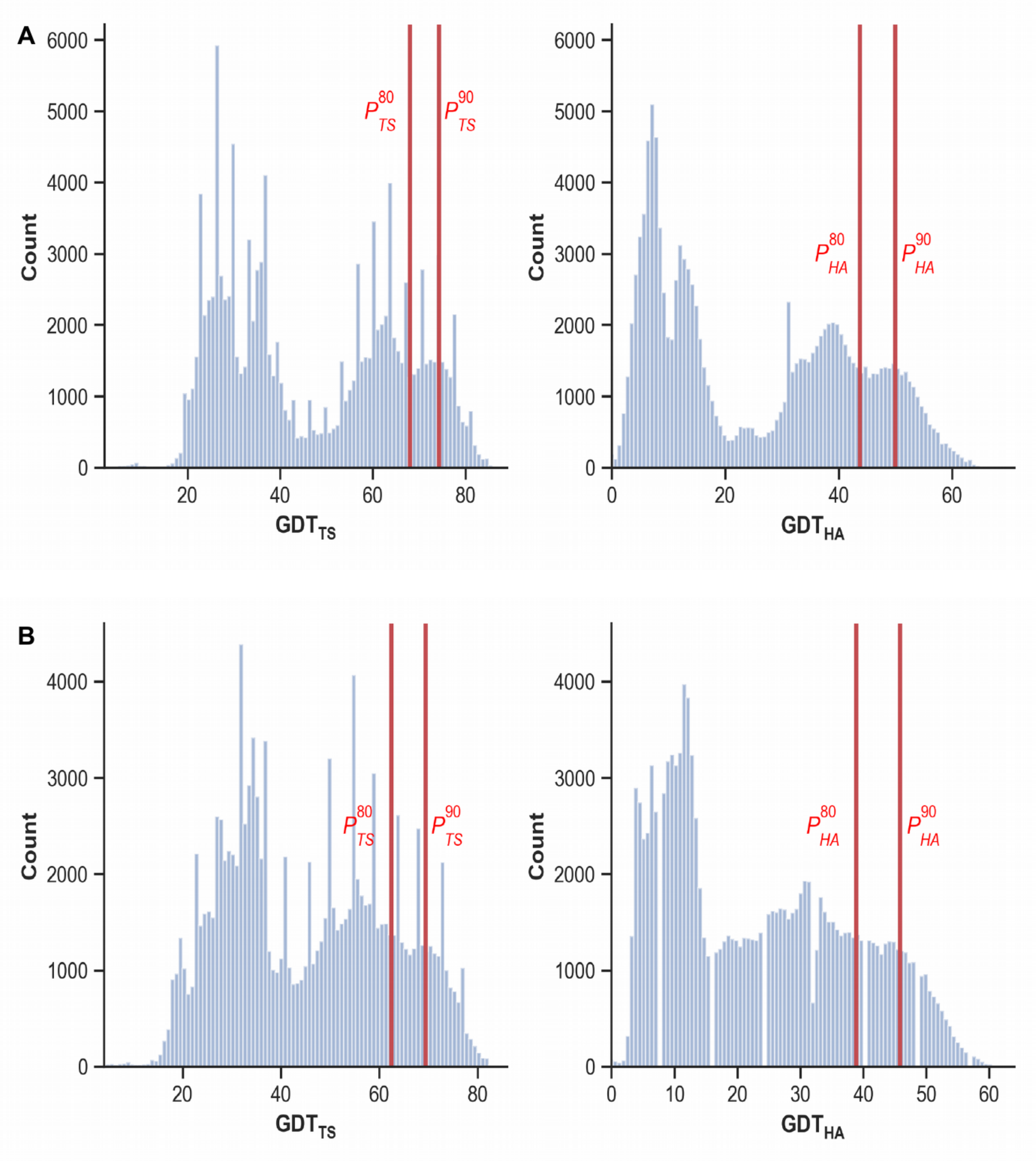

S41 Fig. GDT scores distribution of VHP REMD simulation (75% TPR Part 2).
Histograms show the counts of GDT_{TS} and GDT_{HA} scores of the lowest-temperature replica. Additionally, the 80th and 90th percentiles are displayed by red vertical lines. (A) REMD simulation with 36 contacts at 75% TPR. (B) REMD simulation with 48 contacts at 75% TPR.

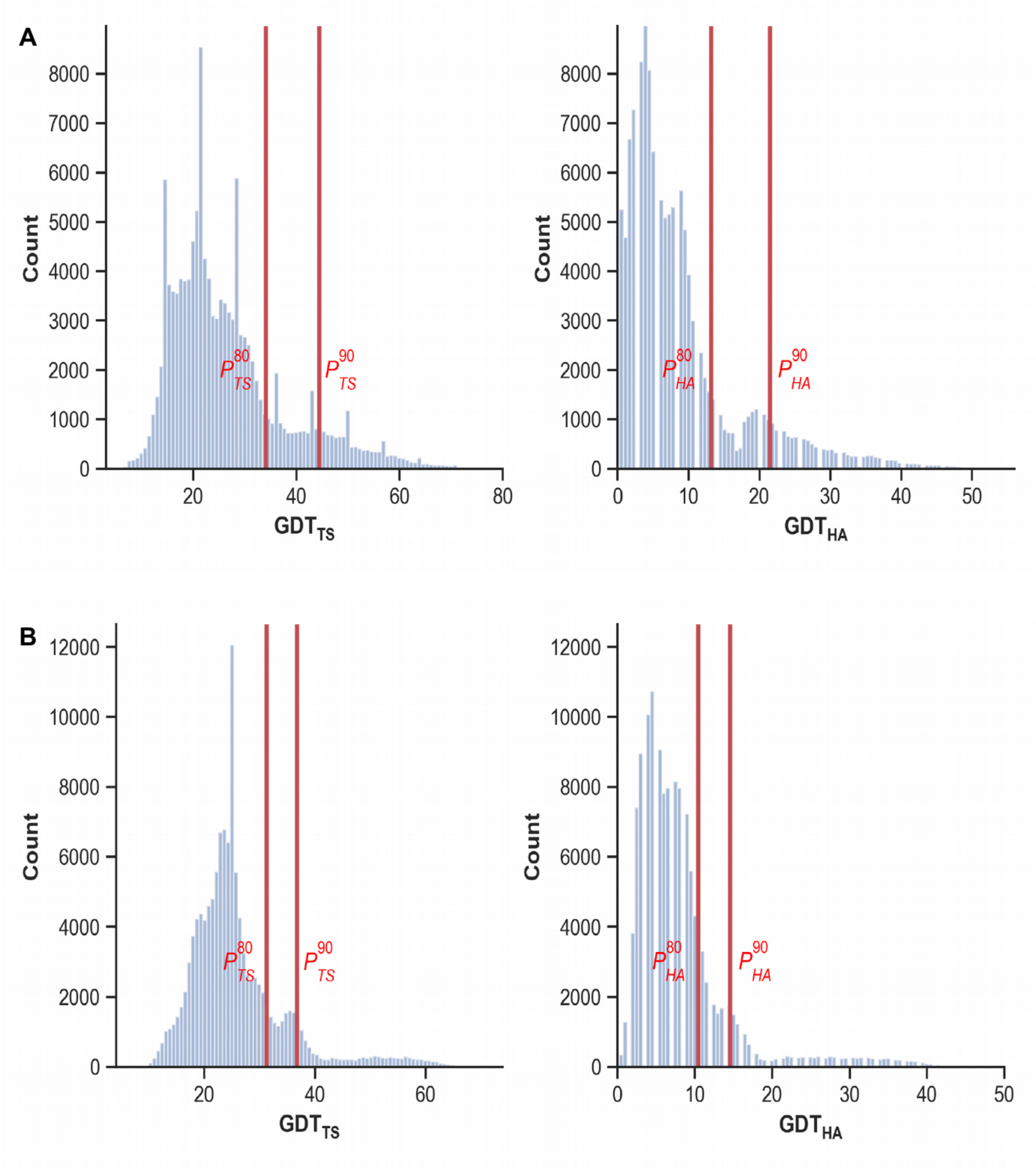

S42 Fig. GDT scores distribution of VHP REMD simulation (50% TPR Part 1).
Histograms show the counts of GDT_{TS} and GDT_{HA} scores of the lowest-temperature replica. Additionally, the 80th and 90th percentiles are displayed by red vertical lines. (A) REMD simulation with 12 contacts at 50% TPR. (B) REMD simulation with 24 contacts at 50% TPR.

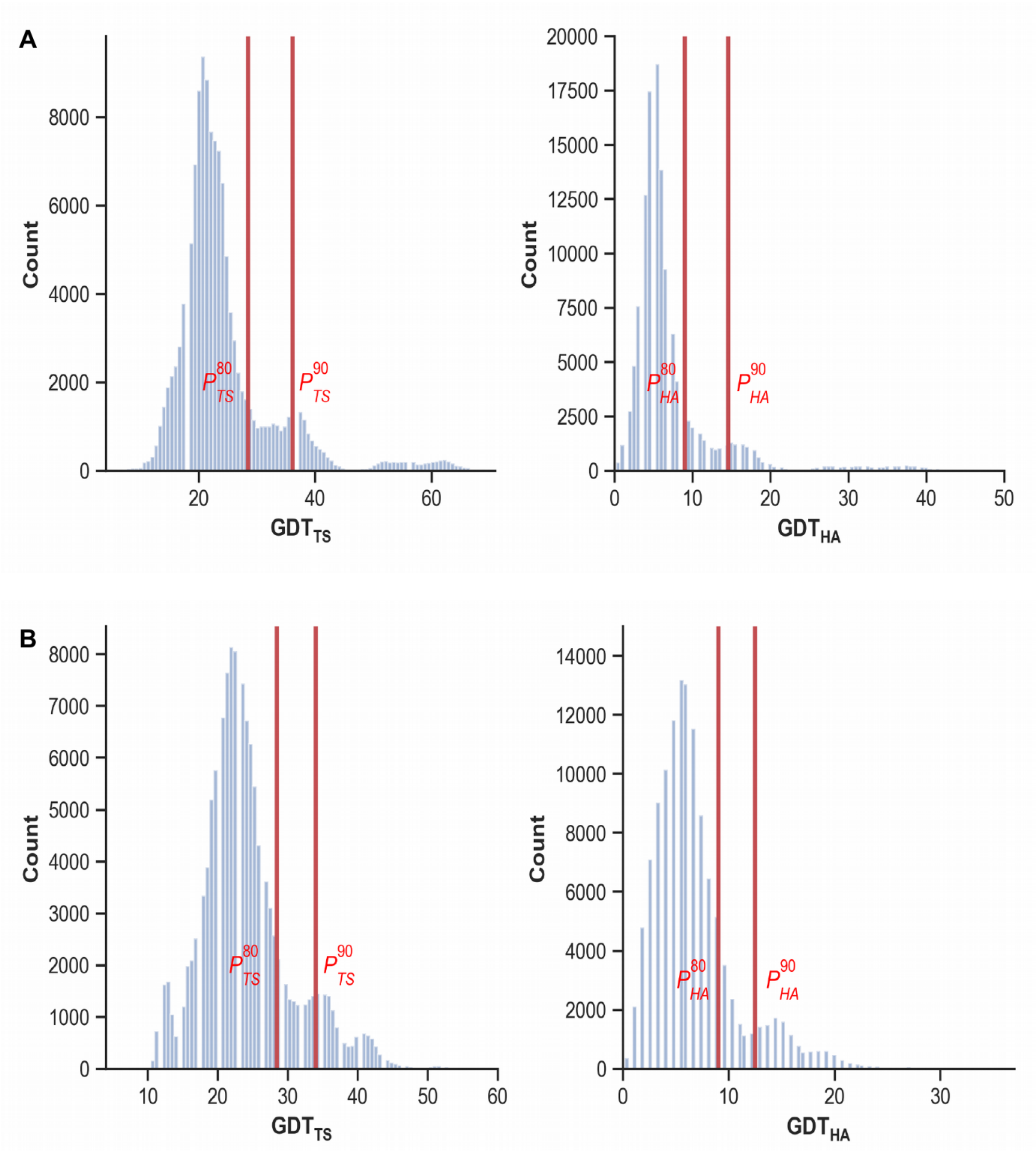

S43 Fig. GDT scores distribution of VHP REMD simulation (50% TPR Part 2).
Histograms show the counts of GDT_{TS} and GDT_{HA} scores of the lowest-temperature replica. Additionally, the 80th and 90th percentiles are displayed by red vertical lines. (A) REMD simulation with 36 contacts at 50% TPR. (B) REMD simulation with 48 contacts at 50% TPR.

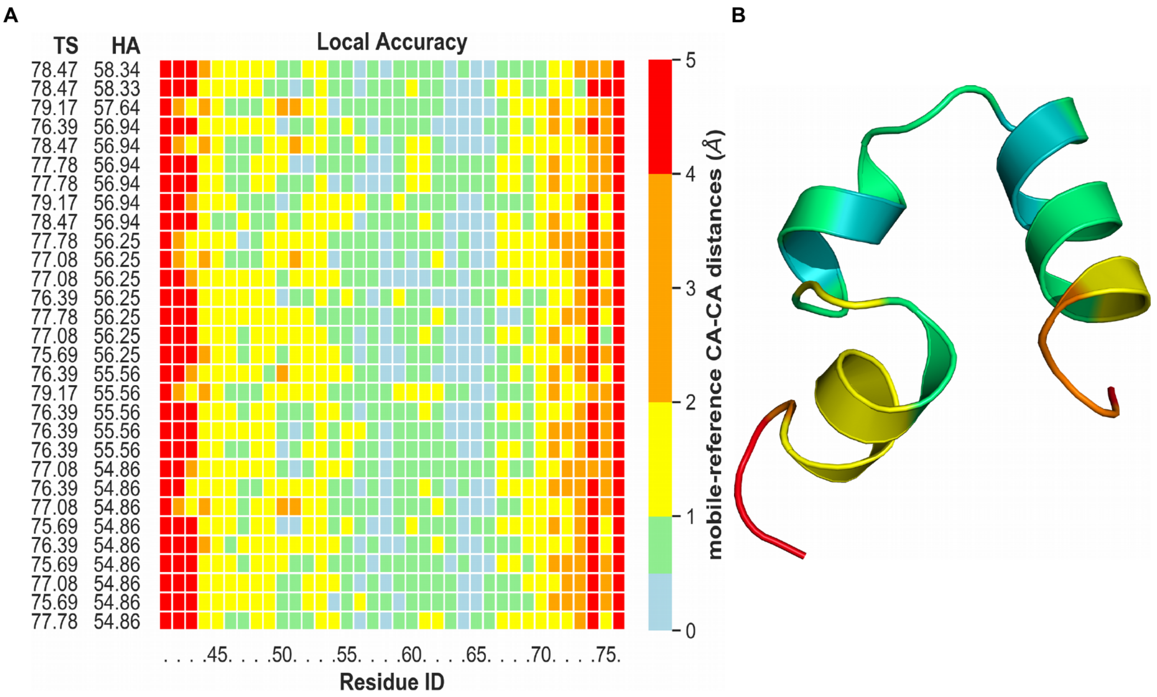

S44 Fig. Local accuracy of VHP structures (reference REMD).
(A) Displayed are the best observed structures ranked by high-accuracy (HA) score and color-coded based on the C_α-C_α distance to visualize the local accuracy. (B) Best observed tertiary structure of VHP corresponding to the first line of subfigure A.

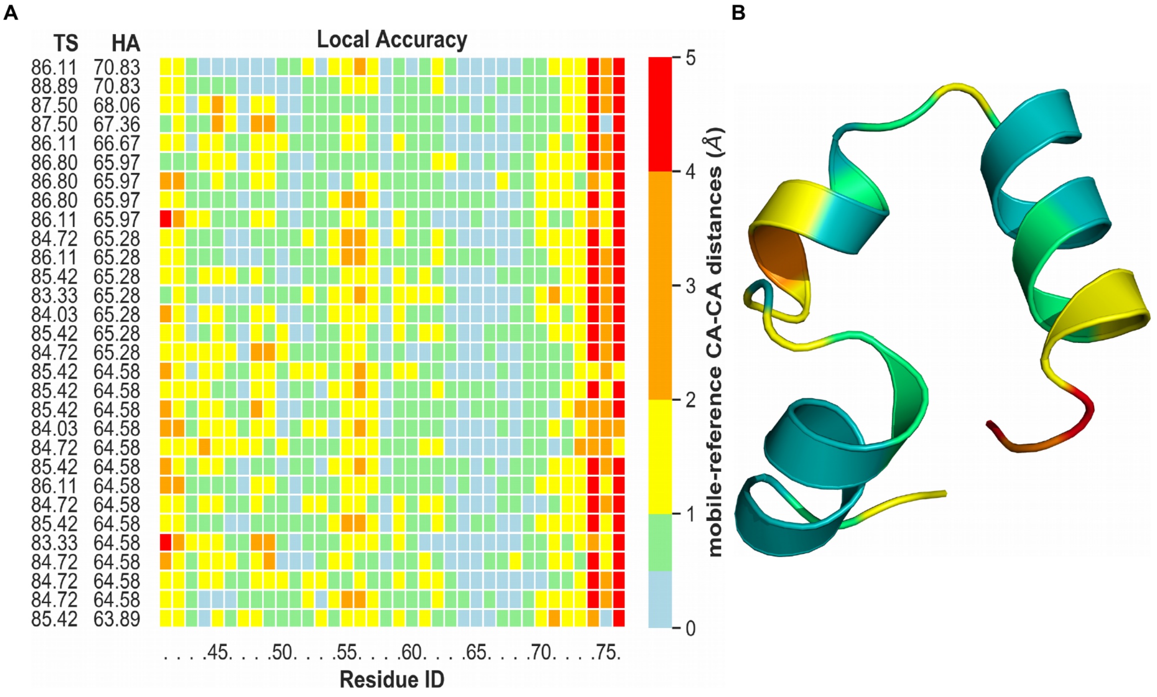

S45 Fig. Local accuracy of VHP structures (36 contacts, 100% TPR).
(A) Displayed are the best observed structures ranked by high-accuracy (HA) score and color-coded based on the C_α-C_α distance to visualize the local accuracy. (B) Best observed tertiary structure of VHP corresponding to the first line of subfigure A.

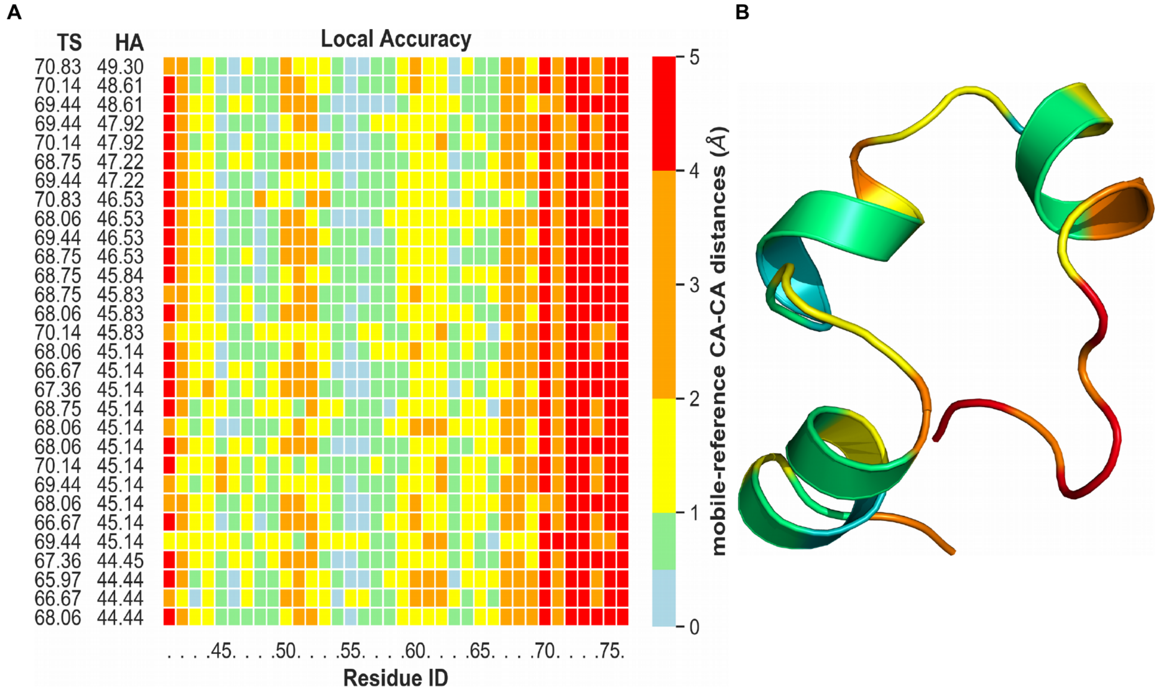

S46 Fig. Local accuracy of VHP structures (36 contacts, 50% TPR).

(A) Displayed are the best observed structures ranked by high-accuracy (HA) score and color-coded based on the C_α-C_α distance to visualize the local accuracy. (B) Best observed tertiary structure of VHP corresponding to the first line of subfigure A.
