# Supplementary material for: Including residual contact information into replica-exchange MD simulations significantly enriches native-like conformations
Source: PLoS One. 2020 Nov 16;15(11):e0242072. doi: 10.1371/journal.pone.0242072 (PMC7668583; doi:10.1371/journal.pone.0242072)
Supplement: S2 Appendix — (PDF) [file pone.0242072.s002.pdf]

S2 Appendix. Teperature distributions of REMD simulations.

Trp-Cage:

```
REMD Temperature Distribution:
T_0 = 300 K ; DELTA = T_0 * (exp(k*i)-exp(k*(i-1)))
T_i = T_(i-1) + a_i * DELTA

Chosen Parameter:
k =0.0115
a0=1.00 for i =  0..9
a1=1.04 for i =  10..19
a2=1.08 for i =  20..29
a3=1.12 for i =  30..39
a4=1.16 for i =  40..49
a5=1.20 for i =  50..59

Temperatures:
300.00, 303.47, 306.98, 310.53, 314.12, 317.76, 321.43, 325.15, 328.91,
    332.71, 336.72, 340.76, 344.86, 349.00, 353.19, 357.43, 361.72,
    366.06, 370.45, 374.88, 379.55, 384.26, 389.04, 393.86, 398.74,
    403.68, 408.68, 413.73, 418.84, 424.01, 429.44, 434.93, 440.48,
    446.09, 451.77, 457.52, 463.33, 469.21, 475.16, 481.17, 487.48,
    493.85, 500.30, 506.83, 513.43, 520.10, 526.86, 533.69, 540.60,
    547.59, 554.90, 562.30, 569.79, 577.36, 585.02, 592.77, 600.61,
    608.54, 616.56, 624.67,
```

VHP:

```
REMD Temperature Distribution:
T_0 = 300 K ; DELTA = T_0 * (exp(k*i)-exp(k*(i-1)))
T_i = T_(i-1) + a_i * DELTA

Chosen Parameter:
k =0.0065
a0=1.00 for i =  0..9
a1=1.04 for i =  10..19
a2=1.08 for i =  20..29
a3=1.12 for i =  30..39
a4=1.16 for i =  40..49
a5=1.20 for i =  50..59
a6=1.24 for i =  60..69
a7=1.28 for i =  70..79
a8=1.32 for i =  80..89
a9=1.36 for i =  90..99

Temperatures:
300.00, 301.96, 303.93, 305.91, 307.90, 309.91, 311.93, 313.97, 316.01,
    318.07, 320.23, 322.40, 324.59, 326.79, 329.00, 331.23, 333.47,
    335.73, 338.00, 340.29, 342.68, 345.09, 347.51, 349.95, 352.40,
    354.87, 357.35, 359.86, 362.37, 364.91, 367.55, 370.22, 372.90,
    375.60, 378.31, 381.04, 383.79, 386.56, 389.35, 392.16, 395.08,
    398.02, 400.99, 403.97, 406.97, 409.99, 413.03, 416.09, 419.17,
    422.27, 425.50, 428.75, 432.02, 435.31, 438.62, 441.96, 445.31,
    448.69, 452.09, 455.52, 459.08, 462.66, 466.26, 469.89, 473.55,
    477.23, 480.93, 484.65, 488.40, 492.18, 496.10, 500.04, 504.02,
    508.02, 512.04, 516.09, 520.17, 524.27, 528.40, 532.56, 536.88,
    541.22, 545.59, 549.99, 554.42, 558.88, 563.37, 567.88, 572.43,
    577.00, 581.75, 586.53, 591.33, 596.17, 601.04, 605.94, 610.88,
    615.84, 620.84, 625.87,
```
