# Supplementary material for: Including residual contact information into replica-exchange MD simulations significantly enriches native-like conformations
Source: PLoS One. 2020 Nov 16;15(11):e0242072. doi: 10.1371/journal.pone.0242072 (PMC7668583; doi:10.1371/journal.pone.0242072)
Supplement: S3 Appendix — (PDF) [file pone.0242072.s003.pdf]

S3 Appendix. Sample mdp file for REMD simulations.

```
; Run parameters
integrator  = md                ; leap-frog integrator
dt          = 0.002             ; 2 fs
nsteps      = 125000000         ; nsteps * dt = 250 ns
; Output control
nstxout               = 10000     ; save coordinates every 20 ps
nstvout               = 10000     ; save velocities  every 20 ps
nstenergy             = 1000      ; save energies    every  2 ps
nstlog                = 1000      ; save log file    every  2 ps
nstxout-compressed    = 1000      ; save compr.coord every  2 ps
compressed-x-grps     = Protein   ; replaces xtc-grps

; Bond parameters
continuation          = no        ; first dynamics run
constraint_algorithm= lincs       ; holonomic constraints
constraints           = all-bonds ; all bonds (even heavy atom-H bonds) constrained
lincs_iter            = 1         ; accuracy of LINCS
lincs_order           = 4         ; also related to accuracy

; Neighborsearching
cutoff-scheme   = Verlet
ns_type         = grid      ; search neighboring grid cells
nstlist         = 10        ; 20 fs, largely irrelevant with Verlet
rcoulomb        = 1.0       ; short-range electrostatic cutoff (in nm)
rvdw            = 1.0       ; short-range van der Waals cutoff (in nm)

; Electrostatics
coulombtype     = PME       ; Particle Mesh Ewald for long-range electrostatics
pme_order       = 4         ; cubic interpolation
fourierspacing  = 0.16      ; grid spacing for FFT

; Temperature coupling is on
tcoupl  = V-rescale                ; modified Berendsen thermostat
tc-grps = Protein Non-Protein      ; two coupling groups - more accurate
tau_t   = 0.1   0.1                ; time constant, in ps
ref_t   = 300   300                ; reference temperature, one for each group, in K

; Pressure coupling
pcoupl = no

; Periodic boundary conditions
pbc = xyz    ; 3-D PBC
; Dispersion correction

DispCorr = EnerPres ; account for cut-off vdW scheme

; Velocity generation
gen_vel  = yes  ; assign velocities from Maxwell distribution
gen_temp = 300  ; temperature for Maxwell distribution
gen_seed = -1   ; generate a random seed
```
