# Supplementary material for: Including residual contact information into replica-exchange MD simulations significantly enriches native-like conformations
Source: PLoS One. 2020 Nov 16;15(11):e0242072. doi: 10.1371/journal.pone.0242072 (PMC7668583; doi:10.1371/journal.pone.0242072)
Supplement: S4 Appendix — (PDF) [file pone.0242072.s004.pdf]

## S4 Appendix. Sample mdp file for MD simulations.

```
; Run parameters
integrator  = md              ; leap-frog integrator
dt          = 0.002           ; 2 fs
nsteps      = 250000000       ; nsteps * dt = 500 ns

; Output control
nstxout              = 10000     ; save coordinates every 20 ps
nstvout              = 10000     ; save velocities  every 20 ps
nstenergy            = 10000     ; save energies    every 20 ps
nstlog               = 10000     ; save log file    every 20 ps
nstxout-compressed   = 1000      ; save compr.coord every  2 ps
compressed-x-grps    = Protein   ; replaces xtc-grps

; Bond parameters
continuation         = yes       ; Restarting after NPT
constraint_algorithm= lincs      ; holonomic constraints
constraints          = all-bonds ; all bonds (even heavy atom-H bonds) constrained
lincs_iter           = 1         ; accuracy of LINCS
lincs_order          = 4         ; also related to accuracy

; Neighborsearching
cutoff-scheme   = Verlet
ns_type         = grid      ; search neighboring grid cells
nstlist         = 10        ; 20 fs, largely irrelevant with Verlet
rcoulomb        = 1.0       ; short-range electrostatic cutoff (in nm)
rvdw            = 1.0       ; short-range van der Waals cutoff (in nm)

; Electrostatics
coulombtype     = PME       ; Particle Mesh Ewald for long-range electrostatics
pme_order       = 4         ; cubic interpolation
fourierspacing  = 0.16      ; grid spacing for FFT

; Temperature coupling is on
tcoupl  = V-rescale              ; modified Berendsen thermostat
tc-grps = Protein Non-Protein    ; two coupling groups - more accurate
tau_t   = 0.1   0.1              ; time constant, in ps
ref_t   = 300   300              ; reference temperature, one for each group, in K

; Pressure coupling is on
pcoupl          = Parrinello-Rahman ; Pressure coupling on in NPT
pcoupltype      = isotropic         ; uniform scaling of box vectors
tau_p           = 2.0               ; time constant, in ps
ref_p           = 1.0               ; reference pressure, in bar
compressibility = 4.5e-5            ; isothermal compressibility of water, bar^-1

; Periodic boundary conditions
pbc = xyz   ; 3-D PBC

; Dispersion correction
DispCorr = EnerPres ; account for cut-off vdW scheme

; Velocity generation
gen_vel     = no    ; Velocity generation is off (continuation is on)
```
